# CRYPTOCHROMES promote daily protein homeostasis

**DOI:** 10.15252/embj.2021108883

**Published:** 2021-11-29

**Authors:** David C S Wong, Estere Seinkmane, Aiwei Zeng, Alessandra Stangherlin, Nina M Rzechorzek, Andrew D Beale, Jason Day, Martin Reed, Sew Y Peak‐Chew, Christine T Styles, Rachel S Edgar, Marrit Putker, John S O’Neill

**Affiliations:** ^1^ MRC Laboratory of Molecular Biology Cambridge UK; ^2^ Department of Earth Sciences University of Cambridge Cambridge UK; ^3^ Department of Infectious Diseases Imperial College London UK; ^4^ Present address: Crown Bioscience Utrecht the Netherlands

**Keywords:** circadian rhythm, clock mutant, CRYPTOCHROME, protein homeostasis, proteotoxic stress, Metabolism, Post-translational Modifications & Proteolysis, Proteomics

## Abstract

The daily organisation of most mammalian cellular functions is attributed to circadian regulation of clock‐controlled protein expression, driven by daily cycles of CRYPTOCHROME‐dependent transcriptional feedback repression. To test this, we used quantitative mass spectrometry to compare wild‐type and CRY‐deficient fibroblasts under constant conditions. In CRY‐deficient cells, we found that temporal variation in protein, phosphopeptide, and K^+^ abundance was at least as great as wild‐type controls. Most strikingly, the extent of temporal variation within either genotype was much smaller than overall differences in proteome composition between WT and CRY‐deficient cells. This proteome imbalance in CRY‐deficient cells and tissues was associated with increased susceptibility to proteotoxic stress, which impairs circadian robustness, and may contribute to the wide‐ranging phenotypes of CRY‐deficient mice. Rather than generating large‐scale daily variation in proteome composition, we suggest it is plausible that the various transcriptional and post‐translational functions of CRY proteins ultimately act to maintain protein and osmotic homeostasis against daily perturbation.

## Graphical abstract

CRYPTOCHROMES are not essential for circadian proteome oscillations but play a crucial role in the temporal regulation of protein and osmotic homeostasis.

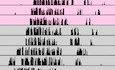

## Introduction

From transcriptional activation and RNA processing, to protein synthesis, folding and degradation, multiple mechanisms operate at every stage of gene expression to ensure that each cellular protein is maintained in a concentration range appropriate to its biological function (Wolff *et al*, 2014; Harper & Bennett, 2016; Juszkiewicz & Hegde, 2018). Protein homeostasis is essential for cell viability and osmotic homeostasis, with the correct temporal regulation of protein activity being critical to every biological process—too much or too little at the wrong time underlies most pathological states (Labbadia & Morimoto, 2015; Balchin *et al*, 2016).

In mammals, cellular physiology is temporally orchestrated around daily cycles that regulate most biological functions to a circadian rhythm, whereas circadian dysregulation is strongly associated with disease states (Cederroth *et al*, 2019). On any given day, the circadian cycle is expressed by more cells of the human body than the cell division cycle, with daily rhythms in clock‐controlled protein abundance thought to be the fundamental basis by which cell biology anticipates and accommodates the predictable demands of day and night (Dunlap, 1999; Wong & O’Neill, 2018; Cederroth *et al*, 2019; Cox & Takahashi, 2019). Individual cellular rhythms are synchronised by endocrine cues such as insulin and glucocorticoid signalling, which function *in vivo* to align internal cellular timing with environmental cycles (Crosby *et al*, 2019). Daily rhythms in proteome composition have been observed *in vivo* and persist in cultured cells under constant conditions *ex vivo* (Reddy *et al*, 2006; Hoyle *et al*, 2017). Typically, the proportion and relative amplitude of rhythmically abundant proteins across a range of cellular contexts is ˜10–20% (Reddy *et al*, 2006; Mauvoisin *et al*, 2014; Robles *et al*, 2014; Hoyle *et al*, 2017). There is little direct evidence that such modest variation would necessarily elicit rhythms in protein function, however, given that protein abundance is rarely rate limiting for protein activity under physiological conditions (Aragón & Sols, 1991; Nadaraia *et al*, 2007; Rocca *et al*, 2015; Bulik *et al*, 2016; Stangherlin *et al*, 2021a).

Circadian regulation of a protein’s abundance is most frequently attributed to cycling transcription of the encoding gene, despite recent investigations having revealed that mRNA and protein abundances correlate quite poorly and that post‐transcriptional and post‐translational regulatory processes are at least as important (Ukai & Ueda, 2010; Liu *et al*, 2016a; Takahashi, 2016; Franks *et al*, 2017; Wang *et al*, 2018). Global transcriptional oscillations are facilitated by daily cycles of transcriptional repression and derepression effected by products of the *Period1/2* and *Cryptochrome1/2* genes, and fine‐tuned by various auxiliary but non‐essential transcriptional feedback mechanisms (Ukai & Ueda, 2010; Takahashi, 2016). The transcription of *Period*, *Cryptochrome* and other genes is stimulated by complexes containing the activating transcription factor BMAL1. The stability, interactions and activity of the encoded PER and CRY proteins are regulated post‐translationally until, many hours later, they repress the activity of BMAL1‐containing complexes in a transcriptional‐translation feedback loop (TTFL).

Within the TTFL circuit, CRY proteins are the essential repressors of BMAL1 complex activity (Ye *et al*, 2014; Chiou *et al*, 2016), whereas PER proteins are thought to play critical signalling and scaffolding roles, required for the nuclear import and targeting of CRY to BMAL1‐containing complexes (Chiou *et al*, 2016). CRY proteins also function as adaptors for the recruitment of E3 ubiquitin ligase complexes that target many proteins, including transcription factors, for ubiquitin‐mediated proteolysis (Correia *et al*, 2019). According to the current widely accepted paradigm therefore, CRY‐mediated transcriptional feedback repression is indispensable for the cell‐autonomous circadian regulation of clock‐controlled protein abundance (van der Horst *et al*, 1999; Kume *et al*, 1999; Sato *et al*, 2006; Ukai‐Tadenuma *et al*, 2011; Ode *et al*, 2017), and this is proposed to drive the daily co‐ordination of cellular activity rhythms (Dunlap, 1999; Cederroth *et al*, 2019; Cox & Takahashi, 2019).

Critically though, CRY‐deficient cells and tissues remain competent to sustain circadian timing in the absence of canonical TTFL function (Maywood *et al*, 2011; Ono *et al*, 2013a; Putker *et al*, 2021), with a mechanism that is dependent on casein kinase 1 and protein degradation, as in wild‐type controls, as well as in naturally anucleate red blood cells (Cho *et al*, 2014; Henslee *et al*, 2017; Beale *et al*, 2019). Similarly, circadian oscillations persist in cells lacking BMAL1 (Lipton *et al*, 2015). Since BMAL1 and CRY proteins are required for normal circadian regulation *in vivo* and also required for TTFL function, it was thought that the complex phenotype of BMAL1‐ or CRY‐deficient mice arises because circadian transcriptional cycles are crucial for cellular and organismal physiology more generally (Maury *et al*, 2010; Yu & Weaver, 2011). The emerging observation that BMAL1 and CRY‐deficient cells and tissues are competent to sustain certain elements of circadian regulation challenges this interpretation and suggests an alternative: that some or all phenotypic complexity associated with deletion of a “clock gene” arises from additional functions of the encoded protein beyond the canonical TTFL model. Supporting this, recent reports imply that various pathophysiological consequences of CRY deficiency might be directly attributable to the absence of CRY function rather than impairment of circadian transcriptional regulation (Mauvoisin *et al*, 2014, 2017; Papp *et al*, 2015; Jordan *et al*, 2017; Kriebs *et al*, 2017). Overall then, current evidence broadly supports the hypothesis that CRY‐mediated circadian transcriptional feedback repression is crucial for cycling TTFL activity, associated with rhythmic robustness and clock‐controlled protein rhythms (Sato *et al*, 2006; Ukai‐Tadenuma *et al*, 2011; Chiou *et al*, 2016; Henslee *et al*, 2017; Ode *et al*, 2017; Wong & O’Neill, 2018), but is not essential for the cell‐intrinsic capacity to maintain daily timekeeping (Putker *et al*, 2021).

To gain insight into how CRY confers rhythmic robustness, and directly test the hypothesis that CRY is essential for circadian regulation of proteome composition, we investigated the molecular consequences of CRY deficiency on cellular protein expression over several days using an unbiased whole‐cell (phospho)proteomic strategy. Our findings reveal that CRY is not essential for circadian regulation of steady‐state (phospho)protein abundance or compensatory rhythms in ion transport, as well as suggesting a crucial role for CRY in the maintenance of protein and osmotic homeostasis. CRY‐deficient cells appear to be in a state of mild chronic stress that may underlie their reduced robustness as well as the complex and numerous consequences of CRY deletion in mice and their tissues.

## Results

### Cell‐autonomous proteome and phosphoproteome rhythms in the absence of CRY

**Figure 1:**
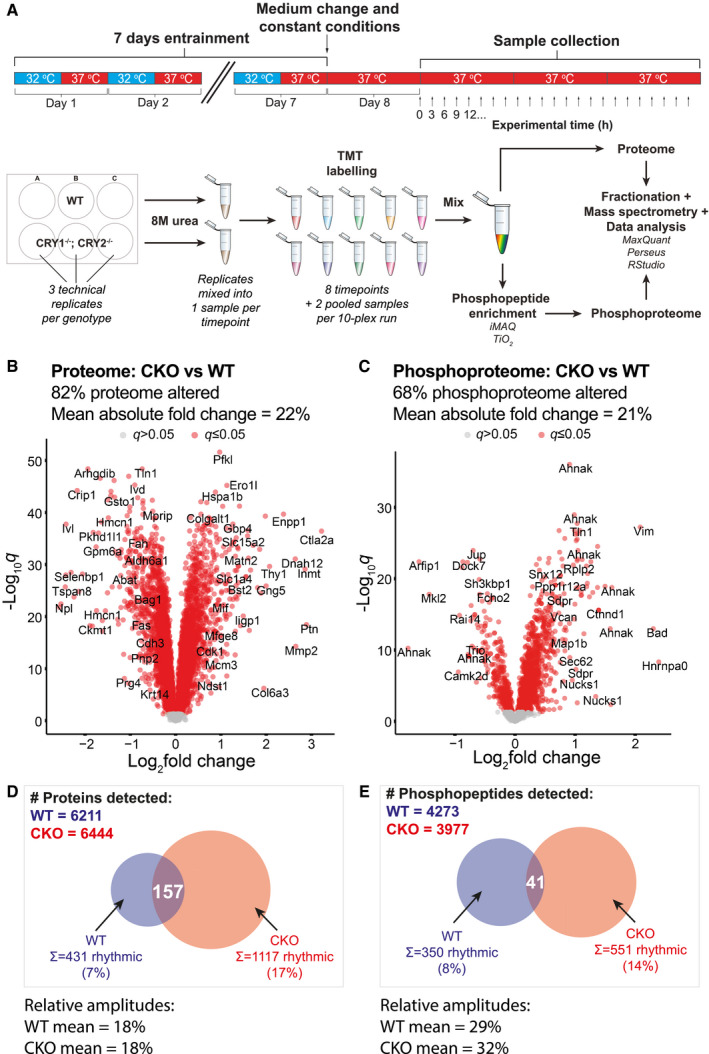
Overall and time‐dependent differences of proteome and phosphoproteome composition between WT and CRY‐deficient cells AAn overview of the proteomics experimental workflow. Samples were taken every 3 h for 3 days in constant conditions, starting 24 h after medium change (“Experimental time 0 h”).B, CVolcano plots showing the fold change in average expression of all proteins and phosphopeptides, respectively, in CKO cells compared with WT (q = Benjamini–Hochberg‐corrected *P*‐value, *n* = 24 time points over 3 days). Statistically significant changes (*q* ≤ 0.05) are shown in red. Some proteins are labelled as space allows.D, EVenn diagrams showing the numbers of rhythmic proteins and phosphopeptides, respectively, in WT cells and CKO cells, with the overlaps annotated. Mean relative amplitude of rhythmic (phospho)peptides is also provided.

To understand the proteomic and phosphoproteomic consequences of CRY deletion, we used confluent primary mouse fibroblasts, a classic model of cellular circadian timekeeping where contact inhibition prevents any interference from the cell division cycle (Hoyle *et al*, 2017). Wild‐type (WT) and CRY1^−/−^; CRY2^−/−^ (CKO) mouse fibroblasts in cell culture were isolated from otherwise isogenic mice and synchronised using daily temperature cycles then sampled in parallel under constant conditions every 3 h over 3 days (Fig 1A). Quantitative proteomics detected over 6,000 proteins and around 4,000 phosphopeptides in both cell lines. We were surprised to find that, averaging across the time course, the overall abundance and relative phosphorylation of most detected proteins were significantly altered in the CKO cells compared with WT controls (Fig 1B and C), by an average of 21%. The striking CRY‐dependent change in overall proteome composition was similar to previous findings in CKO mouse liver (Mauvoisin *et al*, 2014; Appendix Fig S1A).

Over this 3‐day cellular time course, as expected (Sato *et al*, 2006; Ukai‐Tadenuma *et al*, 2011; Koike *et al*, 2012), CRY1 was selectively detected in WT, but not CKO cells, and displayed a ˜24 h rhythmic abundance profile across the time course. The delayed phase of CRY1 relative to a PER2 luciferase reporter (PER2::LUC) recorded from parallel replicate cultures (Appendix Fig S1B) is consistent with other reports (Gabriel *et al*, 2021) and serves to validate the approach. Heat maps visualising the rhythmic proteins are shown in Appendix Fig S1D and E, with examples of rhythmic and arrhythmic (phospho)proteins shown in Appendix Fig S1F and G. Based on estimates of intrinsic noise of gene expression (Elowitz *et al*, 2002; Raser & O’Shea, 2004; Pedraza & Van Oudenaarden, 2005; Sigal *et al*, 2006; Volfson *et al*, 2006), we chose a threshold of 10% relative amplitude to define biological significance for protein abundance oscillations; no such studies were available for protein phosphorylation.

In the WT time course, 7% of detected proteins and 8% of detected phosphopeptides showed biologically significant circadian abundance rhythms, consistent with previous reports (Hoyle *et al*, 2017; Collins *et al*, 2021). Unexpectedly, by the same criteria, 17% of detected proteins and 14% of phosphopeptides were rhythmically abundant in the CKO cell time course (Fig 1D and E). Using an independent statistical tool to test for rhythmicity (eJTK cycle), we found that similarly, more rhythmic species were present in the CKO time course compared with WT, also with little overlap (Appendix Fig S1C).

Beyond the finding that circadian regulation of (phospho)proteome composition was at least as prevalent in these CKO cells as their WT controls, we were particularly surprised that overall differences in proteome composition between the two sets of cultures were substantially greater in extent and magnitude than variation over time in either (Fig 1B and D, Appendix Fig S2).

### CRY may suppress some cell‐autonomous rhythms whilst promoting others

**Figure 2:**
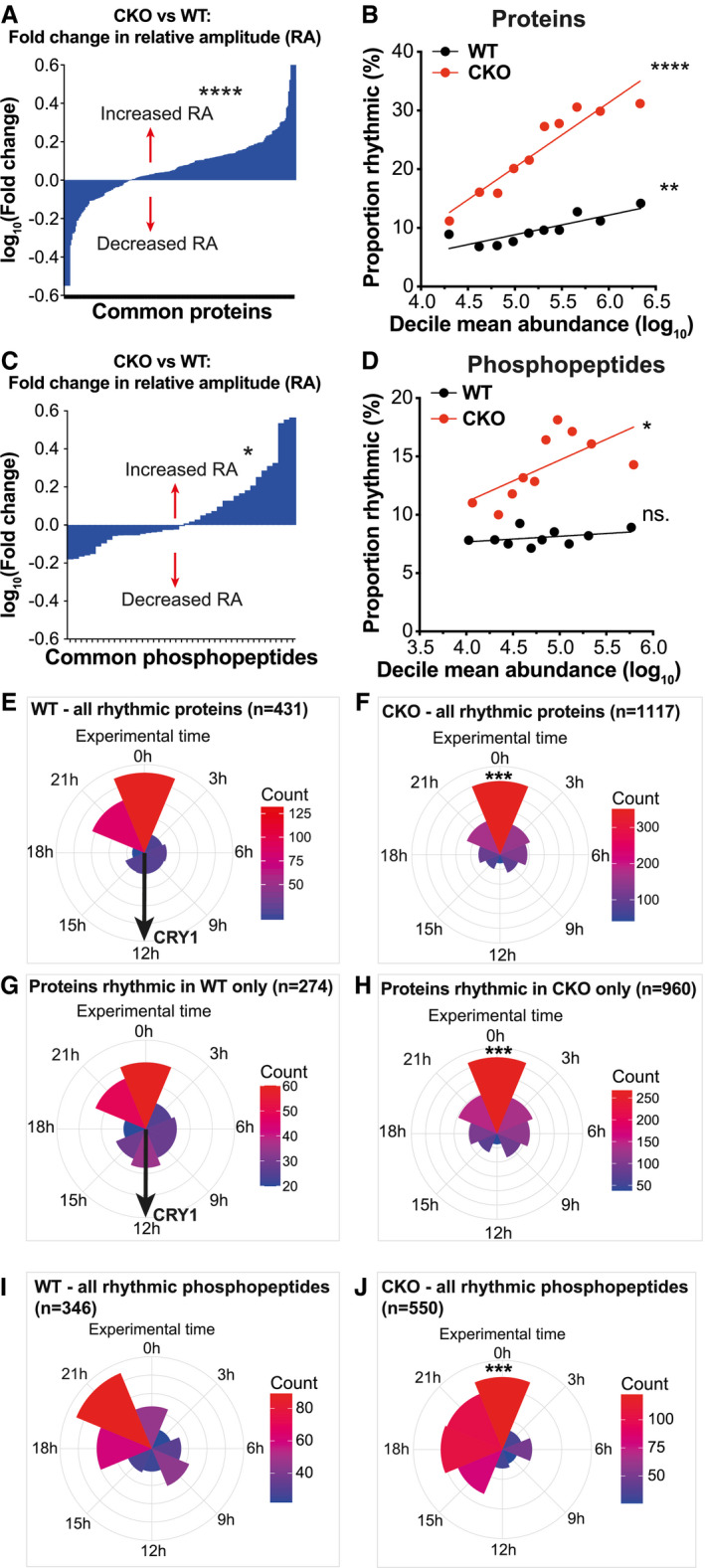
Differences in temporal regulation of the proteome and phosphoproteome between WT and CRY‐deficient cells AFold change in relative amplitude (RA) was calculated for each of the proteins found to be rhythmic in both genotypes by RAIN analysis (i.e. no RA cut‐off). The mean fold change (log) in RA was an increase of 14% in CKO cells compared with WT cells (One sample *t*‐test, ****P < 0.0001).BFor each genotype, all proteins were divided into 10 deciles of equal number, ranked by abundance. The mean abundance of each decile was plotted against the proportion of the decile that was rhythmic. Linear regression lines are shown for each genotype, and the slopes were significantly non‐zero (F‐test, WT **P = 0.0016, CKO ****P < 0.0001). The slopes were also significantly different to each other (*F*‐test, *P* = 0.0001).CFold change in relative amplitude (RA) was calculated for each of the phosphopeptides found to be rhythmic in both genotypes by RAIN. On average, the RA was increased in CKO cells compared with WT cells by 6% (one‐sample *t*‐test, *P < 0.05).DThe same analysis in B) was carried out for phosphopeptides. The slopes were significantly non‐zero in CKO but not WT (*F*‐test, WT *P* = 0.3 (ns.: non‐significant), CKO *P = 0.04). The slopes were also significantly different to each other (*F*‐test, *P* < 0.0001).E–HCircular histograms showing the number of proteins at each rhythmic phase. Phase is defined and estimated by RAIN, as the time of the first predicted peak in a 24‐h period. Concentric circles represent the count scale, with the outermost circle marking the upper end of the counts. Black arrow indicates the phase of CRY1. (E, F) The distributions of rhythmic proteins in CKO cells were significantly different to WT cells (***P < 0.001, Watson’s two‐sample test). (G, H) This was also the case for the proteins rhythmic in only one genotype (***P < 0.001, Watson’s two‐sample test).I, JCircular histograms showing the number of phosphopeptides at each rhythmic phase. The distributions of rhythmic phosphopeptides in CKO cells were significantly different to WT cells (***P < 0.001, Watson’s two‐sample test).

Amongst the proteins that were rhythmically abundant in both the WT and CKO time courses (Fig 1D), there was a modest but significant increase of median relative amplitude in CKO compared with WT cells (Fig 2A). When plotting the mean abundance of each protein (by deciles) against the proportion of proteins within each decile that were detected as rhythmic, we found that more abundant proteins were more likely to be rhythmic than less abundant proteins, with a steeper gradient for the CKO than the WT time course (Fig 2B). Similarly, we found commonly rhythmic phosphopeptides were significantly increased in relative amplitude and abundance in the CKO time course compared with WT (Fig 2C and D). Although, in principle, part of this correlation may be due to preferential and more accurate detection of oscillating high abundance proteins (Laloum & Robinson‐Rechavi, 2020), this explanation cannot account for the apparent differences in relative amplitude and abundance vs. rhythmicity when comparing the two time courses. Given the function of CRY in transcriptional repression and as an E3 ligase adaptor, a parsimonious explanation could be that normally CRY functions to damp circadian rhythms in the abundance of some cellular (phospho)proteins, rather than generate them.

Within both time courses, we noted the phase distribution of rhythmic proteins was clustered around experimental time 0 h (Fig 2E and F), when PER2::LUC activity in wild‐type cells was maximal (Appendix Fig S1B). This was true for most proteins and phosphopeptides that were only rhythmic in either the WT or CKO time courses (Fig 2G and H). However, a subset of rhythmic proteins (including CRY1) were clustered around experimental time 12 h in the WT time course only, and noticeably absent from the CKO time course (Fig 2H). In WT cells, clustering of protein phosphorylation occurred at an earlier phase (by 3 h) compared to rhythmically abundant proteins and PER2::LUC (Fig 2I), with higher relative amplitude (Fig 1E). This phase relationship has strong similarities with observations in mouse liver *in vivo* (Robles *et al*, 2016). Whilst the same was true for the CKO time course, there was a much broader distribution of phase among rhythmic phosphopeptides across 9 h that preceded peak rhythmic protein abundance (Fig 2J).

These observations suggest that cell‐autonomous rhythmic regulation of protein abundance and phosphorylation can occur independently of CRY. It is important to note that a single biological replicate was used and thus, from these data alone, no firm conclusions about any specific genotype‐dependent result can be drawn. We do note, however, that the broad differences in steady state and rhythmic protein/phosphopeptide abundance between the two sets of cultures might suggest that the synthesis and degradation of many proteins, as well as the phosphorylation and dephosphorylation of many phosphosites, are coupled through a mechanism that requires CRY to be present. A more detailed analysis of these data and their possible interpretation can be found in Appendix Figs S2–S4.

### Validation of key proteomic findings

The overall differences in proteome composition between the WT and CKO time courses were greater than change in composition over time in either genotype (Fig 1B and D, Appendix Fig S2B). This would suggest CRY‐deficient cells are effectively in a state of proteome imbalance with respect to WT cells (Harper & Bennett, 2016). Moreover, the divergent identities of rhythmically abundant proteins indicate that CRY proteins make an important contribution to the temporal and overall regulation of proteome composition when they are present. However, an alternative possibility was that any specific differences highlighted by comparisons between the 3‐day WT and CKO proteomic time courses (Figs 1 and 2) arose from founder effects during fibroblast isolation and subsequent expansion (Smith & Whitney, 1980; Phinney, 2012) and therefore not directly attributable to CRY deficiency. To distinguish between these two possibilities, we sought to validate the two major findings of our circadian proteomics study: that genotype‐dependent differences in proteome composition are significantly greater than variation over time within each genotype and that the majority of proteins that change over time within each genotype are different between WT and CKO cells.

**Figure 3:**
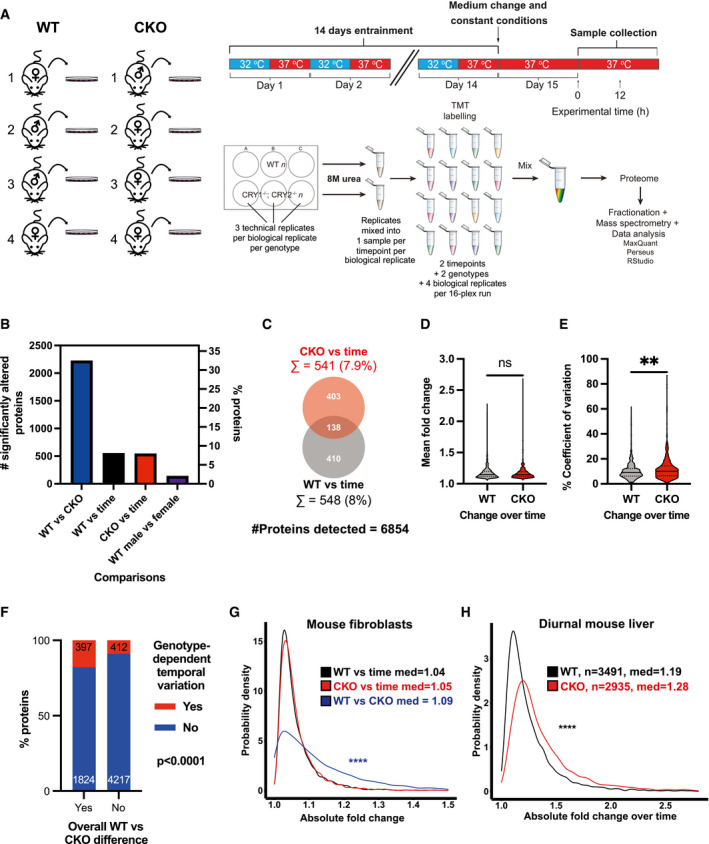
Validation of overall and time‐dependent differences of proteome composition between WT and CKO cells ASchematic of proteomics experimental pipeline. Lung fibroblasts from 8 mice (4 WT, 4 CKO) were isolated and expanded. Samples were taken 12 h apart, starting 24 h after a medium change (“Experimental time 0 h”).BBar chart reports the number and % of detected proteins showing a significant difference in abundance for the listed comparisons by two‐way ANOVA correcting for multiple comparisons, controlling for false discovery rate by the Benjamini, Krieger and Yekutieli method (*q* = 0.05).CMost of the proteins whose abundance changed significantly over time differed between WT and CKO cells.D, EProteins with significant abundance change over time had no significant difference in mean absolute fold change between WT and CKO cells (D, *P* = 0.47), but significantly greater variance in absolute fold change between independent CKO biological replicates than between their WT counterparts (E, **P = 0.0013); Kolmogorov–Smirnov test (*n* = 4). Continuous lines = median, dotted lines = quartiles.FProteins with significant differences in overall abundance between CKO and WT cells were more likely to show genotype‐dependent differences in temporal variation (Fisher’s exact test, *P* < 0.0001), number of proteins in each group reported.GAbsolute fold changes were calculated from maximum/minimum for all protein abundances and probability densities plotted. There were significant differences between WT vs. time fold changes and WT vs. CKO fold changes (****P < 0.0001). There was no significant difference between WT vs. time fold changes and CKO vs. time fold changes (*P* = 0.930), Kruskal–Wallis test with Dunnett’s multiple comparisons test, *P*‐values adjusted with Benjamini–Hochberg method.HData were extracted from Mauvoisin *et al* (2014), where the proteome was quantified in WT and CKO mouse livers extracted under diurnal conditions. Absolute fold change was calculated from maximum/minimum protein abundances. Probability density distributions for fold change are plotted. Kolmogorov–Smirnov test: ****P < 0.0001.

To do this, we employed 16‐plex TMT quantitative mass spectrometry to compare temporal variation between two sets of four independently generated primary WT and CKO fibroblasts, isolated from 8 different male and female mice (Fig 3A). Cells were plated, synchronised and sampled under identical conditions to the first time course, at the two circadian phases where our earlier analyses predicted the greatest variation in proteome composition, after 24 h and 36 h under constant conditions (experimental time 0 h and 12 h, Fig 3A). The use of cells from male and female WT mice allowed us to estimate variation due to stochastic (genotype‐independent) differences between cell lines. We predicted a large difference in proteome composition between CKO and WT cells and modest variation over time within each genotype, whereas we had no reason to expect any substantial sex‐dependent difference in proteome composition (Wu *et al*, 2020).

The results were consistent with our prediction (Fig 3B). In both genotypes, the number of detected proteins with significant temporal variation was ˜4‐fold greater than that whose abundance varied significantly with sex (modelling founder effects arising and fixed during cell isolation/expansion), with a similar proportion (8%) of WT proteins showing significant temporal variation to the proportion that were rhythmically abundant in the 3‐day time course. This supports there being *bona fide* differences in the abundance of many proteins at opposite phases of the circadian cycle. Recapitulating our earlier observation, there was also poor similarity in the identity of proteins whose abundance significantly changed over time between the two genotypes (Fig 3C–E). Also, as in the 3‐day time course (Appendix Fig S2A), we found that significant change in a protein’s overall abundance between WT and CKO cells was strongly associated with genotype‐dependent differences in abundance change over time (Fig 3F). Most critically, the number of proteins with significant genotype‐dependent differences in overall abundance was ˜4‐fold greater than the number with time‐dependent variation within each genotype (Fig 3B), validating our previous finding (Fig 1B and D). This further supports there being a consistent difference in overall proteome composition between WT and CKO fibroblasts which is substantially greater than any temporal variation (Fig 3G), and with little identity shared between those proteins whose abundance changes over time within each genotype (Fig 3C).

Similar to the 3‐day time course, which analysed a single biological replicate with high temporal resolution, analysis of 4 independent biological replicates per genotype at two time points revealed minimal difference in the extent of temporal variation between genotypes (compare Appendix Fig S2B with Fig 3G). Of those proteins with significant temporal variation, we observed similar magnitude of change but significantly greater variance in the extent of change over time in CKO compared with WT cells (Fig 3D and E). This indicates that temporal variation in protein abundance was greater between different CKO cells than between different WT cells, consistent with our observations from the first time course, where more proteins were detected as rhythmic in CKO than WT cells. Taken together, this suggests that CRY normally contributes to the temporal maintenance of proteome composition.

Following this line of argument, if CRY functions to maintain proteome composition over time in isolated cells, then any differences between WT and CKO proteomes would be amplified *in vivo*, where tissues are continually exposed to daily external stimuli, e.g. cycling hormonal cues, temperature fluctuations, feeding/locomotor activity rhythms. We tested this by calculating the daily fold change in protein abundance detected by Mauvoisin *et al* (2014) in WT and CKO mouse liver samples collected at 4 matched time points over a diurnal cycle. We found significantly greater daily variation of protein abundance in CKO compared with WT tissue (Fig 3H), with time‐dependent differences that were much greater than observed in isolated cells. Therefore, in mouse liver *in vivo*, CRY apparently functions to maintain protein homeostasis by suppressing temporal variation in proteome composition.

### CRYPTOCHROME regulates proteasome activity and translation rate

**Figure 4:**
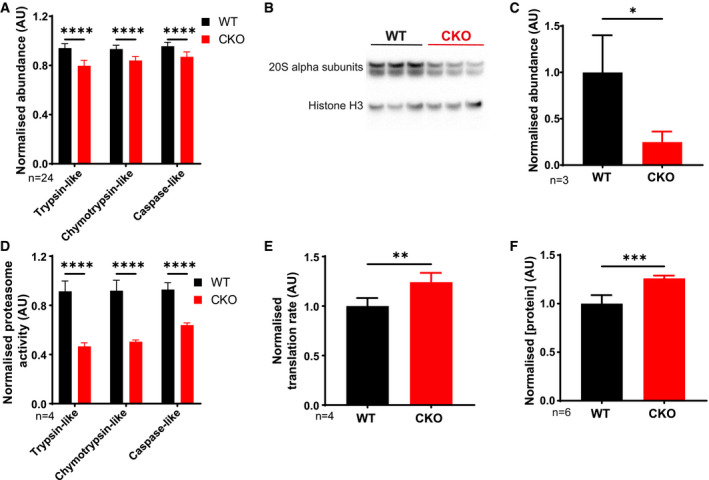
Differences in proteasome activity and translation rate between WT and CRY‐deficient cells From the quantitative proteomics experiment, average abundance of catalytic proteasome subunits was calculated and normalised to WT means. Trypsin‐like (β2), chymotrypsin‐like (β3) and caspase‐like (β1) catalytic subunits are shown. The average was calculated from all 24 time points of the proteomics experiment. Mean ± SD, 2‐way ANOVA with Holm–Sidak’s multiple comparisons, ****P ≤ 0.0001.Representative Western blot using an antibody that recognises all 7 α subunits of the 20S proteasome, with anti‐histone H3 as loading control.Quantification of the blots in (B), using all replicates (*n* = 3), normalised to WT mean. Mean ± SD, one‐tailed Student’s *t*‐test with Welch correction‐prior prediction from experiment in (A) that CKO abundance would be lower, *P ≤ 0.05.Proteasome activity measured using the ProteasomeGlo Assay (Promega), normalised to WT means. Mean ± SD, 2‐way ANOVA with Holm–Sidak’s multiple comparisons. *N* = 6 experiments, representative experiment shown (*n* = 4 technical replicates), ****P ≤ 0.0001.Translation rate was measured using ^35^S‐methionine labelling and imaging with phosphor screens. The quantification values were normalised to the total protein concentration as measured using Coomassie stain and then normalised to WT mean. Mean ± SD, Student’s *t*‐test with Welch correction. *N* = 3 experiments, representative experiment shown (*n* = 4 technical replicates), **P ≤ 0.01.Total protein mass per cell in confluent WT and CKO cultures. Cells were grown in two 12‐well plates; one was used for cell counting and the other was used for lysis in RIPA buffer prior to protein quantification by BCA assay. Quantification shown, normalised to WT mean. Mean ± SD, Student’s *t*‐test with Welch correction, *n* = 6 technical replicates.

We next explored potential mechanisms for the widespread proteome changes in CKO cells by searching our dataset for key factors regulating protein synthesis and degradation. We did not interrogate the transcriptome of CKO cells, since there is now an overwhelming consensus that variation in cellular mRNA abundance is poorly predictive of variation in protein abundance (Reddy *et al*, 2006; Deery *et al*, 2009; Schwanhäusser *et al*, 2011; Vogel & Marcotte, 2012; Mauvoisin *et al*, 2014; Robles *et al*, 2014; Stitt & Gibon, 2014; Liu *et al*, 2016b; Fortelny *et al*, 2017; Hurley *et al*, 2018; Rey *et al*, 2018; Buccitelli & Selbach, 2020; preprint: Brunner *et al*, 2021). We found a striking reduction in the abundance of catalytic proteasomal subunits (Fig 4A, Appendix Fig S5A) which we validated by Western blot and enzymatic assays of proteasome activity (Fig 4B–D, Appendix Fig S5B).

Whilst there was no consistent change in ribosomal subunit abundance, we also observed a significant increase in cytosolic protein synthesis rate by ^35^S‐methionine incorporation (Fig 4E, Appendix Fig S5C and D), as observed previously by puromycin incorporation (Putker *et al*, 2021). We considered that the combined effect of reduced proteasomal activity and increased translation rate would affect the overall steady‐state levels of cellular protein. We found this to be the case, with a modest but significant increase in overall protein per CKO cell compared with WT (Fig 4F).

These cultured CKO cells express more protein than WT cells, with increased translation and decreased proteasomal degradation. Altogether, and in the light of previous observations (Morimoto & Cuervo, 2009; Klaips *et al*, 2018; O’Neill *et al*, 2020), our data suggest that CKO cells likely maintain a different set point for protein homeostasis, as also occurs in many (pre‐)pathological states (Labbadia & Morimoto, 2015; Balchin *et al*, 2016).

### Rhythmic regulation of ion transport and protein content is CRY‐independent

Mammalian and other eukaryotic cells devote significant resources to ensuring osmotic equilibrium over the plasma membrane, in order to maintain cell volume and prevent protein aggregation (Choe & Strange, 2008; Baumgarten & Feher, 2012; Lipton *et al*, 2017; O’Neill *et al*, 2020). Changes in cytosolic macromolecule content are balanced by compensatory ion transport, primarily mediated by K^+^, the major cellular osmolyte (O’Neill *et al*, 2020; Stangherlin *et al*, 2021b). Two independent lines of evidence suggested that CKO cells would exhibit altered osmotic homeostasis.

**Figure 5:**
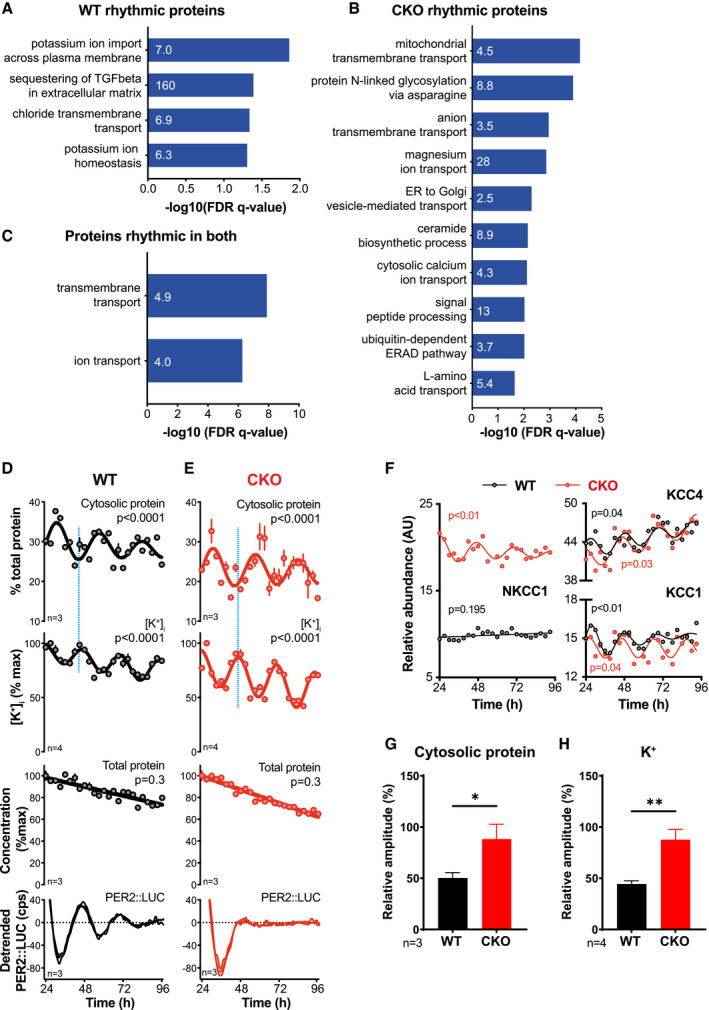
Time‐dependent differences in cellular composition and ion transport between WT and CRY‐deficient cells A–CGene ontology analysis for rhythmic proteins was carried out using Gene ontology enrichment analysis and visualisation (GOrilla) (Eden *et al*, 2007, 2009). Significantly rhythmic WT proteins were compared against background (all proteins identified in the experiment), and the top non‐overlapping GO Biological Process terms shown, sorted according to FDR q‐value. Fold enrichment is annotated on each bar (A). The same GO analysis was carried out comparing proteins rhythmic that were rhythmic in CKO cells (B) and for proteins that were rhythmic across both genotypes (C).D, EFrom one time course experiment, ions, cytosolic proteins and total protein were extracted in parallel samples. The presented experiment is representative of 3 separate time course experiments that were carried out (*N* = 3). Representative experiment shown (*n* = 4 technical replicates for [K^+^], *n* = 3 technical replicates for the other traces). Blue lines highlight the antiphasic relationship between oscillations in cytosolic protein and potassium concentration. Mean ± SEM, *P*‐values from RAIN, red lines are fits by a damped cosine compared with a straight line (null hypothesis). Parallel PER2::LUC recordings were also performed and plotted as a phase marker.FExamples of key ion transporters are shown, as detected in the proteomics experiment. *P* values show the results of an *F*‐test comparing fits of damped cosine against straight line. All proteins except WT NKCC1 had RAIN *P* < 0.05.G, HRelative amplitudes of cytosolic protein (G) and potassium concentrations oscillations (H) in (D) and (E) were greater in CKO compared to WT (Student’s *t*‐test with Welch correction, mean ± SD, *P ≤ 0.05, **P ≤ 0.01).

First, we searched for rhythmically regulated cellular processes in CKO and WT cells using gene ontology (GO) analysis. Of the rhythmically abundant proteins, ranked GO analysis revealed consistent enrichment for processes associated with ion transport, both in wild‐type in and CKO cells when analysed separately or combined (Fig 5A–C). Interrogation of the proteomics dataset then revealed altered expression levels and increased rhythmic amplitudes of many ion transporters in CKO compared with WT cells (Appendix Fig S6A and B). This included several members of the SLC12A family of electroneutral transporters (Fig 5F) that contribute to the maintenance of osmotic homeostasis against variations in cytosolic protein concentration in wild‐type cells over the circadian cycle (Stangherlin *et al*, 2021b). Second, since many proteins are cytoplasmic, we considered that if CRY normally suppresses rhythms in the abundance of many individual proteins that normally peak around the same circadian phase (Fig 2E–H), then the consequence of CRY deletion would be to increase the overall amplitude of daily rhythms in soluble cytosolic protein content.

To validate this, we measured the K^+^ and cytosolic protein content of cells across the circadian cycle. Consistent with previous investigations (Stangherlin *et al*, 2021b), in WT cells, K^+^ and digitonin‐extracted cytosolic protein concentrations exhibited antiphasic circadian rhythms (Fig 5D and Fig S6C), with no significant daily variation in total cellular protein. The same was observed in CKO cells (Fig 5E), but with higher relative amplitudes for soluble protein and K^+^ (Fig 5G and H). Considering previous observations (Stangherlin *et al*, 2021b), the higher amplitude cytosolic protein rhythm in CKO cells likely drives the higher amplitude K^+^ rhythms. This is likely facilitated by increased expression and amplitude of SLC12A transporter activity (Fig 5F), which buffers cellular osmotic potential in response to greater changes in cytosolic macromolecular content over the circadian cycle (Stangherlin *et al*, 2021b).

### CRY‐deficient cells are more sensitive to proteotoxic stress

The viability of CKO cells and mice clearly suggests that they do maintain protein homeostasis overall, despite proteome imbalance and an altered set point for protein homeostasis compared with WT controls. Proteome imbalance typically renders cells more sensitive to stress (Harper & Bennett, 2016). Using ranked GO analysis of overall protein fold changes compared with WT, we found that the expression of proteins involved in “response to stress” was increased in CKO compared with WT cells (Appendix Fig S7A), suggesting that the proteostasis network in CKO cells is in an activated state (Kroemer *et al*, 2010; Pakos‐Zebrucka *et al*, 2016; Klaips *et al*, 2018).

**Figure 6:**
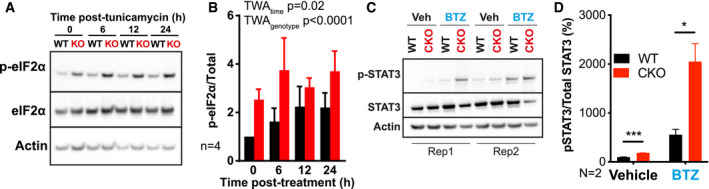
CRY‐deficient cells and tissues are more sensitive to stress WT and CKO cells were treated with 500 nM tunicamycin (TUN) and lysed in RIPA buffer at time points between 0 and 24 h afterwards. Western blots were carried out, probing for phosphorylated eIF2α, total eIF2α and actin. Representative blots shown, *n* = 4.Quantification of all replicates represented in (A), where phosphorylated eIF2α is normalised to total eIF2α. Mean ± SD. 2‐way ANOVA (TWA) with Holm‐Sidak’s multiple comparisons.Mice were housed in 12‐h:12‐h light–dark cycles, with unrestricted feeding. Mouse lungs were collected 7 h after transition to the light phase, 5 h after intraperitoneal injection of mice with bortezomib (BTZ, 2.5 mg/kg) or vehicle (Veh, 1% DMSO in sterile PBS). Tissues were lysed in RIPA buffer and Western blots were performed, probing for phosphorylated STAT3, total STAT3 and actin. Male (left) and female (right) replicates shown, *N* = 2 mice for each condition.Quantification of the blots shown in (C), where phosphorylated STAT3 is normalised to total STAT3. Mean ± SD. Multiple *t*‐tests, corrected for multiple comparisons with the Holm–Sidak method, *P ≤ 0.05, ***P ≤ 0.001. Source data are available online for this figure.

We therefore hypothesised that CKO cells may be more susceptible to proteotoxic stress. To test this, we probed WT and CKO cells for phosphorylation of eIF2α, a well‐characterised marker of the integrated stress response (ISR). As a control, we treated cells with tunicamycin, which gradually induces the integrated stress response *via* inhibition of secretory pathway protein glycosylation (Heifetz *et al*, 1979; Oslowski & Urano, 2011). We observed increased eIF2α phosphorylation in CKO compared with WT cells, at all time points (Figure 6A and B), indicating increased proteotoxic stress in these CKO cells.

We next sought to test whether increased stress was associated with CRY deficiency in mouse tissues. STAT3 is a transcription factor whose phosphorylation on Tyr705 is a well‐established inflammatory marker for both chronic and acute cellular stress *in vivo,* across a range of cell types (Yu *et al*, 2009; Hu *et al*, 2014; Xu *et al*, 2020). We therefore probed for phosphorylated STAT3 in mouse lungs 5 h after intraperitoneal injection of bortezomib (BTZ, proteasome inhibitor) or vehicle (control). Compared with WT tissue, we observed increased STAT3 phosphorylation in control CKO mouse lungs, which was further elevated above WT upon BTZ treatment (Figure 6C and D). This shows CRY deficiency is associated with increased stress and increased sensitivity to stress in mouse tissue, as well as cultured primary cells.

### Proteotoxic stress impairs rhythmic robustness

Daily rhythms of PER2 activity in CKO cells (as measured by PER2::LUC bioluminescence) are less robust than in WT cells, being more variable in their expression and damping more rapidly (Putker *et al*, 2021). At the outset of this investigation, we asked how CRY confers increased robustness upon cellular circadian rhythms observed in WT cells, and found evidence suggesting that proteome imbalance in CRY‐deficient cells renders them more sensitive to stress. Noting that several different cellular stressors (reductive, oxidative, metabolic, transcriptional inhibition) have previously been reported to reversibly attenuate the expression of cellular circadian rhythms (Dibner *et al*, 2009; Putker *et al*, 2017), our observations suggested the hypothesis that the altered set point of protein homeostasis in CKO renders them more susceptible to stress, which in turn leads to their reduced rhythmic robustness. This informed the prediction that circadian rhythms in WT cells would become less robust, and therefore damp more rapidly, when subject to chronic proteotoxic stress.

**Figure 7:**
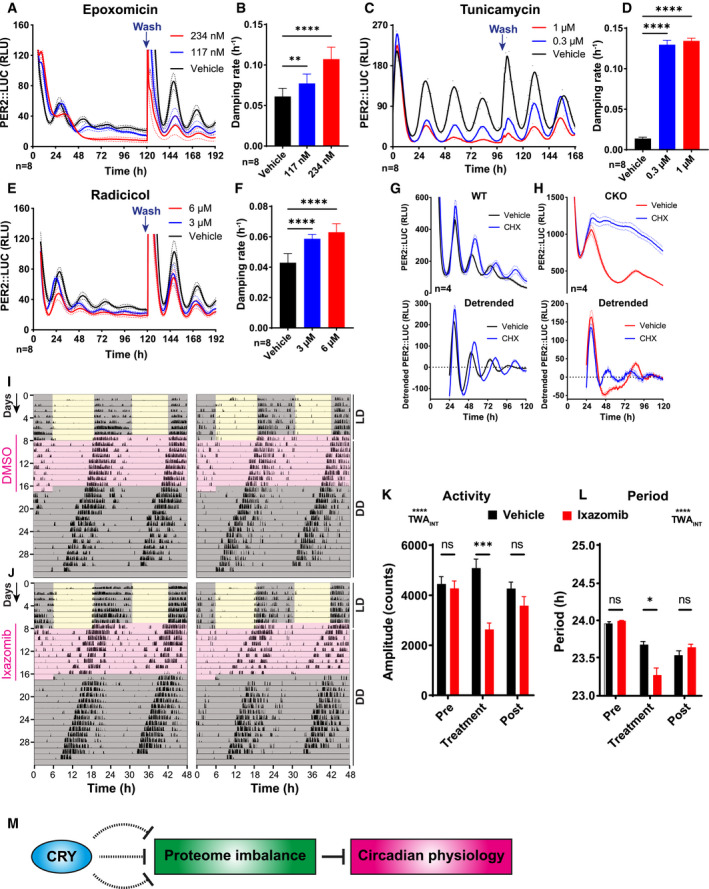
WT cells recapitulate the CRY‐deficient phenotypes upon induced chronic stress AWT PER2::LUC cells were treated with epoxomicin or vehicle control. Blue arrows show the time points where drug was washed off. Mean (solid lines) ± SD (dotted lines).BQuantification of damping rate of the PER2::LUC recordings shown in (A), for the duration of drug treatment. Mean ± SD. One‐way ANOVA with Holm–Sidak’s multiple comparisons, **P ≤ 0.01, ****P ≤ 0.0001.CWT PER2::LUC cells were treated with tunicamycin or vehicle control. Mean (solid lines) ± SD (dotted lines).DQuantification of damping rate of the PER2::LUC recordings shown in (C), for the duration of drug treatment. Mean ± SD. One‐way ANOVA with Holm–Sidak’s multiple comparisons, ****P ≤ 0.0001.EWT PER2::LUC cells were treated with radicicol or vehicle control. Blue arrows show the time points where drug was washed off. Mean (solid lines) ± SD (dotted lines).FQuantification of damping rate of the PER2::LUC recordings shown in (E), for the duration of drug treatment. Mean ± SD. One‐way ANOVA with Holm–Sidak’s multiple comparisons, ****P ≤ 0.0001.G, HWT and CKO cells were treated with 0.3 µM cycloheximide (CHX) or DMSO vehicle control, and kept in constant conditions, *n* = 4, Mean ± SD. Panels above show baseline‐subtracted raw data, and panels below show data detrended with a 24‐h moving average.I, JRepresentative double‐plotted actograms of WT mice receiving drinking water with DMSO vehicle control (*N* = 7 mice) or 35 µg/ml ixazomib (*N* = 8 mice). Two representative actograms are shown per condition. For the first 7 days, the mice were in 12:12‐h light–dark cycles (LD), and had drinking water supplemented with blackcurrant squash. Ixazomib/DMSO was added to this, and the lighting was changed to constant darkness (DD). After 7 days, the drug/vehicle was removed, and the mice remained in DD until the end of the experiment.K, LQuantification of the amplitude of wheel‐running activity and period length from the experiment shown in (I), (J), including pre‐treatment, during treatment and post‐drug treatment. Mean ± SEM. 2‐way ANOVA with Holm‐Sidak’s multiple comparisons, *P ≤ 0.05, ***P ≤ 0.001, ****P ≤ 0.0001, TWA_INT_ = two‐way ANOVA interaction.MSchematic diagram: our data suggest that CRY deficiency is associated with proteome imbalance, which in turn disrupts circadian regulation of physiology.

To test this, we monitored PER2::LUC rhythms in WT cells where proteotoxic stress was elicited by sustained inhibition of three different pathways: with epoxomicin (proteasome inhibitor), tunicamycin (unfolded protein ER stress response) or radicicol (HSP90 inhibitor). We observed significantly increased damping rate in treated cells compared with controls, which was reversible upon drug removal by a media change (Fig 7A–F). Conversely, the relief of proteotoxic stress, by partial inhibition of protein synthesis with sub‐saturating cycloheximide (CHX) (Kim & Strange, 2013; Parzych *et al*, 2015; Peng *et al*, 2015), was sufficient to partially rescue PER2::LUC rhythms in CKO cells (Fig 7G and H). Critically, the relative amplitude of CHX‐rescued PER2::LUC rhythms was much lower than wild‐type PER2::LUC rhythms, commensurate with the established contribution of CRY proteins in the daily repression of *Period* gene expression, that is absent from CKO cells. This is consistent with our hypothesis (Putker *et al*, 2021) that CRY‐mediated transcriptional feedback amplifies a post‐translational circadian timing mechanism that regulates PER protein stability/activity, that is rendered less robust and frequently masked by proteotoxic stress associated with CRY deficiency.

Extending this hypothesis *in vivo* predicts that cellular stress normally obscures the expression of circadian physiology and behaviour in mouse models. When rhythms are observed in CKO mice (Maywood *et al*, 2011; Ono *et al*, 2013b; Putker *et al*, 2021), after strong environmental cues, the period of oscillation in constant darkness is shorter and the amplitude lower than wild‐type controls. We therefore tested the prediction that the mild continuous proteotoxic stress presented by a continuous low dose of ixazomib (an orally available proteasome inhibitor) would reversibly shorten the period of circadian locomotor activity rhythms and reduce their amplitude, i.e. pushing the phenotype closer to CKO mice. We found that amplitude was reduced, and period length was shortened during 7 days of drug treatment of WT mice compared with vehicle controls, and reversible upon drug removal (Fig 7I–L). Therefore, chronic proteotoxic stress is sufficient to impair robustness of circadian rhythms in cells and mice. In principle, this may contribute to the reduced robustness and timekeeping fidelity of CKO cells as well as diverse phenotypes of CRY‐deficient mice (Fig 7M).

## Discussion

### Cryptochromes are not essential for cell‐autonomous circadian regulation of protein abundance

In this study, we found evidence for circadian regulation of protein abundance in CRY1^−/−^; CRY2^−/−^ (CKO) mouse fibroblasts under constant conditions. This is consistent with previous studies showing circadian oscillations in cellular reporter activity and whole‐organism behaviours in this genetic background (Maywood *et al*, 2011; Ono *et al*, 2013a, 2013b; Putker *et al*, 2021) and is not readily compatible with the prevailing hypothesis that daily cycles of CRY‐mediated transcriptional repression are the essential mechanism that generates circadian rhythms of cellular function and physiology *via* oscillations in the abundance of clock‐controlled proteins (Kume *et al*, 1999; Sato *et al*, 2006; Ye *et al*, 2014). Besides its established interactions with other circadian transcriptional regulators, our observations *in vitro* and *in vivo* are consistent with a model where CRY functions indirectly to promote protein homeostasis, which is important for many cellular functions, including circadian timing. Validating predictions from this model by several approaches, we found that CRY deficiency was linked with proteotoxic stress, which renders circadian rhythms less robust in cells and in mice. There is no reason to doubt that CRY proteins normally regulate circadian transcription, rather our data suggest that the function of CRY‐mediated circadian transcriptional regulation requires urgent reappraisal. In the light of our findings, we propose an experimentally testable refinement to current models for the generation and utility of circadian rhythms in mammalian cells (Appendix Fig S8).

### CRY defends cellular homeostasis, which is permissive for robust circadian rhythms

In order to understand the relationship between proteome imbalance and reduced circadian robustness in CRY‐deficient cells, we used several complementary mass spectrometry techniques. We found the abundance and phosphorylation of very many cellular proteins, as well as the major cellular osmolyte (K^+^), to be profoundly perturbed by CRY deficiency. Indeed, the broad differences in cellular composition between WT and CKO cells (Fig 5, Appendix Fig S6) were much greater than the extent of compositional variation over time, in either genotype; and echo recent observations in NKCC1^−/−^‐deficient cells (Stangherlin *et al*, 2021b). The remodelling of CKO cellular proteomes was associated with increased translation, reduced proteasome activity (Fig 4) and phosphatase abundance (Appendix Fig S4) that would be expected to alter the relative rates of protein synthesis/degradation, and phosphorylation/dephosphorylation, respectively. In principle, this would seem sufficient to account for such widespread changes in steady‐state protein and phosphorylation levels, and it is likely such adaptations contribute to the altered osmotic homeostasis and a state of proteome imbalance of CKO compared with WT cells. Proteome imbalance is established as predisposing cells to stress (Harper & Bennett, 2016; Hipp *et al*, 2019; Vecchi *et al*, 2020) and is therefore very likely responsible for the increased proteotoxic stress and sensitivity to stress observed in CKO cells. Increased proteotoxic stress by three different mechanisms was sufficient to impair robustness of WT circadian rhythms, whereas alleviation of proteotoxic stress rescued more robust rhythms in CRY‐deficient cells. Our data therefore suggest that proteome imbalance and associated stress are likely to underlie the impaired timekeeping fidelity and reduced circadian robustness of CKO cells and tissues *ex vivo*.

CRY proteins are well characterised as promiscuous transcriptional repressors (Koike *et al*, 2012; Kriebs *et al*, 2017; Chan *et al*, 2020) as well as being selective E3 ubiquitin ligase adaptors (Huber *et al*, 2016; Correia *et al*, 2019). Considering the diverse phenotypes, pathologies and proteome changes reported for other E3 ligase adaptor and transcriptional repressor knockout mice (Zhou *et al*, 2013; Scheffner & Kumar, 2014; Lombardi *et al*, 2015; Hoffmann & Spengler, 2019; Liu *et al*, 2020), it is not surprising that the deletion of both *Cryptochrome* genes results in a similarly diverse range of phenotypes; especially when considering the very many identified targets and interacting proteins of CRY1 and CRY2 (Koike *et al*, 2012; Huber *et al*, 2016; Kriebs *et al*, 2017; Correia *et al*, 2019; Chan *et al*, 2020).

Proteome imbalance is already very strongly, and in some cases causally, linked to a range of pathological conditions including chronic inflammation, various cancers, metabolic disorders and neurodegeneration (Yalcin & Hotamisligil, 2013; Agyemang *et al*, 2015; Labbadia & Morimoto, 2015; Harper & Bennett, 2016; Hadizadeh Esfahani *et al*, 2018; Hafycz & Naidoo, 2019; Costa‐Mattioli & Walter, 2020; Yerbury *et al*, 2020). It is therefore quite plausible that proteome imbalance underlies the diverse range of phenotypes exhibited by CKO mice, including impaired body growth (Masuki *et al*, 2005; Bur *et al*, 2009), increased susceptibility to multiple cancers (Lee *et al*, 2010; Kettner *et al*, 2016; Mteyrek *et al*, 2017; Chan & Lamia, 2020), chronic inflammation (Narasimamurthy *et al*, 2012; Hand *et al*, 2016; Cao *et al*, 2017) and dysregulated insulin secretion/fat deposition on high fat diets (Barclay *et al*, 2013). Indeed, during this research we observed that not only do CKO mice eat more and gain less weight than their isogenic WT counterparts, but they also succumbed to spontaneous morbidity and mortality with a much higher frequency (Appendix Fig S7B–E).

### CRY may suppress circadian rhythms in protein abundance and osmotic balance

We noticed that the profound alteration of cellular proteome and ionic composition of CKO cells included a complete reorganisation of the subset of proteins and phosphoproteins that exhibit temporal variation in steady‐state abundance. To our surprise, at least as many proteins showed significant temporal variation in CKO as WT cells, but only a minority of these proteins were common to both genotypes (Figs 1 and 3). In the time course with high temporal resolution, we found that the rhythmic proportion of the proteome was actually greater in CKO compared to WT cells (Fig 1D, Appendix Fig S1C). Our analysis of both sets of proteomics experiments suggested that significant genotype‐dependent differences in the overall abundance of a given protein were associated with a change in whether its abundance changed over time or not (Fig 3F, Appendix Fig S2). A parsimonious interpretation of these findings might be that, in addition to stimulating rhythms in some proteins, directly or indirectly, CRY normally functions to suppress rhythms in the abundance of many others. This idea is further supported by *post hoc* analysis of published data (Fig 3H) from mouse liver collected under diurnal cycles and quantified by an independent method (Mauvoisin *et al*, 2014).

We speculate that CRY could act by (indirectly) coupling rhythms of protein synthesis and degradation to maintain a dynamic steady state with rhythmic flux or “turnover”. Were this correct, it would suggest that removing CRY abolishes rhythms in some proteins whilst unmasking the circadian regulation of others, with the majority of such proteins changing in overall abundance due to the new steady‐state equilibrium that results from a change in their average rate of synthesis relative to degradation. If so, then it informs the explicit prediction that, in wild‐type cells, a much greater proportion of proteins are subject to phase‐coherent circadian regulation of synthesis/degradation than is apparent from their steady‐state abundance (Appendix Fig S8B). This may not be surprising when one considers the extensive evidence for post‐transcriptional and post‐translational circadian regulation of protein synthesis and degradation (Castelo‐Szekely & Gatfield, 2020; Crosby & Partch, 2020), especially given circadian rhythms of protein degradation have been observed in the absence of nascent transcription (Cho *et al*, 2014).

We also found that, compared to WT, CKO cells have increased total protein levels and reduced K^+^ levels, as well as higher amplitude rhythms of cytosolic protein and K^+^. Changes in soluble protein concentration require stoichiometrically larger changes in ion concentration to maintain osmotic homeostasis (Baumgarten & Feher, 2012; Stangherlin *et al*, 2021b). We therefore suggest that CKO cells may have an impaired ability to buffer changes in intracellular osmolarity, particularly in response to external stimuli, which may contribute to their increased sensitivity to stress (Hoffmann *et al*, 2009; Danziger & Zeidel, 2015) and attenuated but prolonged response to growth factor stimulation (Crosby *et al*, 2019). Overall, our findings have significant implications for understanding the function of CRY proteins specifically, and the canonical TTFL more generally, as well as opening several important avenues for future investigation.

### The utility of circadian rhythms for cellular proteostasis

It is frequently suggested that the adaptive advantage conferred by cellular circadian clocks is to anticipate the differential demands of day and night, by turning on genes to accommodate the anticipated increase in demand for the activity of the encoded protein (Dunlap, 1999). The extent of daily variation for most “clock‐controlled proteins” is rather low however (relative amplitude < 20%, Fig 1D, Appendix Fig S2B, Fig 3D), and there is little reason to think that such modest changes in protein abundance would impact on protein function in most cases (Aragón & Sols, 1991; Nadaraia *et al*, 2007; Rocca *et al*, 2015; Bulik *et al*, 2016). Protein synthesis is the most energetically expensive process that most cells undertake however, and multiple mechanisms exist to inactivate and sequester proteins that are not required (Wolff *et al*, 2014; Hipp *et al*, 2019). Moreover, recent reports favour the view that changes in cellular transcriptomes function to buffer cellular proteomes, not perturb them (Liu *et al*, 2016a; Franks *et al*, 2017; Wang *et al*, 2019). Rather than synthesise proteins as and when needed, it makes evolutionary sense that cells would expend energy to ensure a constant abundance of most proteins, which could then be mobilised on demand.

However costly though, damaged/misfolded proteins and activated signal transducers do need to be degraded and replaced to avoid deleterious consequences, such as aggregation and sustained pathway activation. We therefore speculate that, rather than rhythm generation, a fundamental advantage conferred on mammalian cells by the daily regulation of CRY activity, within and beyond the canonical TTFL circuit, is the temporal consolidation of proteome renewal, matching synthesis and degradation rates to keep protein concentrations constant and thereby maintain protein homeostasis over time. Indeed, synchronised daily increases in protein synthesis and degradation are likely to be a prerequisite for the efficient assembly of macromolecular protein complexes (Shiber *et al*, 2018). Supporting this, temporal consolidation of proteome renewal has been observed in yeast cells during their metabolic cycle, a biological oscillation that shares many key features with circadian rhythms in mammalian and other eukaryotic cells (O’Neill *et al*, 2020). This hypothesis leads to the direct prediction that macromolecular complex assembly will be less efficient and more energetically expensive in cells and tissues that lack the capacity for daily regulation of gene expression cycles, such as Cry1/2, Per1/2 and Bmal1 knockouts.

### Caveats to our findings

In this investigation, we specifically addressed how the steady‐state circadian cellular proteome adapts to CRY deficiency in order to understand the impairment to cellular timekeeping. For this reason, we did not investigate transcriptional regulation in CKO cells, which we believe has been adequately characterised in excellent previous work (Kume *et al*, 1999; Sato *et al*, 2006; Vollmers *et al*, 2009; Lamia *et al*, 2011; Ukai‐Tadenuma *et al*, 2011; Jouffe *et al*, 2013; Nangle *et al*, 2014; Putker *et al*, 2021). Moreover, the generally poor correlation between changes in whole‐cell transcriptome with changes in protein abundance and activity (Ohtsuki *et al*, 2012; Cenik *et al*, 2015; Cheng *et al*, 2016; Liu *et al*, 2016a; Eastman *et al*, 2018; preprint: Feltham *et al*, 2019; Wu *et al*, 2019; Zapalska‐Sozoniuk *et al*, 2019) means that causal relationships cannot be reliably inferred in any case. In our future work, we will determine the relative rates of protein synthesis and removal that generate rhythms in abundance. Once proteins are identified whose abundance rhythm is specifically attributable to a cell‐autonomous rhythm in translation, it will be possible to determine the relative contribution made by any circadian variation in cytosolic mRNA availability and its ribosomal recruitment. Whole‐cell transcriptomics alone cannot provide immediate mechanistic insight, since this does not distinguish sequestered mRNA from the pool that is actually available for translation (Eastman *et al*, 2018; Ivanov *et al*, 2019), nor does it account for the increase of mRNA stability that occurs during translation (Wu *et al*, 2019).

We do not exclude that circadian regulation of transcription occurs in CRY‐deficient cells; indeed, we have observed rhythms in the activity of the *Nr1d1* promoter in CKO cells, albeit under very specific culture conditions with < 5% the amplitude of WT controls (Putker *et al*, 2021). Rather, the increased number and amplitude of protein abundances and phosphorylation we observed in CRY‐deficient cells simply cannot be attributed to canonical CRY/PER‐mediated transcriptional feedback repression, since CRY is not present and PER does not repress BMAL1 complexes without CRY (Ye *et al*, 2014; Chiou *et al*, 2016). We also have not addressed the nature of the post‐translational mechanism postulated to generate circadian rhythms in mammalian cells, which is discussed elsewhere (Wong & O’Neill, 2018; Putker *et al*, 2021).

A further caveat to our study is that, for technical reasons, the primary fibroblasts used for the 3‐day (phospho)proteomics time course came from a single, but otherwise isogenic, WT and CKO mouse. Logically, a single observation of circadian (phospho)proteome regulation in CRY‐deficient cells is sufficient to refute the hypothesis that CRY is essential for circadian proteome regulation. Moreover, in our previous work, we demonstrated circadian rhythms in many independently generated CKO fibroblast lines and tissues, isolated from many different mice (Putker *et al*, 2021). Furthermore, analysis of published mouse liver data provides an independent validation of our finding that CRY can function to minimise, not generate, daily protein variation. Finally, the null hypothesis for this aspect of our analysis was either no rhythms or severely attenuated rhythms in CKO cells. This was rejected by analysis of both the initial mass spectrometry time course as well as the validation experiment, with multiple biological replicates (Fig 3), as well as by independent approaches (Fig 5).

It is quite plausible that the numbers, temporal profile and specific identity of many rhythmic (phospho)proteins vary between independently isolated lines due to stochastic heterogeneity and clonal expansion effects (Smith & Whitney, 1980; Phinney, 2012). Our validation experiment (Fig 3) suggests a ceiling effect here however, since the time‐dependent variation was ˜4 greater than sex‐dependent variation in WT fibroblasts, for example. It is also true that the numbers, identities and amplitudes of rhythmic proteins will inevitably vary somewhat, dependent on the method of analysis used to determine rhythmicity (Appendix Fig S1C). Rather than any specific protein, however, we have focused on the temporal regulation of the proteome and cellular composition as a whole, and it is not plausible that a different method of rhythmicity analysis would yield qualitatively different results for any of our experiments.

## Conclusion

We have shown that CRY‐dependent feedback mechanisms are not required for cell‐autonomous circadian rhythms of protein abundance in mammalian cells. Moreover, when CRY is present, it apparently functions to suppress at least as many abundance rhythms as it facilitates both *in vitro* and *in vivo*. Most importantly, CRY deficiency was associated with an overall imbalance of the proteome, associated with changes in protein stoichiometries, reduced proteasome activity, increased protein synthesis and overall protein levels. Cells adapt to this genetic insult by altering osmotic balance and proteome composition to achieve a different set point for protein homeostasis, with increased basal stress and sensitivity to proteotoxic stress. This likely contributes to the impairment of circadian timekeeping in CKO cells, tissues and mice and may also contribute to the (patho)physiological consequences of CRY deficiency in mice. We speculate that the principal utility of CRY‐mediated feedback repression is to couple global protein synthesis with degradation rates for most proteins, to minimise changes in protein abundance. This would ensure the energetically efficient temporal consolidation of proteome renewal during each day, whilst defending protein and osmotic homeostasis.

## Materials and Methods

### Mammalian cell culture

All animal work was licensed by the Home Office under the Animals (Scientific Procedures) Act 1986, with Local Ethical Review by the Medical Research Council and the University of Cambridge, UK. Fibroblasts homozygous for PER2::LUCIFERASE (Yoo *et al*, 2004) were extracted from adult mouse lung tissue and then serial passage was used as described previously to induce spontaneous immortalisation (Seluanov *et al*, 2010; Causton *et al*, 2015). Fibroblasts were cultured in Dulbecco’s modified Eagle’s medium (DMEM), supplemented with 100 units/ml penicillin, 100 µg/ml streptomycin (Gibco) and 10% FetalClone III serum (HyClone, Thermo Fisher). All cells were confirmed to be free of mycoplasma. Unless stated otherwise, confluent cell cultures up to a maximum of 30 passages were used during experiments to abolish any effects of cell division, since these cells display contact inhibition. For the first proteomics experiment, cells had been through 20 passages; for the second, cells had been through 6 passages.

### General statistics

*P* values are annotated in figures with asterisks, where the number of asterisks indicates the significance: Ns = not significant; *P ≤ 0.05; **P ≤ 0.01, ***P ≤ 0.001; ****P ≤ 0.0001. Technical replicates are denoted as “*n*” in the figures or figure legends (e.g. *n* = 3), and biological replicates are denoted as “*N*”. Statistical tests were carried out using Prism GraphPad 8 (San Diego, CA) or R v4.0.3.

### Longitudinal bioluminescent reporter experiments

Data from longitudinal bioluminescence recordings were analysed using Prism GraphPad 8 (San Diego, CA). A 24‐h moving average was used to detrend data, and a circadian damped cosine wave was fitted by least‐squares regression to determine period, phase and amplitude:

$$y=\left(mx+c\right)+ae^{‐kx}\cos\left(\frac{2\pi x‐r}{p}\right)$$

where *m* is the baseline gradient, *c* is the displacement in the *y* axis, *k* is the damping rate, *a* is the amplitude, *r* is the phase, and *p* is the period. The first 24 h of each recording were omitted because this represents the transient effects of medium change on clock gene expression. Rhythmicity of bioluminescence recordings was assessed by comparing the fit of this equation to the null hypothesis of a straight line using the extra sum‐of‐squares F‐test in Prism GraphPad 8 (San Diego, CA). If fitting to the damped cosine was preferred (*P* ≤ 0.05), then the recording was deemed “rhythmic”.

### Time course experiments: general structure

Cells were plated at a near‐confluent density (roughly 27,000 cells/cm^2^) and cultured in DMEM with 10% FetalClone III serum for one week in a temperature‐controlled incubator that was programmed to oscillate between 32°C and 37°C, with transitions every 12 h. The cells received a medium change at the transition between 37°C and 32°C after 4 days. After another 3 days, the cells received another medium change at the same transition time into medium containing either 10% or 1% serum, and the incubator was programmed to remain at 37°C constantly. At this time, a subset of cells received medium containing 1 mM luciferin, and these were placed into an ALLIGATOR for bioluminescent recording. After 24 h, sampling began, with 3‐hour intervals, and continuing for 3 days. The time point of the first sample is known as “Experimental time 0”, and all time points are reported relative to this. The nature of the sampling varied according to the specific experiment, and details are presented in separate sections.

### Proteomics and phosphoproteomics

#### Sample preparation

A time course was carried out as described above, with three technical replicates per genotype (i.e. 3 wells in a 6‐well plate) at each time point. At each time point, cells were washed twice in ice‐cold PBS and then lysed at room temperature in 100 µl lysis buffer (8 M urea, 20 mM Tris, pH 8) for 20 min. The lysis buffer was prepared the day before sampling began and frozen in 1 ml aliquots. At each time point, one aliquot was defrosted at room temperature (23°C) whilst shaking at 700 rpm for 5 min. After lysis, the cells were scraped and technical replicates were combined, thus creating 1 sample per genotype at each time point. These samples were flash‐frozen immediately after collection in liquid nitrogen and stored at −80°C. After the time course was completed, all the samples were simultaneously defrosted and sonicated for 2 min. The protein concentration was then measured using a BCA assay (Pierce). Twelve pooled samples were created by combining a portion of each experimental sample such that each sample/pool contained an equal mass of protein. All samples were then flash‐frozen in liquid nitrogen and stored at −80°C.

#### Enzymatic digestion

Each sample (256 µg) was reduced with 5 mM DTT at 56°C for 30 min and then alkylated with 10 mM iodoacetamide in the dark at room temperature for 30 min. They were then digested with mass spectrometry grade Lys‐C (Promega) at a protein:Lys‐C ratio of 100:1 (w/w) for 4 h at 25°C. Next, the samples were diluted to 1.5 M urea using 20 mM HEPES (pH 8.5) and digested at 30°C overnight with trypsin (Promega) at a ratio of 70:1 (w/w). Digestion was quenched by the addition of trifluoroacetic acid (TFA) to a final concentration of 1%. Any precipitates were removed by centrifugation at 13,000 *g* for 15 min. The supernatants were desalted using homemade C18 stage tips containing 3 M Empore extraction discs (Sigma) and 5 mg of Poros R3 resin (Applied Biosystems). Bound peptides were eluted with 30‐80% acetonitrile (MeCN) in 0.1% TFA and lyophilised.

#### TMT (Tandem mass tag) peptide labelling

The lyophilised peptides from each sample were resuspended in 100 µl of 2.5% MeCN, 250 mM triethylammonium bicarbonate. According to manufacturer’s instructions, 0.8 mg of each TMT 10‐plex reagent (Thermo) was reconstituted in 41 µl of anhydrous MeCN. The peptides from each time point and pooled sample were labelled with a distinct TMT tag for 75 min at room temperature. The labelling reaction was quenched by incubation with 8 µl 5% hydroxylamine for 30 min. For each set of 10‐plex TMT reagent, the labelled peptides from 8 time point samples + 2 pools were combined into a single sample and partially dried to remove MeCN in a SpeedVac (Thermo Scientific). After this, the sample was desalted as before and the eluted peptides were lyophilised.

#### Basic pH Reverse‐Phase HPLC fractionation

The TMT labelled peptides were subjected to off‐line high‐performance liquid chromatography (HPLC) fractionation, using an XBridge BEH130 C18, 3.5 μm, 4.6 mm × 250 mm column with an XBridge BEH C18 3.5 μm Van Guard cartridge (Waters), connected to an Ultimate 3000 Nano/Capillary LC System (Dionex). Peptide mixtures were resolubilised in solvent A (5% MeCN, 95% 10 mM ammonium bicarbonate, pH 8) and separated with a gradient of 1‐90% solvent B (90% MeCN, 10% 10 mM ammonium bicarbonate, pH 8) over 60 min at a flow rate of 500 μl/min. A total of 60 fractions were collected. They were combined into 20 fractions and lyophilised and desalted as before. 5% of the total eluate from each fraction was taken out for proteome LC‐MS/MS analysis and the rest was used for phosphopeptide enrichment.

#### Enrichment of phosphopeptides

All 20 fractions of peptide mixture were enriched first using PHOS‐Select iron affinity gel, an Iron (III) Immobilised Metal Chelate Affinity Chromatography (IMAC) resin (Sigma). Desalted peptides were resuspended in 30% MeCN, 0.25 M acetic acid (loading solution) and 30 µl of IMAC beads, previously equilibrated with the loading solution, was added. After 60 min incubation at room temperature, beads were transferred to a homemade C8 (3 M Empore) stage tip and washed 3 times with loading solution. Phosphopeptides were eluted sequentially with 0.4 M NH_3_, 30% MeCN, 0.4 M NH_3_ and 20 µl of 50% MeCN, 0.1% TFA.

The flow through from the C8 stage tips was collected and combined into 10 fractions and used for titanium dioxide (TiO_2_) phosphopeptide enrichment. For this, the total volume of flow through was made up to 50% MeCN, 2 M lactic acid (loading buffer) and incubated with 1–2 mg TiO_2_ beads (Titansphere, GL Sciences, Japan) at room temperature for 1 h. The beads were transferred into C8 stage tips, washed in the tip twice with the loading buffer and once with 50% MeCN, 0.1% TFA. Phosphopeptides were then eluted sequentially with 50 mM K_2_HPO_4_ (pH 10) followed by 50% MeCN, 50 mM K_2_HPO_4_ (pH 10) and 50% MeCN, 0.1% TFA.

The first 10 fractions of IMAC and the 10 fractions of TiO_2_‐enriched phosphopeptides were combined, and the other 10 fractions from IMAC enrichment were combined into 5 fractions, thus making a total of 15 fractions for phosphoproteomics analysis. Phosphopeptide solution from these fractions was acidified, partially dried and desalted with a C18 stage tip that contained 1.5 µl of Poros R3 resin. These were then partially dried again and thus ready for mass spectrometry analysis.

#### LC MS/MS

The fractionated peptides were analysed by LC‐MS/MS using a fully automated Ultimate 3000 RSLC nano System (Thermo) fitted with a 100 μm × 2 cm PepMap100 C18 nano trap column and a 75 μm × 25 cm reverse phase C18 nano column (Aclaim PepMap, Thermo). Samples were separated using a binary gradient consisting of buffer A (2% MeCN, 0.1% formic acid) and buffer B (80% MeCN, 0.1% formic acid) and eluted at 300 nl/min with an acetonitrile gradient. The outlet of the nano column was directly interfaced via a nanospray ion source to a Q Exactive Plus mass spectrometer (Thermo). The mass spectrometer was operated in standard data‐dependent mode, performing a MS full‐scan in the m/z range of 350–1,600, with a resolution of 70,000. This was followed by MS2 acquisitions of the 15 most intense ions with a resolution of 35,000 and Normalised Collision Energy (NCE) of 33%. MS target values of 3e6 and MS2 target values of 1e5 were used. The isolation window of precursor ion was set at 0.7 Da and sequenced peptides were excluded for 40 s.

#### Spectral processing and peptide and protein identification

The acquired raw files from LC‐MS/MS were processed using MaxQuant (Cox and Mann) with the integrated Andromeda search engine (v1.6.3.3). MS/MS spectra were quantified with reporter ion MS2 from TMT 10‐plex experiments and searched against the *Mus musculus* UniProt Fasta database (Dec 2016). Carbamidomethylation of cysteines was set as fixed modification, while methionine oxidation, N‐terminal acetylation and phosphorylation (STY) (for phosphoproteomics group only) were set as variable modifications. Protein quantification requirements were set at 1 unique and razor peptide. In the identification tab, second peptides and match between runs were not selected. Other parameters in MaxQuant were set to default values.

The MaxQuant output file was then processed with Perseus (v1.6.2.3). Reporter ion intensities were uploaded to Perseus. The data were filtered: identifications from the reverse database were removed, only identified by site, potential contaminants were removed, and we only considered proteins with ≥ 1 unique and razor peptide. Then, all columns with an intensity “less or equal to zero” were converted to “NAN” and exported. The MaxQuant output file with phosphor (STY) sites table was also processed with Perseus software (v1.6.2.3). The data were filtered: identifications from the reverse database were removed, only identified by site, potential contaminants were removed and we only considered phosphopeptides with localisation probability ≥ 0.75. Then, all columns with intensity “less or equal to zero” were converted to “NAN” and exported.

#### Bioinformatics

All data handling was done using R v3.6.3. Since the sample for time point 12 was missing for CRY1^−/−^; CRY2^−/−^, abundance values were inferred for each protein by taking the mean of the two neighbouring time points. WT and CKO datasets were analysed either combined or independently. The combined analysis was used to directly compare protein and phosphopeptide abundances between genotypes, since the internal reference scaling normalisation accounts for batch effects. The independent method was used for all other analysis that did not require comparison of abundance, thus allowing the detection of proteins that were present in one genotype but not the other.

Proteins and phosphopeptides were only accepted for further analysis if present in all time points and pooled samples. Hence, in the combined analysis, proteins/phosphopeptides had to be present in all time points for both genotypes, as well as all pooled samples. In the independent analysis, proteins/phosphopeptides had to be present in all time points and pools for one genotype only. Sample loading normalisation was carried out by taking the sum of all intensities for each time point and normalising to the mean of these, since an equal amount of protein was used for each TMT labelling reaction. This was followed by internal reference scaling (IRS) to allow for comparisons between TMT experiments (Plubell *et al*, 2017): for each TMT 10‐plex set, the mean abundance for each protein in both pools was calculated. Then, the mean of these means was calculated and used to normalise the values for each protein for all the samples.

Rhythmicity was tested using the RAIN (Rhythmicity Analysis Incorporating Non‐parametric methods) algorithm (Thaben & Westermark, 2014), and multiple testing was corrected for using the adaptive Benjamini–Hochberg method. Proteins with a corrected *P* ≤ 0.05 were deemed significant. Relative amplitude of rhythmic proteins was calculated by detrending the data using a 24‐h moving average and dividing the resultant range by the average normalised protein abundance. To include only proteins with a biologically relevant level of oscillation, only those with relative amplitude ≥ 10% were taken for further analysis (see text for details). Phosphoproteomics data were handled in the same way, except that normalised phosphopeptide abundances were adjusted according to the changes in abundance of the corresponding protein from the normalised total proteome data, and no threshold for relative amplitude was used.

Gene ontology analysis was performed using the GOrilla online tool (Eden *et al*, 2007, 2009). Analysis was performed either as a single ranked list of gene names, or as a target dataset compared to background (all proteins detected in the experiment). Kinase recognition motifs were screened using a custom script written in Python v2.7, which used the PHOSIDA database (Gnad *et al*, 2007, 2011).

### Validation proteomics experiment

For the 16‐plex TMTpro proteomics experiment, lung fibroblasts were isolated from 8 adult mice (4 WT and 4 CKO) and plated after 6 passages into 6‐well plates, with 3 technical replicates per cell line for each time point. Cells were grown in temperature cycles (12 h 37°C and 12 h 32°C) for 14 days, with medium changes every 4 days. After 14 days, at the transition to 32°C cells received a medium change into medium containing 1% serum and moved into constant 37°C. Cells were harvested using the same conditions as the 3‐day time course experiment, at 24 h and 36 h after the medium change. 60 µg of each sample was used for enzymatic digestion, 16‐plex TMTpro labelling (Thermo), HPLC fractionation and LC MS/MS, as described above.

Bioinformatic analysis was carried out using R v4.1.1 and Prism v9.2.0. Sample loading normalisation was carried out by normalising to the sum of all intensities per time point. To identify proteins that were significantly altered, 2‐way ANOVAs were carried out between the categories of interest (WT vs. CKO, WT vs. time, CKO vs. time, WT male vs. female), followed by two‐stage linear step‐up procedure of Benjamini, Krieger and Yekutieli multiple comparisons analysis. Proteins with *P* ≤ 0.05 were deemed significantly altered in abundance.

### Western blotting

For Western blots, proteins were run on NuPAGE™ Novex™ 4–12% Bis‐Tris Protein Gels (Thermo Fisher) before transferring to nitrocellulose membranes. For transfer, the iBlot system (Thermo Fisher) was used. Membranes were blocked using 5% milk powder (Marvel) or 0.125% BSA (Sigma) and 0.125% milk powder (Marvel) in TBS containing 0.1% Tween‐20 (TBST) for 30 min at room temperature then incubated with primary antibody at 4°C overnight. HRP‐conjugated secondary antibodies (Thermo Fisher) diluted 1:10,000 in blocking buffer were incubated with the blots for 1 h at room temperature. Chemiluminescence was detected in a Bio‐Rad chemidoc using Immobilon reagent (Millipore). Protein loading was checked by staining gels with Colloidal Coomassie Blue Stain (Severn Biotech). Densitometric analysis was carried out using Image Lab 4.1 (Bio‐Rad Laboratories).

### Measurement of cellular protein content

At specified time points, confluent monolayers of cells were washed twice with ice‐cold PBS. Cells were then incubated with 200 µl digitonin lysis buffer (50 mM Tris pH 7.4, 0.01% digitonin, 5 mM EDTA, 150 mM NaCl, 1 U/ml benzonase, protease and phosphatase inhibitors) on ice for 15 min before lysates were collected. For total protein extraction, cells were instead incubated with 200 µl RIPA buffer (50 mM Tris pH 7.4, 1% SDS, 5 mM EDTA, 150 mM NaCl, 1 U/ml benzonase, protease and phosphatase inhibitors), on ice for 15 min. Cells lysed with RIPA buffer were then scraped and collected, and all samples were flash‐frozen in liquid nitrogen. After thawing, RIPA lysates were sonicated at high power for 10 s at 4°C to shear genomic DNA. RIPA lysates and digitonin lysates were clarified by centrifugation at 21,000 *g* for 15 min at 4°C.

Intrinsic tryptophan fluorescence was used to measure protein concentrations. 10 µl of each sample was transferred into a UV‐transparent 384‐well plate (Corning 4681) in quadruplicate. After brief centrifugation of the plate, quantification was carried out using a Tecan Spark 10 M microplate reader, with excitation at 280 nm and emission at 350 nm. Standards were made using bovine serum albumin (Fisher Scientific), dissolved using the same lysis buffer as the lysates being measured. Standard curves were fitted to a quadratic curve using Prism GraphPad 8 (San Diego, Ca), and protein concentrations were interpolated.

### Measurement of intracellular ion content by Inductively Coupled Plasma – Mass Spectrometry (ICP‐MS)

Confluent monolayers of cells were washed on ice with iso‐osmotic buffer A (300 mM sucrose, 10 mM Tris base, 1 mM EDTA, pH 7.4 adjusted with phosphoric acid, 330–340 mOsm adjusted with sucrose/HPLC water), followed by iso‐osmotic buffer B (300 mM sucrose, 10 mM Tris base, 1 mM EDTA, pH 7.4 adjusted with acetic acid, 330–340 mOsm adjusted with sucrose/HPLC water). Iso‐osmotic buffer A contains phosphoric acid which displaces lipid bound ions. Iso‐osmotic buffer B contains acetic acid which removes traces of phosphates. Cells were then incubated for 30 min at room temperature in 200 µl ICP‐MS cell lysis buffer (65% nitric acid, 0.1 mg/ml (100 ppb) cerium). Lysates were then collected and stored at −80°C. All samples were thawed simultaneously and diluted using HPLC water to a final concentration of 5% nitric acid. Diluted samples were analysed by inductively coupled plasma–mass spectrometry (ICP‐MS) using the NexION 350D ICP‐MS (PerkinElmer Inc.) as described previously (Feeney *et al*, 2016).

### Proteasome activity assays

20,000 cells per well were plated in a 96‐well plate using standard culture medium, and the medium was changed 9 h later. 10 µM epoxomicin in the medium was used as negative control. 3 h after this medium change/epoxomicin treatment, the ProteasomeGlo Cell‐Based Assay (Promega) was used to measure proteasome catalytic activity. Chymotrypsin‐like, trypsin‐like and caspase‐like activities were measured separately using the relevant substrates from Promega (Suc‐LLVY‐Glo, Z‐LRR‐Glo, Z‐nLPnLD‐Glo respectively). Assay reagents were prepared according to the manufacturer’s instructions. The 96‐well plate was equilibrated to room temperature, and a volume of assay reagent equal to the volume of medium was added to each well before shaking at 700 rpm for 2 min. The plate was incubated at room temperature for a further 10 min, and then, luminescence was measured using the Tecan Spark 10 M microplate reader, recording counts for 1 s. The luminescence readings from the epoxomicin controls represent background protease activity, and so this was subtracted from all other recordings.

### Measurement of translation rate

Wild‐type and CKO mouse lung fibroblasts were grown to confluence in 48‐well plates, and medium was changed 24 h before the experiment to either low (1%) or high (10%) serum. The cells were pulsed with 0.1 mCi/ml ^35^S‐L‐methionine/^35^S‐L‐cysteine mix (EasyTag™ EXPRESS35S Protein Labeling Mix, Perkin Elmer) in cysteine/methionine‐free DMEM for 15 min at 37°C, with or without serum supplement. Afterwards, cells were washed with ice‐cold PBS and lysed in digitonin‐based buffer (with protease inhibitor tablet, added freshly) on ice. Lysates were reduced with LDS buffer and run on 4‐12% Bis–Tris SDS–PAGE using MES buffer. Gels were then dried at 80°C and exposed overnight to a phosphorimager screen. Images were acquired with Typhoon FLA700 gel scanner and quantified using Fiji.

For the puromycin pulse experiment, cells were seeded (in the presence of 1 mM luciferin) in fibronectin‐coated dishes at high density one day before starting the experiment. Separate 6‐well plates were used for each time point. 30 min before the start of the experiment, cells were synchronised by a dexamethasone pulse (10 nM), after which cells were exchanged into air medium (DMEM without glucose, l‐glutamine, phenol red, sodium pyruvate and sodium bicarbonate (Sigma). The following was added: 0.35 g/l sodium bicarbonate (Sigma), 5 g/l glucose (Sigma), 20 mM MOPS (VWR), penicillin/streptomycin solution (as above), Glutamax (Thermo Fisher), B27 (Thermo Fisher), 1 mM potassium luciferin solution (Biosyth), 1% FetalClone III serum. The medium was adjusted to pH 7.6 at room temperature, and osmolality adjusted to 350–360 mOsm/kg using sodium chloride). At the indicated time points, 10 µg/ml puromycin was added while keeping cells warm on a hot plate, after which the plate was incubated in tissue culture incubator for exactly 10 min. Time point 0 represents cells just before the dexamethasone pulse. Cells were then washed with ice‐cold PBS, after which they were lysed in lysis buffer (50 mM Tris, pH 7.5, 1% Triton X‐100, 0.1% SDS, 1.5 mM MgCl_2_, 100 mM NaCl, cOmplete protease inhibitor cocktail (Roche)) and flash‐frozen. For Western blot analysis, samples were spun down, diluted in Laemmli sample buffer, ran on 4–20% gradient gels, and blots were probed for ATF4 and tubulin.

### Animal work

Cry1/2‐null mice were kindly provided by G.T. van der Horst (Erasmus MC, Rotterdam, the Netherlands) (van der Horst *et al*, 1999). All animals were housed in 12:12‐h light:dark conditions. Post‐mortem histopathology examinations were carried out by Abbey Veterinary Services, UK. For the *in vivo* stress marker experiment, animals received an IP injection of bortezomib (Generon) (2.5 mg/kg) or vehicle control (1% DMSO in sterile PBS) 2 h after the transition to the light phase. 5 h later, the animals were culled and lungs were immediately flash‐frozen in liquid nitrogen. Tissues were homogenised on ice in RIPA buffer (as above) and flash‐frozen. After thawing these samples on ice, they were sonicated briefly at 4°C. Samples were clarified by centrifugation and protein diluted to 2 mg/ml. Bolt/NuPage loading buffer with 50 mM TCEP (final concentration) was added and reduced for 10 min at 70°C. Western blots were then carried out as described above, probing against phospho‐STAT3 (Tyr705) or actin. One female mouse and one male mouse of each genotype were used in this experiment.

For the proteasomal inhibition experiment, mice were individually housed in a temperature and humidity‐controlled circadian cabinet (Phenome Technologies) and drinking water was supplemented with 10% blackcurrant and apple squash (Co‐operative Foodstores Limited) to mask the taste of the drug. Locomotor activity was monitored by wheel‐running detection and analysed using ClockLab (Actimetrics). Mice were entrained using 12‐h light (400 lux), 12‐h dark (LD) cycles for the first 7 days.

### Antibodies

**Table:** 

Antibody	Host	Cat. #	Manufacturer	Dilution
Anti‐mouse‐HRP	Goat	A4416	Sigma	1:10,000
Anti‐rabbit‐HRP	Goat	A6154	Sigma	1:10,000
Anti‐rat‐HRP	Goat	629520	Thermo	1:10,000
Tubulin YL1‐2	Rat		in‐house	1:100
β‐Actin	Mouse	sc‐47778	Santa‐Cruz Biotechnology	1:1,000
Proteasome 20S α1‐7	Mouse	ab22674	Abcam	1:2,000
EIF2α	Mouse	AhO0802	Thermo	1:1,000
P‐EIF2α (S51)	Rabbit	ab32157	Abcam	1:1,000
P‐STAT3 (Tyr705)	Rabbit	D3A7	Cell Signaling	1:1,000
Histone H3	Rabbit	ab1791	Abcam	1:10,000

## Author contributions

DCSW, ES and JSON designed the study, analysed the data and wrote the manuscript; DCSW, ES, AS, AZ, CTS, RSE and MP performed cell experiments; SYP‐C and JD performed mass spectrometry analyses; DCSW, NMR, ADB and JSON performed mouse studies; MR performed tissue collection and husbandry. All authors commented on the manuscript.

## Conflict of interest

The authors declare that they have no conflict of interest.

## Supporting information



Appendix

Source Data for Figure 6

## Data Availability

Scripts and processed data are available on GitHub (https://github.com/davwong47/Circadian‐proteomics). Mass spectrometry data have been deposited to the ProteomeXchange Consortium (Deutsch *et al*, 2019) via the PRIDE (Perez‐Riverol *et al*, 2018) partner repository with the dataset identifier PXD019499 (http://www.ebi.ac.uk/pride/archive/projects/PXD019499).
