# Supplementary material for: CRYPTOCHROMES promote daily protein homeostasis
Source: EMBO J. 2021 Nov 29;41(1):e108883. doi: 10.15252/embj.2021108883 (PMC8724739; doi:10.15252/embj.2021108883)

Appendix

Table of Contents

Appendix figure legends....... 2
Appendix references....... 10
Appendix figures....... 11

Appendix figure legends

Appendix Figure S1, Relating to Figure 1. Examples and analysis of 3-day WT and CKO (phospho)proteomics time courses. A) Post-hoc analysis of proteomics data from Mauvoisin et al. (Mauvoisin *et al*, 2014). Volcano plot showing the fold change in average expression of all proteins, in CKO livers compared to WT (q = Benjamini-Hochberg corrected p-value). Statistically significant changes ($q \leq 0.05$) are shown in red. Some proteins are labelled as space allows. **B)** The relative abundance of CRY1 detected in the 3-day WT dataset is plotted. It was preferentially fit by a damped cosine wave over a straight line (Extra sum-of-squares F test, p=0.01). CRY1 was the only "clock protein" detected in WT samples, and was not detected in any CKO samples. Below: Longitudinal bioluminescence recording of WT and CKO PER2::LUC fibroblasts, performed simultaneously with the proteomics experiment as a phase marker. Mean±SEM. **C)** RAIN and eJTK were compared as statistical tests for rhythmicity. Top, the results of eJTK analysis showing that the relative numbers of rhythmic proteins and the overlap between WT and CKO time courses were similar to the results from the RAIN analysis in Figure 1. **D), E)** Heatmaps showing min-max normalised plots for all the rhythmic proteins (D) and phosphopeptides (E) in WT and CKO cells. For each genotype separately, rows represent proteins sorted by phase, and each column is a time point from the timecourse experiment. One missing sample is annotated in black. **F), G)** Examples of proteins (F) and phosphopeptides (G) detected by mass spectrometry are shown, labelled by gene name and phosphosite. Examples are included of proteins/phosphopeptides that are rhythmic in both genotypes, rhythmic in only one genotype, or not rhythmic in either. P-values shown are from a comparison of fit (F test, damped cosine against straight line). Annotation as rhythmic/arrhythmic are from the RAIN output.

Appendix Figure S2, Relating to Figures 1 & 2. Summaries of 3-day proteomics time course analysis.

A) Table summarising the numbers of proteins detected in both genotypes with significantly increased or decreased abundance (corrected $p<0.05$ vs. $p\geq0.05$) and significant change in rhythmicity (RAIN $p<0.05$ vs. $p\geq0.05$) between the first set of WT and CKO proteomics time courses. If it is assumed that CRY is required for canonical TTFL function, then rhythms in the abundance of at least as many proteins are suppressed by the TTFL (green) than are dependent upon it (blue). Most noticeably, 82% of detected proteins in CKO cells were altered in average abundance compared with WT (Abundance up + down), suggesting that the respective synthesis and degradation rates of very many proteins are altered by CRY deficiency. We postulated that CRY deletion might unmask rhythmicity in proteins that normally exhibit matched rhythms of synthesis and degradation in WT cells, i.e. constitutively present with rhythmic turnover. Supporting this, there was a significant association between changes in average protein abundance and changes in rhythmicity (Fisher's exact test, $p=0.04$), i.e., proteins that changed in overall abundance were more likely to change in rhythmicity: from arrhythmic to become rhythmic, or *vice versa*. If stated assumptions and RAIN rhythmicity results for these time courses are taken at face value, it would suggest that ~ 30% of the fibroblast proteome is subject to cell-autonomous circadian regulation (green + blue + brown), with CRY primarily acting to suppress rhythms in the abundance of most of these proteins (green).

B) Absolute fold changes were calculated from maximum/minimum for each protein and plotted as probability density curves to illustrate the extent of proteome variation within a genotype across time or in maximum/minimum abundance between genotypes; medians are annotated. WT vs time was significantly different to WT vs CKO ($p<0.0001$) but not significantly different to CKO vs time ($p=0.5586$) by Kruskal-Wallis with Dunnett's MCT. Note that the proteome-wide distribution of temporal variation was very similar between WT and CKO time courses; whereas major differences were observed between genotypes both in proteome composition and in the identity of temporally-regulated proteins, as reported in Figure 1.

Appendix figure S3, Relating to Figures 1 & 2. Summaries of 3-day phosphoproteomics time course analysis.

To test the effects of CRY deletion on the phosphoproteome, we performed the same analysis as in Figure S2 with detected phosphopeptides. **A)** Table summarising the numbers of phosphopeptides detected in both genotypes with significantly increased or decreased abundance (corrected $p<0.05$ vs. $p\geq0.05$) and significant change in rhythmicity (RAIN $p<0.05$ vs. $p\geq0.05$) between the WT and CKO time courses. If it is assumed that CRY is required for canonical TTFL function, then at least as many rhythms in protein phosphorylation are suppressed by the TTFL (green) than are facilitated by it (blue). Most noticeably, 68% of detected phosphopeptides in CKO cells were altered in average abundance compared with WT (Abundance up + down), suggesting that the respective phosphorylation and dephosphorylation rates at many phospho-sites are altered by CRY deficiency. We postulated that CRY deletion might unmask rhythmicity in phosphosites that normally exhibit matched rhythms of phosphorylation and dephosphorylation in WT cells, i.e. they exhibit a constant level of phosphorylation but with rhythmic phosphate turnover. Supporting this, there was a significant association between changes in average phosphopeptide abundance and changes in rhythmicity (Fisher's exact test, $p=0.0009$), i.e., phosphopeptides that changed in overall abundance were more likely to change in rhythmicity: from arrhythmic to become rhythmic, or *vice versa*. If the stated assumptions and RAIN rhythmicity results for these time courses are taken at face value, it would suggest that ~ 21% of the detected fibroblast phosphoproteome is subject to cell-autonomous circadian regulation (green + blue + brown), with CRY primarily acting to (indirectly) suppress rhythms in steady state phosphorylation in the majority of cases (green). This possibility requires further validation but certainly merits future investigation.

B) Absolute fold changes were calculated from maximum/minimum for each phosphopeptide and plotted as probability density curves to illustrate the extent of phosphoproteome variation within a

genotype across time or in maximum/minimum abundance between genotypes; medians are annotated. WT vs time was significantly different to both WT vs CKO ($p<0.0001$) and CKO vs time ($p<0.0001$) by Kruskal-Wallis with Dunnett's MCT. Please note that, though a statistically significant difference was observed, the phosphoproteome-wide distribution of temporal variances were very similar between WT and CKO time courses, i.e., the major differences were observed between genotypes both in phosphoproteome composition and in the identity of temporally-regulated phosphopetides, as reported in Figure 1..

Appendix figure S4, Relating to Figures 1 & 2. Increased protein phosphorylation and reduced protein phosphatase expression in CRY-deficient compared with WT cells.

A) Fold-change in abundance was calculated for each phosphopeptide. There was a significant upregulation of overall phosphorylation in CKO cells compared to WT, across all timepoints (One sample t test, $p<0.0001$, n=2803). **B)** We therefore considered whether CRY deletion might impact upon the abundance or activity of protein kinases and phosphatases, which act in dynamic equilibrium to determine the phosphorylation level of each peptide. Fold-change in abundance was calculated for protein kinases (top) and protein phosphatases (bottom) detected in the proteomics dataset. There was a significant downregulation of phosphatase abundance in CKO cells compared to WT (One sample t test, p=0.002, n=39), but no overall change in kinase abundance (One sample t test, p=0.7, n=182). **C), D), E)** To validate this, we employed the PHOSIDA database of kinase binding motifs (Gnad et al, 2011, 2007) to infer rhythmic kinase activity using the phosphopeptides sequences we detected. We found that targets of kinases typically associated with circadian regulation were not over-represented in WT or CKO rhythmic datasets compared to background, nor in the set of phosphopeptides that were rhythmic in both genotypes. Phosphopeptides that were rhythmic (in WT, in CKO, or in both genotypes respectively, blue) were compared to the background of phosphopeptides present in all samples and pools (red). Thus, if phosphatase availability is ever rate-limiting for protein dephosphorylation, then both the increased overall

phosphorylation and increased rhythmic phosphorylation in CKO compared with WT cells could plausibly result from their reduced level of protein phosphatase expression. If validated, this would suggest that normally CRY ultimately functions to globally supress steady-state protein phosphorylation, as well as rhythms in protein phosphorylation, in part through regulation of phosphatase abundance.

Discussion: In our kinase inference analysis, we did not find evidence supportive of circadian phosphorylation by any of the other kinases normally implicated in the post-translational circadian regulation, such as CK1. The very poor overlap in rhythmically phosphorylated proteins between the two genotypes, and between rhythmic phosphorylation and rhythmic protein in either genotype, suggests that the circadian functions of this post-translational modification are likely to be context-dependent. As with the proteome, however, we did notice a highly significant association between changes in phosphopeptides rhythmicity and overall abundance between the two genotypes (Figure S3A). If a similar interpretation to that which we propose for the proteome were true, as much as 20% of protein phosphorylation is subject to cell-autonomous circadian regulation (Figure S3A), i.e., in most cases, rhythmic phosphorylation is matched by a dephosphorylation rhythm of similar phase and amplitude. Given the apparent consolidation of phosphorylation rhythms in WT cells around a temporal window that anticipates the active phase (Figure 2I vs 2E), also observed *in vivo*, we speculate that the co-ordinated and phase-coherent circadian regulation of phosphorylation and dephosphorylation at many phosphosites, without change in their steady state phosphorylation level, might confer more sensitive and rapid transduction of a given extracellular stimulus when received around the rest-to-active transition compared with 12 hours later. Future work will be required to test this hypothesis experimentally.

Appendix Figure S5, Relating to Figure 4. Differences in proteasome expression and protein synthesis between CRY-deficient and WT cells.

A) The abundance of proteasomal catalytic subunits detected in the proteomics experiment are plotted as time series. These are *Psmb1*, *Psmb2* and *Psmb5*, corresponding to caspase-like, trypsin-like and chymotrypsin-like subunits respectively. P values from RAIN analysis are shown, and linear regression lines are shown for reference. **B)** Whole images of Western blots shown in Figure 4B, probing for alpha subunits of the 20S proteasome (left) and Histone H3 (right). Samples were taken at 12h and 36h after a medium change. Only samples taken at 36h are shown in Figure 4B because those from 12h are likely to represent an acute response to the medium change. **C)** ^{35}S-methionine incorporation was used to measure translation rate in cultured WT and CKO cells. This was carried out at 0% and 10% serum. 4 replicates are shown, run on the same gel. An image of the phosphor screen is shown above, with the corresponding Coomassie stain below. The condition with 10% serum is shown in Figure 4E as this represents normal culture conditions. **D)** Quantification of (C). 2-way ANOVA showed interaction between the effects of serum and genotype (p=0.045). Holm-Sidak multiple comparisons results are shown as asterisks. N=3 experiments, n=4 technical replicates.

Appendix Figure S6, Relating to Figure 5. Increased rhythmic ion transporter expression and activity in CRY-deficient compared with WT cells.

A), B) 29 proteins were annotated as “Ion transport” by the GO analysis from the proteomics experiment – relative amplitude and average abundance was calculated for each of these proteins, in both WT and CKO. On average, both relative amplitude and average abundance was increased in CKO cells compared to WT (Paired t test). **c)** Examples of ions detected from both WT and CKO cells by ICP-MS are shown. Mean±SEM, F test for comparison of fit between damped cosine and straight line, N=3. The preferred fit is shown in red.

Appendix Figure S7, Relating to Figure 6. Increased stress in CRY-deficient cells and reduced growth/viability in CRY-deficient mice.

A) Volcano plot showing the fold change in average expression of all proteins in CKO cells compared to WT (*q* = Benjamini-Hochberg corrected p-value). Proteins annotated as “Response to stress” from GO analysis are highlighted in red, showing that these are upregulated in CKO cells. **B), C)** Growth curves of male and female mice were weighed weekly. Mice were of the following genotypes: WT, CRY1$^{-/-}$; CRY2$^{+/-}$ and CRY1$^{-/-}$; CRY2$^{-/-}$ (CKO). F test was used to test the null hypothesis that one curve fits all sets. P values annotated as asterisks. **D)** Food consumption measured over 1 week, normalised for mouse weight. Food consumption was monitored by weighing food daily. Mean±SD, 2-way ANOVA. **E)** Death rates among the 3 genotypes mentioned above, expressed as a percentage of the number of mice. Mice were fed *ad libitum* and group-housed under standard 12:12 light:dark conditions. The absolute numbers of deaths and total population size are annotated on the bars. Only mice that had been weaned were included, and unnatural causes of death (e.g. cage flooding, fighting) were excluded. Asterisk indicates significance from Chi-squared test for trend, p=0.007. Comparing WT and CKO, Fisher’s exact test p=0.009. Comparing WT and Het, Fisher’s exact test p=0.5. Median age of death for WT mice = 4 weeks, CRY1$^{-/-}$; CRY2$^{+/-}$ = 4 weeks, CRY1$^{-/-}$; CRY2$^{-/-}$ (CKO) = 7 weeks, p=0.4 for differences between groups, Kruskal-Wallis test.

Appendix Figure S8, Relating to Figure 7. A refined model for the generation and utility of cellular circadian timekeeping that may account for phenotypes associated with CRY-deficiency.

A) The canonical model for circadian timekeeping and the effect of the CRY1$^{-/-}$;CRY2$^{-/-}$ (CKO) genotype. The transcriptional-translational feedback loop (TTFL) is the mechanistic basis by which circadian rhythms in clock protein activity are generated. This leads to rhythmic expression of clock-controlled genes and encoded proteins, ultimately facilitating the circadian coordination of cell biology and whole-organism physiology; depicted by sinusoidal curves in black. CRY proteins are essential components of the TTFL, and without them it cannot function. In consequence, there is

no circadian regulation of cellular function, organismal physiology or behaviour (flat line, red). Absence of circadian rhythms leads to the deleterious phenotypes of CKO mice and cells, such as altered metabolism, increased carcinogenic potential and death.

B) A proposed refinement to the canonical model where blue elements are present in both WT and CKO, black elements in WT only, and red elements are consequences of CRY1$^{-/-}$;CRY2$^{-/-}$ (CKO) genotype. We suggest the principal utility of the canonical TTFL is to minimise, not generate, daily variation in protein abundance, whilst allowing daily cycles of protein activity and proteome renewal that function to maintain overall protein and osmotic homeostasis over time. In this model, circadian timing results from a cytosolic post-translational oscillator, or "cytoscillator", involving enzymes such as casein kinase 1, as in mammalian erythrocytes and other eukaryotic cells. The cytoscillator is sufficient to confer post-translational circadian regulation upon protein activity, abundance, and compensatory ion transport, but its timing mechanism is not robust against proteotoxic stresses and other extracellular perturbations. PERIOD proteins are the primary vector of timing information from the cytosol to the nucleus. The cytoscillator confers daily rhythms upon the activity of PERIOD as well as CRY, BMAL1 and other promiscuous transcription/translation factors proteins, which act to anticipate and buffer against changes in protein abundance, both directly and *via* clock-controlled gene regulation. This gives rise to circadian regulation of chromatin architecture and the expression of genes including *Period1/2* and *Cry1/2* seen in WT cells, and confers robustness to circadian rhythms by preventing proteome imbalance and proteotoxic stress, as well as by hysteresis *via* PERIOD1/2 abundance rhythms (Wong & O'Neill, 2018). In consequence, many proteins are circadian regulated in their activity and synthesis, but relatively few proteins show biologically significant change in their overall abundance. Cells adapt to the absence of CRY by remodelling of the (phospho)proteome and ionome, at the cost of increased cellular stress, which underlies the many adverse phenotypes of the CKO genetic model. One such adverse phenotype is a severe impairment to the temporal co-ordination of physiology and behaviour over circadian timescales. CKO cells possess the capacity for circadian timekeeping but,

without the TTFL to temporally buffer protein homeostasis, adaptations to CRY-deletion are effectively epistatic to the expression of circadian rhythms *in vivo* under most conditions. The pathways linking CRY with proteome imbalance are very likely multiple and indirect, given the many well-characterised functions of CRY proteins. This model is an extension of that proposed by Putker et al (Putker *et al*, 2021). In future work, we will explore the evidence for post-translational circadian regulation of compartmentalisation, translation and proteome renewal.

Appendix references

Gnad F, Gunawardena J & Mann M (2011) PHOSIDA 2011: The posttranslational modification database. *Nucleic Acids Res* 39: 253–260

Gnad F, Ren S, Cox J, Olsen J V., Macek B, Oroshi M & Mann M (2007) PHOSIDA (phosphorylation site database): Management, structural and evolutionary investigation, and prediction of phosphosites. *Genome Biol* 8: R250

Mauvoisin D, Wang J, Jouffe C, Martin E, Atger F, Waridel P, Quadroni M, Gachon F & Naef F (2014) Circadian clock-dependent and -independent rhythmic proteomes implement distinct diurnal functions in mouse liver. *Proc Natl Acad Sci U S A* 111: 167–72

Putker M, Wong DCS, Seinkmane E, Rzechorzek NM, Zeng A, Hoyle NP, Chesham JE, Edwards MD, Feeney KA, Fischer R, *et al* (2021) CRYPTOCHROMES confer robustness, not rhythmicity, to circadian timekeeping. *EMBO J* e106745: 1–15

Wong DC & O'Neill JS (2018) Non-transcriptional processes in circadian rhythm generation. *Curr Opin Physiol* 5: 117–132

Appendix figures

Appendix Figure S1

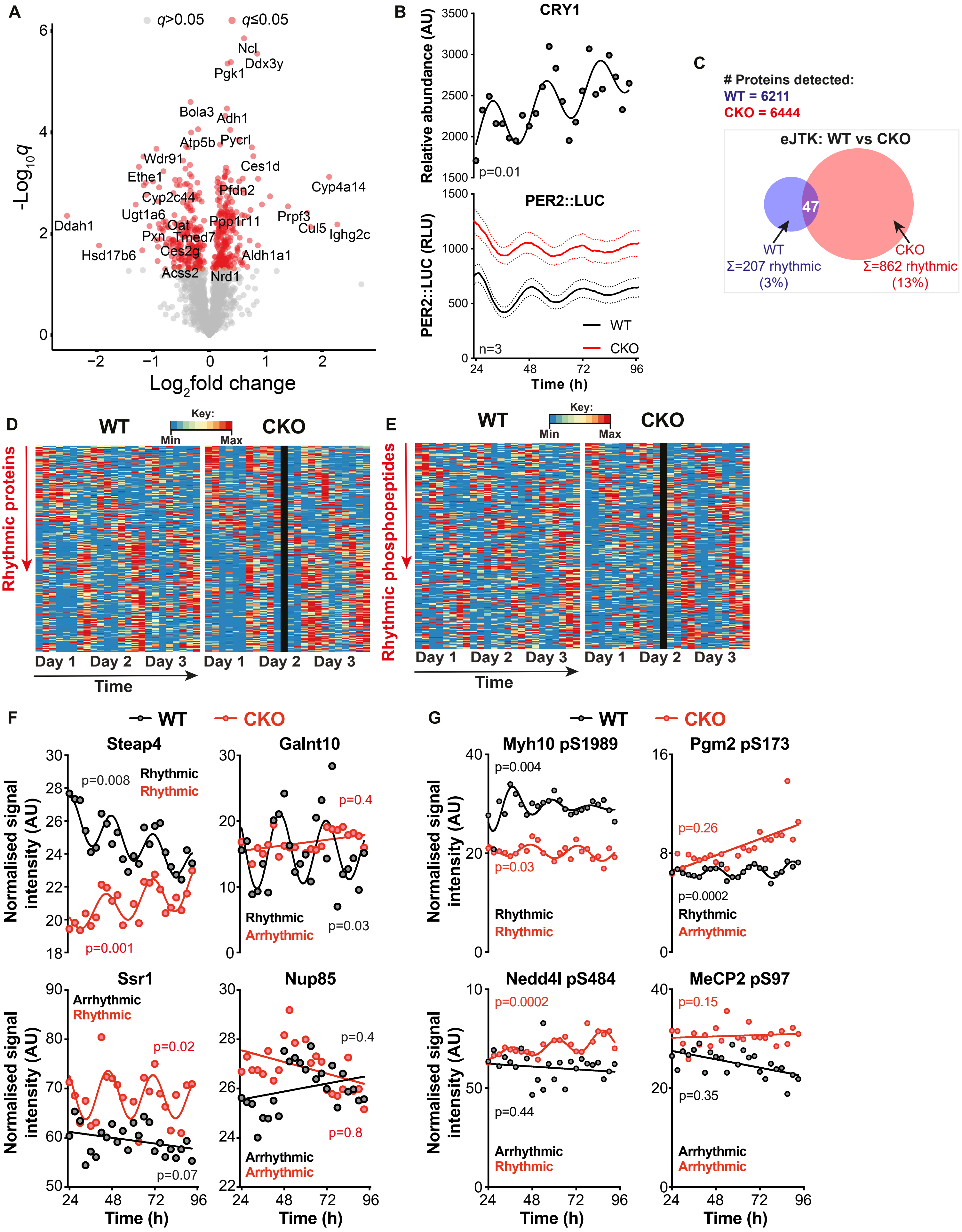

Appendix Figure S2

A

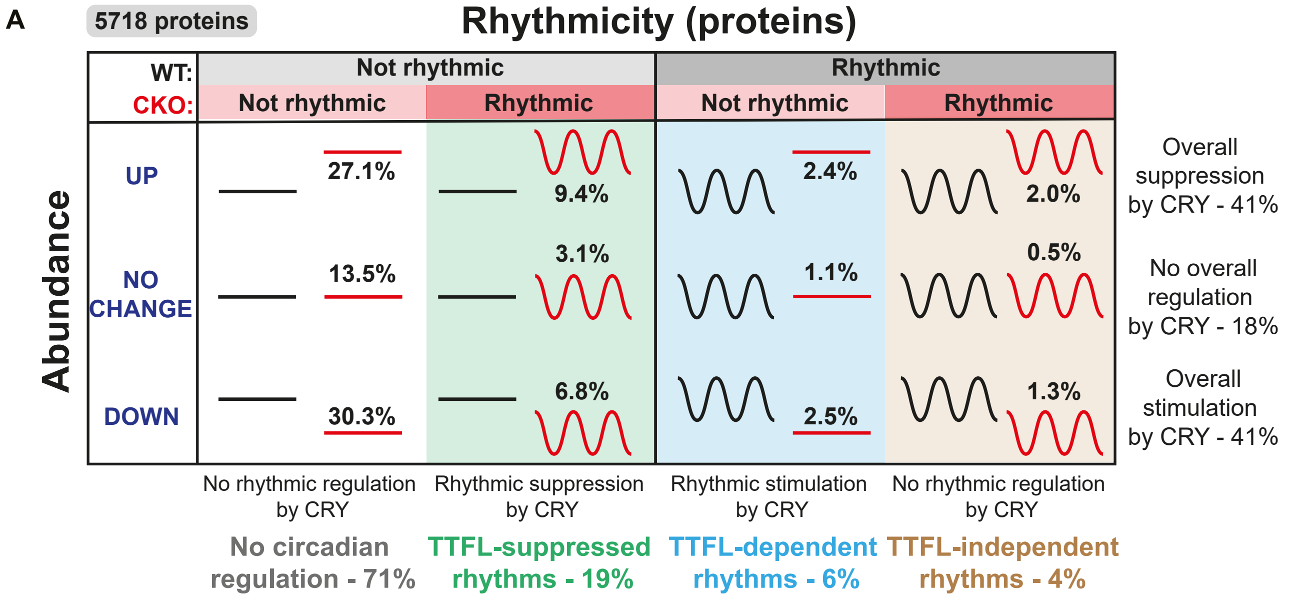

B

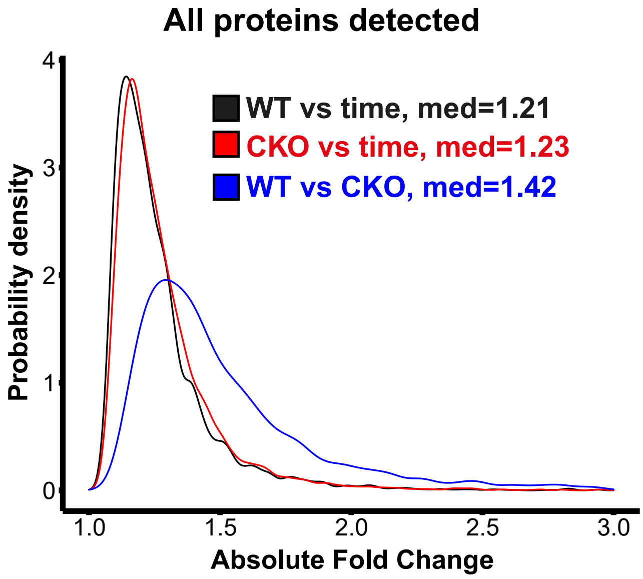

Appendix Figure S3

A

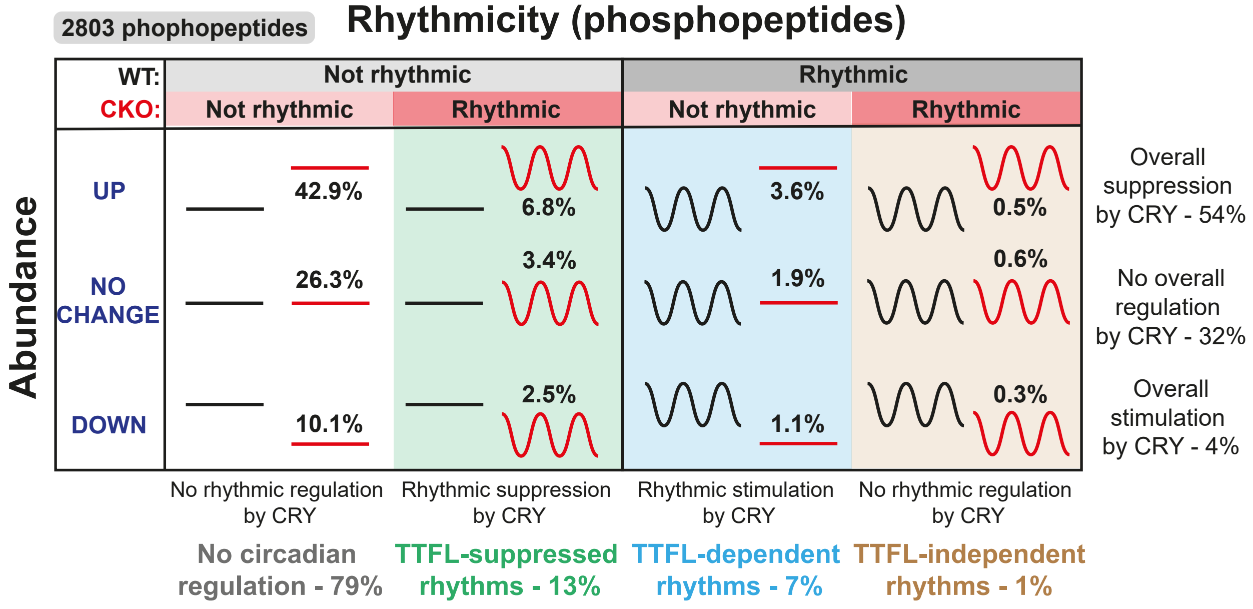

B

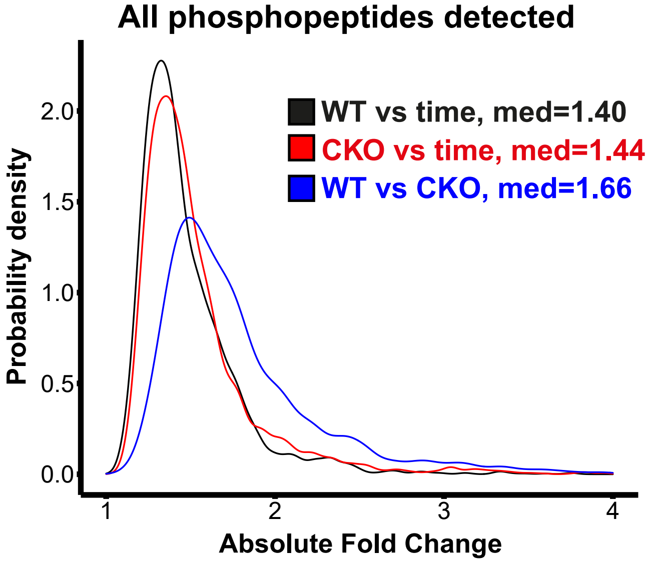

Appendix Figure S4

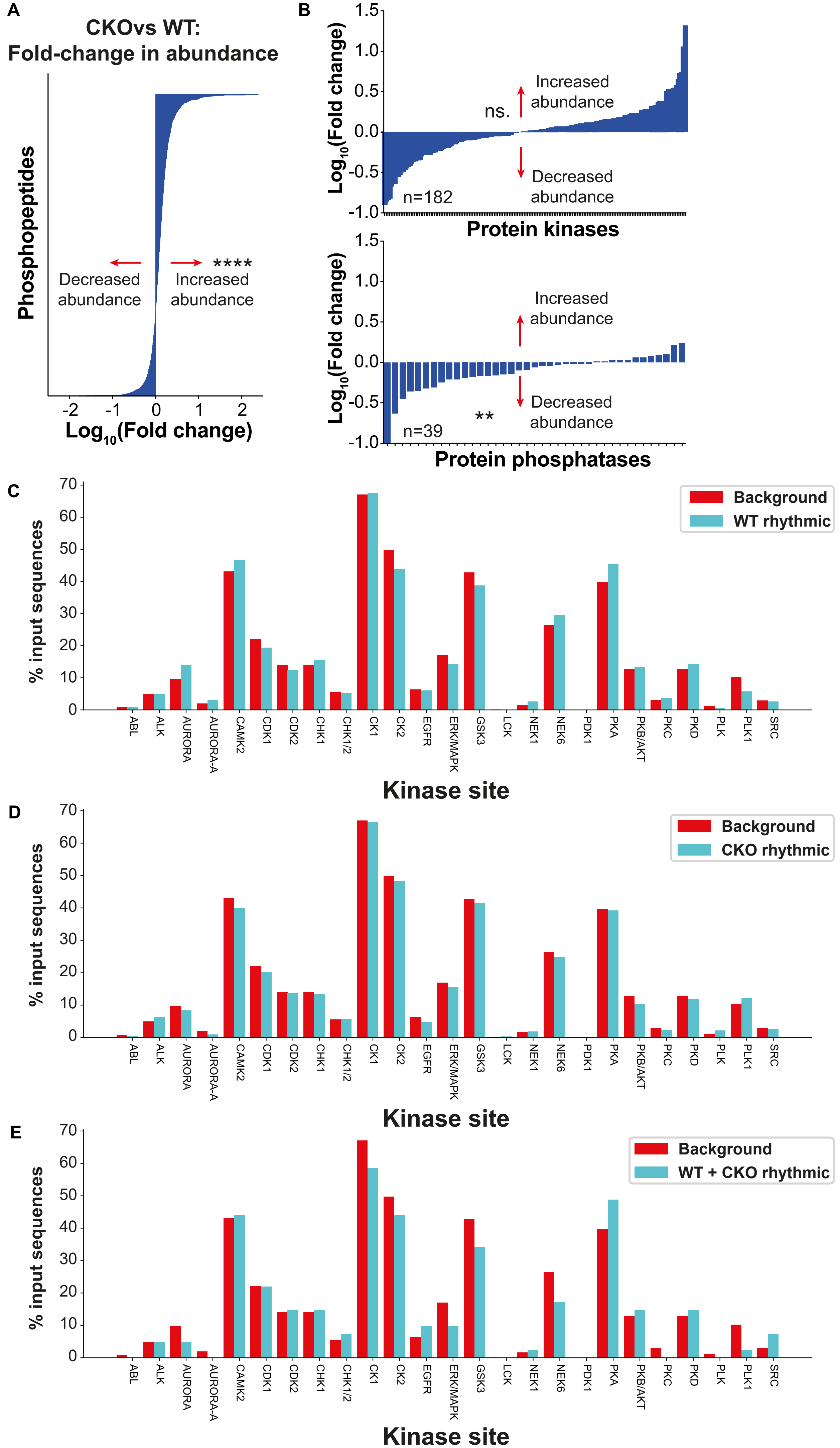

Appendix Figure S5

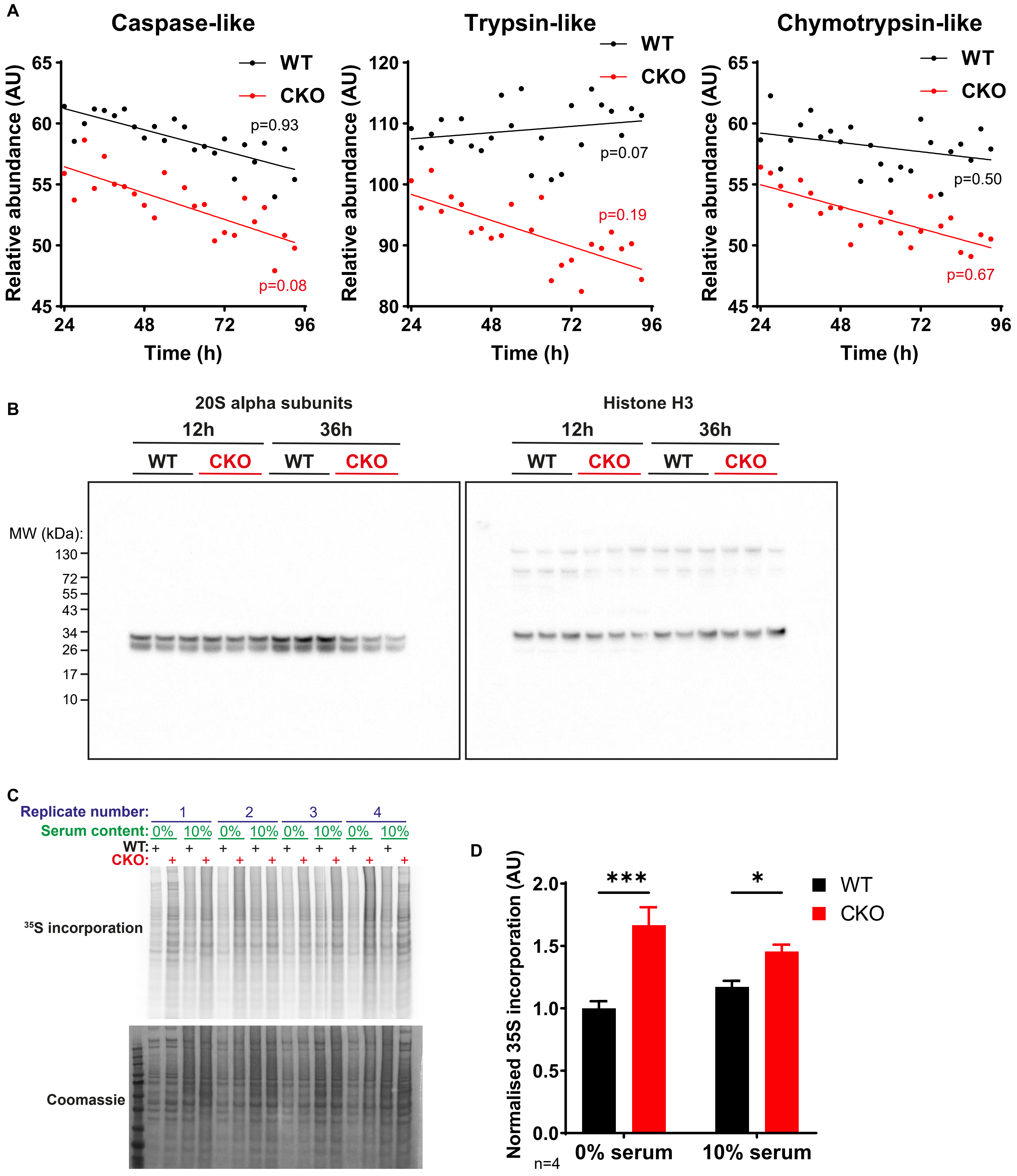

Appendix Figure S6

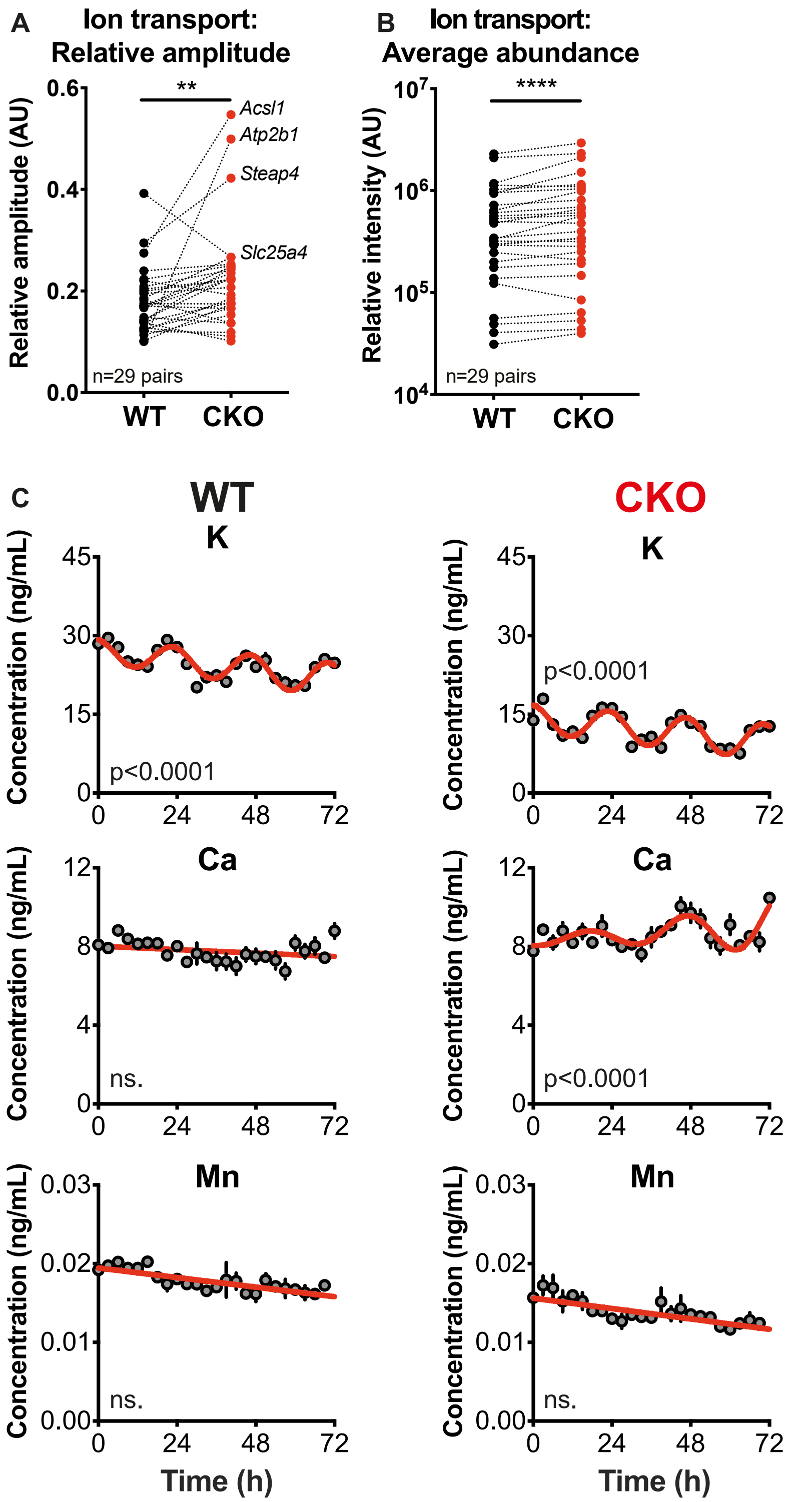

Appendix Figure S7

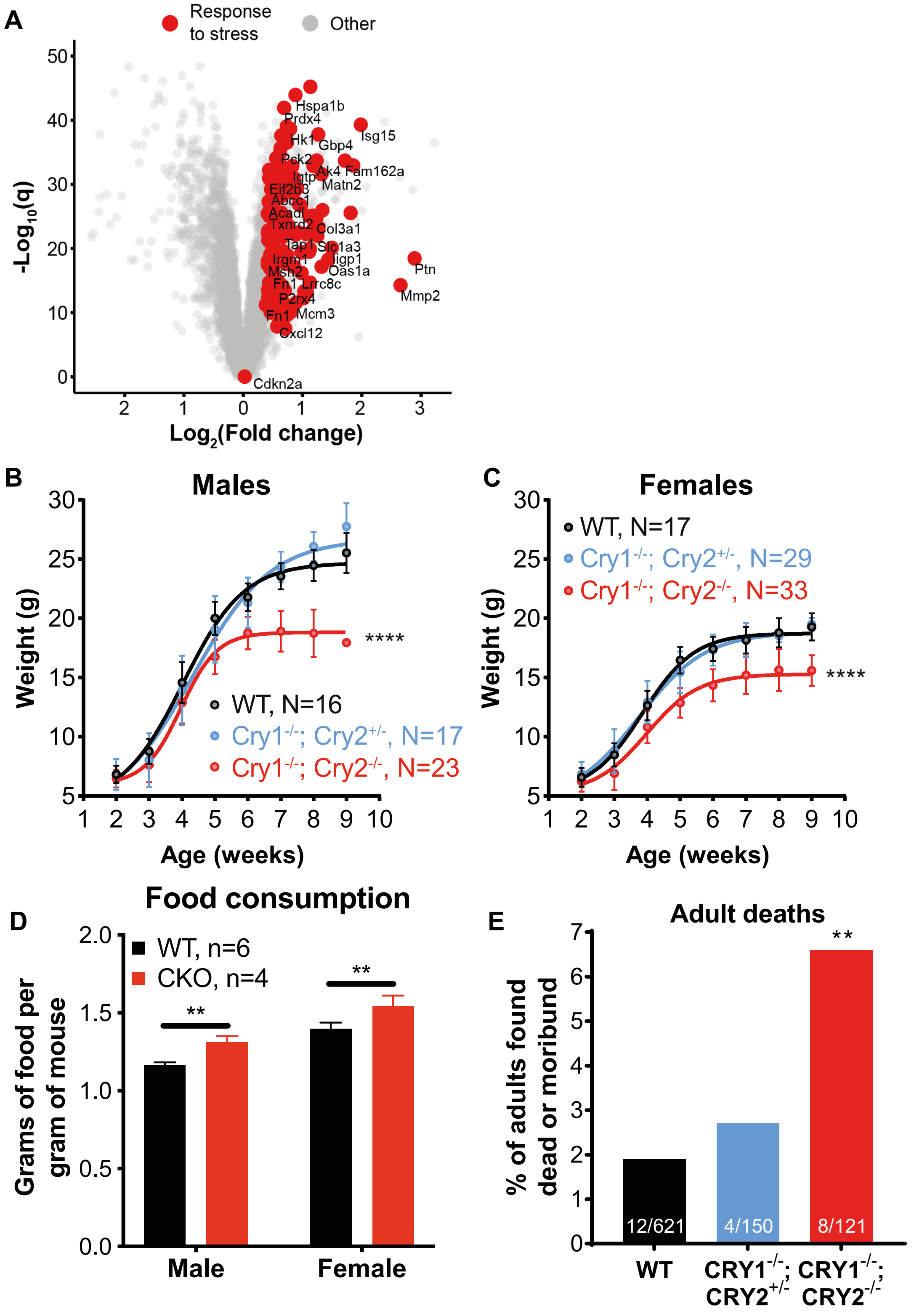

Appendix Figure S8

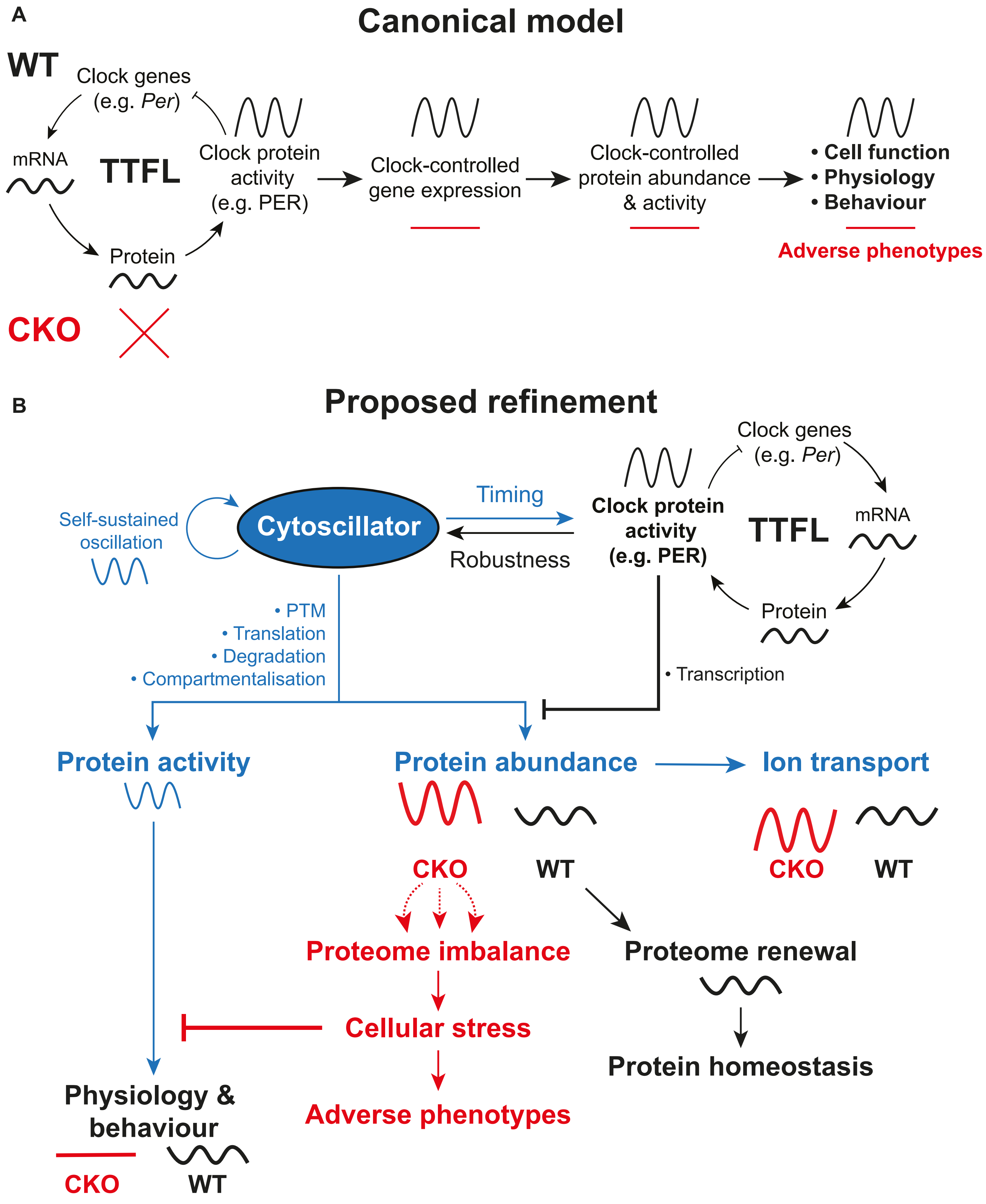

Supplement: Supplementary file 1 — Appendix [file EMBJ-41-e108883-s001.pdf]
