# Supplementary figures and images for: CRYPTOCHROMES promote daily protein homeostasis
Source: EMBO J. 2021 Nov 29;41(1):e108883. doi: 10.15252/embj.2021108883 (PMC8724739; doi:10.15252/embj.2021108883)

Relating to Figure 6A

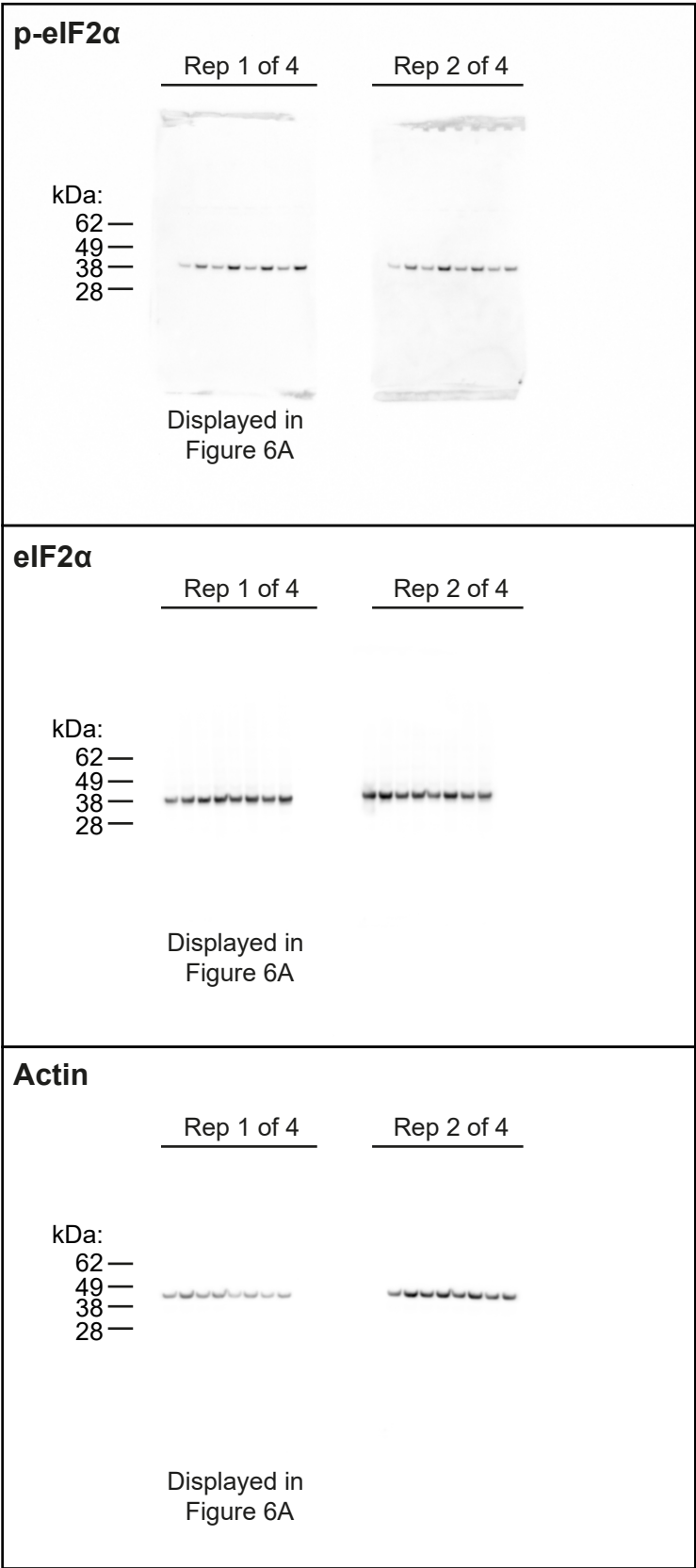

Relating to Figure 6C

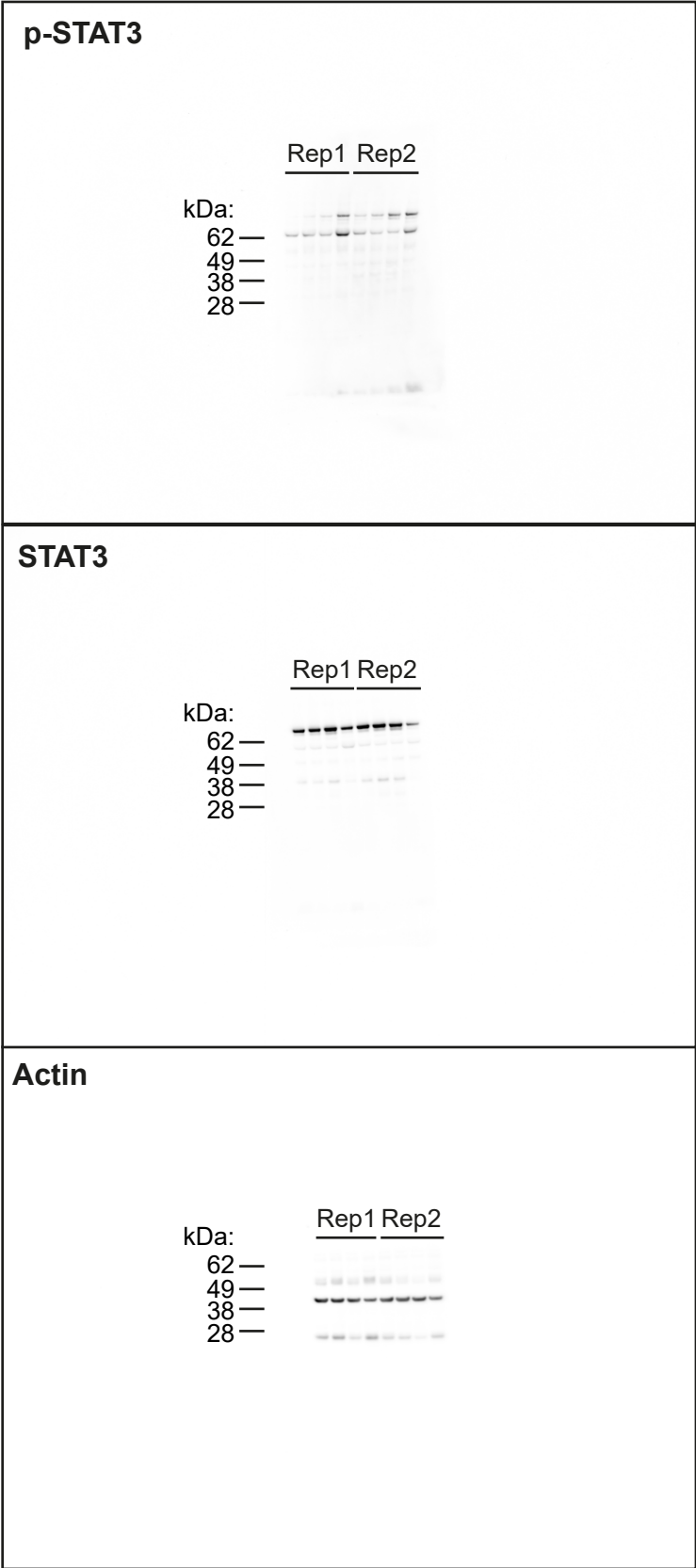

Supplement: Supplementary file 2 — Source Data for Figure 6 [file EMBJ-41-e108883-s003.pdf]
