# Supplementary material for: CRYPTOCHROMES promote daily protein homeostasis
Source: EMBO J. 2021 Nov 29;41(1):e108883. doi: 10.15252/embj.2021108883 (PMC8724739; doi:10.15252/embj.2021108883)
Supplement: Supplementary file 3 [file EMBJ-41-e108883-s002.pdf]

THE EMBO JOURNAL

CRYPTOCHROMES promote daily protein homeostasis

David Wong, Estere Seinkmane, Aiwei Zeng, Alessandra Stangherlin, Nina Rzechorzek, Andrew Beale, Jason Day, Martin Reed, Sew-Yeu Peak-Chew, Christine Styles, Rachel Edgar, Marrit Putker, and John O'Neill
DOI: 10.15252/embj.2021108883

Corresponding author: John O'Neill (oneillj@mrc-lmb.cam.ac.uk)

Review Timeline:

Transfer from Review Commons:	3rd Jun 21
Editorial Decision:	22nd Jun 21
Revision Received:	6th Oct 21
Editorial Decision:	3rd Nov 21
Revision Received:	7th Nov 21
Accepted:	9th Nov 21

Editors: Ieva Gailite / David del Alamo

Transaction Report:

This manuscript was transferred to The EMBO Journal following peer review at Review Commons.

(Note: With the exception of the correction of typographical or spelling errors that could be a source of ambiguity, letters and reports are not edited. Depending on transfer agreements, referee reports obtained elsewhere may or may not be included in this compilation. Referee reports are anonymous unless the Referee chooses to sign their reports.)

Review #1

1. How much time do you estimate the authors will need to complete the suggested revisions:

Estimated time to Complete Revisions (Required)

(Decision Recommendation)

Between 1 and 3 months

2. Evidence, reproducibility and clarity:

Evidence, reproducibility and clarity (Required)

The work presents some interesting theories on the role of CRY in the clock. In general, these theories seem sound based on the data that the authors present. However, a direct link between proteotoxic stress and CRY is not validated, as the authors are not able to alleviate the proteotoxic stress and return the clock to a robust oscillation. In addition, as the authors discuss, these cells were drawn from a single mouse and only a single sample was analyzed at a given time point, giving little statical power to the changes noted in the analysis. Overall, I believe it is important for the authors to make clear that their findings are highly correlated with their predictions but have not been able to demonstrate causation. As there could be many things that cause the proteotoxic stress associated with CRY KOs directly rather than indirectly, it is important to suggest that the CRY KO phenotype could also be an unintentional byproduct rather than an evolved function in the clock. And while I agree that this could support the authors hypothesis that there is another, underlying, clock beyond the TTFL, there are many other possibilities that explain these results, and this should be noted as well.
In summary, I found this work to be interesting and present some novel ideas about the roles of CRY in the TTFL. However, the authors could do more statistically to support their assertions and in some cases either need to specifically state that their findings are correlative and not causative or need to show more direct evidence to support their idea of causation. In addition to these general comments, I have some specific issues with the manuscript (see below).

Major comments

1) The authors state in their introduction that the 10-20% daily variation in protein levels would likely not elicit rhythms in protein function but then go on to argue a great increase in protein oscillations in the CRY KOs with a cutoff of 10% variation. If it is the case that oscillations in these levels don't matter, then the fact that CRY suppresses a 10% variation in protein levels should have no bearing on physiological functions. I do not think that they mean to imply this, and they should revise the comment so that this statement is clearer in the context of the paper. This is also true in reference to Fig 1B. Either 20% abundance change matters, or it doesn't.

2) It would be helpful to have the luciferase traces of the CKO cells, to support their assertion that CRY is contributing robustness to the TTFL in these cells.

3) From the text, it is not clear if biological replicates were run for the proteomics or if all samples were combined and run as one. This should be clear as it has implications as to the confidence in the changes that were seen between the WT and CKO cells.

4) If CRY is suppressing oscillations, it is possible that the rhythms in WT cells would decrease in amplitude over time and not be detected by eJTK. Metacycle or the PAICE suite are better are detecting these non-uniform oscillations and might give you are better idea of what is oscillating and what is not. The same is true for phase calls, neither RAIN nor eJTK is good at calling phase and is 2-4 hours off in most cases.

5) The authors state that "the TTFL-independent circadian rhythm seen in CKO cells drives oscillations of higher relative amplitude and acts preferentially towards more abundant proteins and phosphopeptides" While I do see a correlation between the increased abundance and the oscillations, I do not see evidence that there is a preferential and direct drive in the oscillation higher abundance proteins. This statement either needs to be supported with more direct evidence or needs to be changed.

6) While a P value might validate that these variations in protein abundance are "significantly different" in figure S2, a Cohen statistic will likely show that the change in variability is not meaningful. The authors should run a Cohen analysis to demonstrate that the small changes spread out over many proteins truly represent a meaningful difference.

7) The authors state that there is a decrease in 20S alpha subunits. However, the supplement shows that at 12 hrs the 20S subunits are the same in the WT and the KO but different at 36 hrs. Though the authors state that the lack of differences at 12 hrs are likely due to the medium change, it could also be an induction due to the stress response in cells or it might be that their 36hr tpt was not accurate. More time points are needed to validate that the increase in 20S in WT (or decrease in CRY KO) is valid. In addition, there could be oscillations in the 20S subunits. This data needs to have more timepoints assessed before the authors draw conclusions about the role of CRY in the 20S proteosome.

8) The authors relate proteotoxic stress in the CRY KO to the lack of robustness in the clock. To show this, they added proteotoxic stress elicitors to WT cells and noted that the clock was damped. While this data is correlated, relieving proteotoxic stress in a CRY KO strain and seeing a return to robust oscillations would be the only way to demonstrate that the buffering of proteotoxic stress was the key role of CRY in the clock.

Minor Comments

1) Much of the results reads like a discussion section and the discussion section repeats what is in the results. Consolidating and reorganizing these two sections will streamline

the paper and make the conclusions much more obvious.

2) line 140 should be figure s1b.

3) line 401 synthesise is synthesize

3. Significance:

Significance (Required)

The manuscript "CRYPTOCHROME suppresses the circadian proteome and promotes protein homeostasis" investigates the role that CRY plays in generating circadian rhythms, an important question as the essentiality of CRY in the clock for robustness has been established but the mechanistic role that CRY plays is much less clear. The authors find that a CRY KO leads to global changes in the proteome and phosphoproteomes. They also note that there is an increase in the number of oscillating proteins in the CRY KO, suggesting that CRY is a suppressor of oscillations. The authors also note that the changes in protein levels and oscillating proteins lead to the dysregulation of cellular stress responses. These results lead them to conclude that CRY KO phenotypes are less related to the dysfunction of the clock then they are to CRY specific-clock independent functions and that the main role of CRY in generating circadian robustness is in regulating proteotoxic stress. This work is of interest to most researchers who are interested in circadian rhythms. My expertise is in the fundamental regulation of circadian timing and output and nothing in this manuscript was beyond my expertise.

Review #2

1. How much time do you estimate the authors will need to complete the suggested revisions:

Estimated time to Complete Revisions (Required)

(Decision Recommendation)

Cannot tell / Not applicable

2. Evidence, reproducibility and clarity:

Evidence, reproducibility and clarity (Required)

Please find below the review, which I structured according to the guidelines given by Review Commons.
This review was compiled by David Gatfield, University of Lausanne.

SECTION A - Evidence, reproducibility and clarity

Summary: Provide a short summary of the findings and key conclusions (including methodology and model system(s) where appropriate).

The manuscript reports on how the circadian rhythmic proteomes and phosphoproteomes in mouse fibroblasts are affected by the knockout of the Cryptochrome genes (Cry1 and Cry2), encoding critical inhibitors of the transcriptional feedback loops that drive the molecular circadian oscillator in mammals. A large body of evidence from many labs has previously shown that mice lacking both Cry genes (CKO) are behaviourally arrhythmic - they are, in fact, one of the most frequently used genetic models for a clock-less animal. However, a very recent paper (Putker, [...], O'Neill, 2021, EMBO J 40(7):e106745) - from the same lab that is also submitting the current manuscript - has shown that under specific conditions (e.g. after entrainment through stronger environmental Zeitgeber) circadian rhythms are observable in CKO mice. In the previous paper, the authors put forward their idea that whereas circadian transcriptional feedback imparts robustness and functionality onto biological clocks, the core timekeeping mechanism is post-translational.

In the current manuscript, the authors continue along this line of research. They temperature-entrain CKO fibroblasts and control cells, release them into constant conditions, and sample them over 3 days (resolution 3 hours). In both cell lines, quantitative proteomics allowed the detection of thousands of individual proteins and phosphopeptides. The abundance of most proteins (82% of all detectable) and phosphopeptdes (68%) was significantly different between the two cell lines when averaging over all timepoints of the series. Moreover, when applying rhythmicity detection algorithms to the time series, almost 3-fold more rhythmically accumulating proteins were found in the CKO than in the WT cells, with only few rhythmic proteins in common. Of note, for the main rhythmicity analyses, relatively lenient rhythmicity criteria were applied (relative amplitude threshold of 10%; main detection algorithm RAIN; however, also the more stringent JTK method resulted in similar results). The ensemble of these findings - all in Figure 1 - are unexpected and intriguing given that normally less rhythmicity, rather than more rhythmicity, would be expected in cells in which the Cry1/2 genes are inactivated. Further analyses of the proteomics data, shown in Figure 2, explore more detailed rhythmic properties of the oscillating proteins. Proteins rhythmic in both cell lines have a moderately higher amplitude and are on average more abundant in CKO than in WT. Moreover, depending on which subsets of rhythmic events were analysed and compared (i.e. proteins, phosphoproteins, common, or exclusive to one of the two genotypes), phase distributions of the oscillating events were differentially affected. The intermediary conclusion that the authors make is that the role of CRY proteins is to suppress rhythmic events (proteins/phosphoproteins), which now, in the CKO cells, are unmasked and detectable.

In the rest of the manuscript the authors put together different ideas for the underlying mechanism(s) and show data that is globally consistent with these ideas. First, they hypothesise that in order to accumulate higher average (rhythmic) protein levels, proteasomal activity may be reduced and translation rate increased. Figure 3 shows data that indeed points in this direction, especially for the lower proteasomal activity. Another difference between CKO and WT cells, shown in Figure 4, concerns the overall

cytosolic protein content and - possibly coupled to this - the regulation of osmotic homeostasis, both of which show genotype-dependent differences in their daily patterns. These findings are consistent with the idea that in the absence of CRY proteins some sort of "proteomic imbalance" occurs - a state that may be reflected in higher sensitivity to proteotoxic stress; indeed, different assays shown in Figure 5 indicate that CKO cells have higher basic and inducible (by tunicamycin) levels of eIF2alpha phosphorylation (marker of integrated stress response), and CKO mice are sensitive to cellular stress induction as well (phospho-STAT3 levels after proteasome inhibitor treatment). Finally, in Figure 6, the authors induce proteotoxic stress conditions similar to what CKO cells experience under basic levels, now in WT cells. The observation is that under conditions of chronic stress (using 3 different inducers), rhythmicity of the circadian clock is reduced/abolished in WT cells, as judged from circadian reporter rhythms (transcriptionally driven PER2::LUC reporter). Overall, the authors conclude that CRY proteins have an important role in buffering overall protein biosynthesis against (circadian) fluctuations.

Major comments:

- Are the key conclusions convincing?

(1) Concerning the initial findings of more abundant rhythmicity in the CKO vs. WT cells (i.e. Figures 1-2), the conclusions are convincing within the limits of the applied experimental design. Thus, I am concerned that the data originate (if I understand this correctly) from a single cellular CKO and WT preparation (or pool of cells) that has been in culture for up to 30 passages. It is difficult to assess whether lower or higher detectable rhythmicity is a (to be a bit provocative: arbitrary?) feature of the different cellular preparations, or truly of the different genotypes. Many labs have experienced that in different cellular preparations, even when they are isogenic, there can be per se quite substantial differences in cellular morphology, gene expression, and many other parameters, including rhythmicity dynamics. Ideally, the experiment should therefore have included several biological replicates (i.e. independent cell preparations) or even a rescue experiment: re-expressing CRYs in the CKO line. Otherwise, I do not see how the authors can make a strong argument for a genotype-specific effect.

Independently of this issue, instead of the interpretation that CRYs suppress rhythmicity that becomes unmasked in CKO cells, I am wondering whether the authors have also considered an alternative model according to which the difference in rhythmicity detectable under free-run actually lies in differential sensitivity to the temperature-entrainment protocol in CKO vs WT cells? Maybe CKO and WT cells do not differ in their rhythmic proteomes per se, but only in the efficiency with which one can synchronise the cells, which will then be reflected in the protein rhythms during free-run?

(2) The other findings (Figures 3-6) are overall convincing, but again with the caveat of using single CKO and WT preparations for many conclusions. However, this risk is hedged somewhat better here, through additional experiments (e.g. in mice).
Still, I would like to point out that while the findings in Figures 3-6 are generally consistent with the proposed model, there are overall relatively few experiments that actually attempt to challenge it. Much of the data thus remains suggestive and

circumstantial.

I also do not agree with the statement (page 10) that it is not necessary to interrogate the transcriptome in the CKO cells because this has been done previously. It would be important to know which of the rhythmicity changes (or, as a matter of fact, gene expression changes at large) show parallel transcriptomal effects as well, and which ones are truly translationally/post-translationally driven. I do not believe that the reference to historical controls is valid in this case - for an integrative view of the sources of changes in protein levels, it would be essential to know how the mRNA levels change within the same samples.

- Should the authors qualify some of their claims as preliminary or speculative, or remove them altogether?

I would say that overall probably a number of claims could require considerable toning down - when reading the results part, several times I thought this would be better for discussion.
I am not listing these points here in detail; further down in this review, I give a short compilation of some things I noticed while reading the manuscript.

- Would additional experiments be essential to support the claims of the paper? Request additional experiments only where necessary for the paper as it is, and do not ask authors to open new lines of experimentation.

The lack of true biological replicates (or rescue experiments) in the mass-spec data is problematic in my view.
I am not sure whether there is a less laborious (time & cost) way around doing such an experiment, but I am reluctant to accept that one can make quite substantial novel claims on CRY proteins based on a single cell preparation/cell line.

- Are the suggested experiments realistic in terms of time and resources? It would help if you could add an estimated cost and time investment for substantial experiments.

This is something I cannot judge.
But quality of the data and validity of the interpretation is for me the only thing that is relevant here.

- Are the data and the methods presented in such a way that they can be reproduced?

Yes, I think this is overall well described.

- Are the experiments adequately replicated and statistical analysis adequate?

See above. This is where I see the main issue with the study in its present form!

Minor comments:
- Specific experimental issues that are easily addressable.

I have a list of individual comments just below.

- Are prior studies referenced appropriately?

Yes.

- Are the text and figures clear and accurate?

Overall yes.

- Do you have suggestions that would help the authors improve the presentation of their data and conclusions?

In the following I list a couple of points that the authors may find helpful and that are generally easy to correct:

-Page 4, line 74: "Similarly, circadian oscillations persist in cells and tissue slices lacking BMAL1 (37,38)" Given the problematic situation surrounding the paper Ref. 37 (Ray et al., 2020), the authors are probably better advised to not use it as an argument to support their story.

-Page 6, line 109-110: "In our cellular time course, as expected, Cry1 was selectively detected in WT, but not in CKO cells, and [...] (Figure S1B).". It would be good to show all core clock protein data (where detectable) in WT vs CKO, as supplemental material.

-Figure S1D-G: In the heatmaps (S1D-E), a black line indicates the loss of a specific timepoint from the time series (explained in figure legend). However, individual protein plots in S1F-G appear to show all timepoints. How is this possible?

-Page 7, line 129: "In this way we found that more abundant proteins were more likely to be rhythmic than less abundant proteins, but crucially, [...]". Can this simply by an effect of signal/noise, variation, statistical power, etc.??

-Page 7, line 140: Reference to (Figure S1A) should be corrected to (Figure S1B).

-Page 8, line 158: "Secondly, in WT cells, CRY regulates the phase of rhythmicity for a subpopulation of proteins and phosphopeptides whose abundance peaks around the same phase as CRY1 and in antiphase with the majority of the rhythmic (phosphor)proteome." I am not sure this claim can be made here from the presented data, because the phase difference of the commonly rhythmic proteins is not shown?

Page 9 (line 186) - page 10 (line 209): all this is very speculative and hypothetical - rather something for discussion than for results section?

Page 10, lines 212-217. As already mentioned above, the fact that other publications have studied CKO transcriptional changes in other biological samples, paradigms etc., does not mean that the authors can ignore them in this study with, after all, other cells, other entrainment paradigms, etc.!
The authors are taking a shortcut that I find difficult to accept - transcriptomal changes should be taken into account in order to be sure what effects are really occurring at the

protein level! Difficult to look at the protein in isolation.

Page 13, line 286: "This may be reconciled by our observation that density regulated protein (DENR) and eIF2A. additional alternative subunits of the translation initiation complex. Were significantly upregulated in CKO cells compared with WT (Figure S6B-C);". The increase in DENR is so minimal, it is hard to imagine that this can explain anything. Honestly, I do not see the sense in this statement, it's unnecessary distraction and not hard data. In general, there are too many such suggestive claims in the results section that are then not followed up on.
BTW, DENR likely has very little, if any, role in canonical translation (but rather in re-initiation).

Figure 3C vs. 3B: The quantification of the Western blot does not appear to reflect the experiment itself. On the membrane (3B), it does not look like a reduction down to <25 % that is show in the quanfication (3C); also the (supposedly more quantitative) mass-spec data suggests that the effect size is smaller. Did something go wrong in the quantification?

Figure 3D: The signal in this assay depends on cell number or "total cytoplasmic content per well." In the Methods section, I saw that 20'000 cells were seeded per well. Question: at the time of experiment, were cell numbers equivalent (e.g. are proliferation rates - from seeding to measurement - of CKO vs. WT the same?), and are the cells actually of comparable size (i.e. do 20'000 CKO cells have about the same cytoplasmic equivalent as 20'000 WT cells)? The assay could be, for example, normalised to total protein content.

Figure 3E: The significance is indicated with (**), but the same graph in Figure S4C only carries one star for significance (*).

Figure 3: How do low proteasome activity (=more stable proteins) and stronger protein amplitudes fit together? This is something I did not fully understand.

Figure 5A: Are p-eIF2alpha peptides detected in the original mass spec data? If yes, can the quantification be added to this Figure?

Figure 5C: the WT animals are rhythmic, but the CKOs are not. Is this a concern (confounding factor) in this experiment?

3. Significance:

Significance (Required)

SECTION B - Significance

- Describe the nature and significance of the advance (e.g. conceptual, technical, clinical) for the field.

The nature and significance of the advance would be "conceptual", representing a new way of how we may think about the role of CRY proteins for gene expression - inside

and outside of the clock!

- Place the work in the context of the existing literature (provide references, where appropriate).

From the original view - held until a few years ago - that CRY proteins are exclusively active as components of the core clock negative limb, broader functions have been documented over the last years. In particular, work by the Lamia lab and by others has shown a role as a modulator of nuclear receptor-mediated transcription - giving CRYs also a role in output pathways. In this context, further investigations into other/additional roles of CRYs are important and welcome.

- State what audience might be interested in and influenced by the reported findings.

I think that (for sure in the current state in which the authors do not provide a molecular explanation of how CRYs supposedly regulate the various proposed processes - from proteasome regulation, to translation etc.) this work would be mainly interesting for the broader circadian clock community.

- Define your field of expertise with a few keywords to help the authors contextualize your point of view. Indicate if there are any parts of the paper that you do not have sufficient expertise to evaluate.

My expertise: Circadian clocks, RNA biology/translation, gene expression more broadly.

We thank both reviewers for their most constructive comments. Below we respond to each of their comments in detail. We have also now included additional experimental evidence, in cells and mice, to address their comments and hope that they will both find the manuscript to be much improved.

We note that both reviewers were concerned by a single point on which we were quite clear in our original submission: that a single well-characterised cell line was used to test the hypothesis that CRY is essential for circadian regulation of the mammalian cellular proteome. Our data are evidently sufficient to refute this hypothesis – it cannot be true if a single exception is observed. We agree that the increased number and amplitude of rhythmic proteins detected in CKO vs WT cells would not be sufficient to support our secondary finding that CRY functions to suppress circadian protein rhythms – if considered in isolation. For this reason, we interrogated *in vivo* data from multiple biological replicates that were collected and previously published by an independent lab (Fig S2C). We hope the reviewers agree that independent physiological validation *in vivo* should automatically render additional cellular experiments to be redundant, since the cell is a simplified model for the mouse.

Therefore, whilst we are not at all resistant to doing further proteomics experiments, we do not see how their results could have any bearing on the current manuscript. We have already established that the expression of circadian rhythms in CKO cells is more variable than in WT cells (Putker *et al*, 2021); thus, were we to repeat the entire cellular proteomics experiment with an independent cell line and observed a smaller effect size, it would not change that fact that CRY is not essential for circadian regulation of the proteome in mammalian cells because this was already observed in the first set of experiments (Fig 1, 2, 4D). Moreover, it would not change the observation that CRY effectively functions to suppress temporal variation in protein abundance, since we have already validated this prediction in mouse tissue, i.e., no cellular data can render the mouse tissue result to be any less true.

Whilst outside the scope of our original study, we do agree that it might be interesting to assess the extent of within-genotype variation, comparing independently generated WT and CRY lines. However, we would like to emphasise that this cannot change the observations we have already made, nor their interpretation, for the reasons outlined above. We would therefore appreciate some guidance here. Multiplexed isobaric tag mass spectrometry has advanced since our first experiment and can now accommodate 16 samples in a single mass spectrometry run. We would therefore propose to compare 4 independent cell lines (2 male, 2 female) from each genotype, at two circadian timepoints (36 and 48h). Based on our previous findings, we would expect more variation in protein abundance between CKO lines, and more variation between timepoints within each CKO line, compared with wild type controls. We have hesitated to begin this experiment, as we are not confident that this is what the reviewers are looking for, and because it is unlikely to provide more insight than already furnished by the mouse liver analysis.

Reviewer #1 (Evidence, reproducibility and clarity (Required)):

The work presents some interesting theories on the role of CRY in the clock. In general, these theories seem sound based on the data that the authors present. However, a direct link between proteotoxic stress and CRY is not validated, as the authors are not able to alleviate the proteotoxic stress and return the clock to a robust oscillation.

We thank the reviewer for their comments. Unfortunately, there has been a misunderstanding that we claim a direct link between CRY knock-out and proteotoxic stress. We do not claim this, nor is it addressed by our experiments, and we apologise if this was insufficiently clear. A vast literature already connects proteome imbalance with sensitivity to proteotoxic stress, and is taken as

foundational knowledge for our investigation (Hipp *et al*, 2019; Harper & Bennett, 2016). CRY-deficient mice and cells differ from wild type controls in many ways, presumably because CRY proteins directly regulate several different biological functions (Huber *et al*, 2016; Correia *et al*, 2019; Koike *et al*, 2012; Lamia *et al*, 2011) and indirectly regulate many more (Kriebs *et al*, 2017; Koike *et al*, 2012). We found that overall proteome composition (82% detected proteins) of CRY-deficient cells was significantly different compared with wild type i.e. the proteome is imbalanced with respect to wild type controls, and CRY-deficient cells and tissues are more sensitive to proteotoxic stress. We do not propose any specific mechanism since proteotoxic stress is known to be multifactorial and can occur through many different mechanisms (Anckar & Sistonen, 2011; Hetz & Saxena, 2017; Sasaki & Hiderou, 2015; Zhao *et al*, 2002; Pakos-Zebrucka *et al*, 2016). Rather, we observe that proteome imbalance and increased propensity towards proteotoxic stress is associated with CRY-deficiency and propose that proteotoxic stress underlies and contributes to other CRY-deficient phenotypes that have hitherto been attributed solely to the absence of CRY-mediated cycling clock gene regulation (Bur *et al*, 2009; Masuki *et al*, 2005; Barclay *et al*, 2013; Takasu *et al*, 2015). We have revised Figure 6G and Figure S7B along with accompanying figure legends to emphasise the indirect nature of the association between CRY deficiency and proteome imbalance, and altered the abstract and discussion as follows:

Line 22: *"Proteome imbalance and increased temporal variation of protein abundance in CRY-deficient cells and tissue was linked with increased proteotoxic stress, which impairs circadian robustness, and may contribute to the wide-ranging phenotypes of CRY-deficient mice."*

Line 358: *"Our observations in vitro and in vivo are more consistent with a model whereby CRY functions indirectly to prevent proteome imbalance by suppressing temporal variation in protein abundance."*

We agree with the reviewer's prediction that reduction of proteotoxic stress should increase the robustness of PER2::LUC rhythms in CRY-deficient cells. Importantly, however, we would not expect a complete rescue of circadian robustness since these cells would still lack any underlying transcriptional cycles that are proposed to amplify and confer hysteresis upon daily rhythms in PERIOD protein activity (Putker *et al*, 2021; Wong & O'Neill, 2018). To test the reviewer's prediction, we treated cells with a low (sub-saturating) concentration of cycloheximide in order to reduce the overall rate of protein synthesis, and thereby reduce the increased burden placed on the protein quality control machinery in CKO cells (Kim & Strange, 2013; Peng *et al*, 2015; Parzych *et al*, 2015). Consistent with prediction, we observed that CKO PER2::LUC rhythms became more robust (rhythms persisted for more cycles) under mild inhibition of protein synthesis, but importantly did not rescue them to wild type level. These data are now presented in revised Figure 6, with the following changes to the manuscript text:

Line 325: *"Conversely, the relief of proteotoxic stress, by partial inhibition of protein synthesis with sub-saturating cycloheximide (CHX) (Kim & Strange, 2013; Peng et al, 2015; Parzych et al, 2015), was sufficient to partially rescue PER2::LUC rhythms in CKO cells (Figure 6G, H). Critically, the relative amplitude of CHX-rescued PER2::LUC rhythms was much lower than wild type PER2::LUC rhythms, commensurate with the established contribution of CRY proteins in the daily repression of Period gene expression, which is absent from CKO cells. This is consistent with our hypothesis (Putker et al., 2021) that CRY-mediated transcriptional feedback amplifies a post-translational circadian timing mechanism regulating PER protein stability/activity, that is rendered less robust and frequently masked by proteotoxic stress associated with CRY-deficiency."*

In addition, as the authors discuss, these cells were drawn from a single mouse and only a single sample was analyzed at a given time point, giving little statical power to the changes noted in the analysis.

In our previous work we demonstrated the variable expression of CRY-independent circadian PER2::LUC rhythms in multiple independent lines (Putker *et al*, 2021). In this investigation we specifically addressed the function of CRY in the temporal regulation of a cellular proteome. Sampling with high temporal resolution over several independent circadian cycles provides greater statistical power to detect cycling abundance over time than sampling with many technical replicates and lower resolution (Hughes *et al*, 2017). Compared with the temporal regulation of a cellular proteome, the use of cells from multiple mice would provide insight into biological variation within each genetic background. This was not a focus of our investigation, but in light of other reviewer comments (described and discussed above), we agree that some exploration of the biological variation within each genotype would have been helpful if we had not also validated key findings in mouse tissue *in vivo*. As discussed on page 1, the outcome of further cellular mass spectrometry experiments cannot affect the two main findings: that CRY is not essential for circadian proteome regulation (demonstrated in Fig 1, 2, 4) or that CRY suppresses temporal proteome variation (validated in mouse tissue, Fig S2C).

Overall, I believe it is important for the authors to make clear that their findings are highly correlated with their predictions but have not been able to demonstrate causation. As there could be many things that cause the proteotoxic stress associated with CRY KOs directly rather than indirectly, it is important to suggest that the CRY KO phenotype could also be an unintentional byproduct rather than an evolved function in the clock. And while I agree that this could support the authors hypothesis that there is another, underlying, clock beyond the TTFL, there are many other possibilities that explain these results, and this should be noted as well.

In summary, I found this work to be interesting and present some novel ideas about the roles of CRY in the TTFL. However, the authors could do more statistically to support their assertions and in some cases either need to specifically state that their findings are correlative and not causative or need to show more direct evidence to support their idea of causation. In addition to these general comments, I have some specific issues with the manuscript (see below).

We thank the reviewer for these comments, we address specific statistics comments below.

Regarding correlation vs causation, we absolutely agree that current understanding of the circadian function of CRY is largely correlational. To reiterate, although plausible and widely assumed, there is no direct evidence that a TTFL involving the Cry genes/proteins directly generates clock-controlled protein abundance rhythms in mammalian cells – even PER2 protein (not mRNA) rhythms persist in CRY-deficient cells, indicating a signal amplification not generation function for CRY (Putker *et al*, 2021). Moreover, several other experimental observations from independent labs are not consistent with CRY oscillations fulfilling the function attributed to them in the canonical TTFL model. For example, constitutive expression of CRY does not abolish E-box mediated circadian transcriptional rhythms (Fan *et al*, 2007; Nangle *et al*, 2014); and there is greater temporal protein variation in CRY KO mouse liver than in WT (Fig S2C from Mauvoisin et al. (Mauvoisin *et al*, 2014)). It is not disputed that CRY proteins normally facilitate rhythmic repression of transcription at many genomic loci, nor that CRY proteins must be present within some permissive range for transcriptional rhythms to be observed. On the other hand, whilst a modulatory role has been clearly established, there is simply no direct evidence that CRY-dependent transcriptional cycles are the essential mechanism that actually generates clock-controlled protein rhythms in mammalian cells.

Thus, in this investigation, we specifically and directly test a clear prediction from the prevailing hypothesis, that CRY proteins are essential for circadian regulation of mammalian cellular proteomes (Kume *et al*, 1999; Sato *et al*, 2006; Ye *et al*, 2014). Our findings are incompatible with that hypothesis and so we propose a refinement to it. We do not claim causality, rather we refute a previous claim of causality - the observation of increased circadian protein abundance rhythms in a single CRY-deficient mammalian cell line, validated in mouse liver, is sufficient to refute the hypothesis that CRY is essential for the generation of circadian protein rhythms in mammalian cells. We do not claim that the refinement we propose (Fig S7) is "true", merely that it agrees substantially better with empirical observation than the prevailing paradigm, and makes testable predictions, several of which we validate experimentally. We have revised the text to communicate this point and associated caveats more clearly, as follows:

Line 352: *"In this study we found that circadian regulation of the cellular proteome persists in CRY1$^{-/-}$; CRY2$^{-/-}$ (CKO) mouse fibroblasts under constant conditions. This is consistent with previous studies showing circadian oscillations in cellular reporter activity and whole-organism behaviours in this genetic background (Putker et al, 2021; Maywood et al, 2011; Ono et al, 2013a, 2013b), and is incompatible with the prevailing hypothesis that daily cycles of CRY-mediated transcriptional repression are the essential mechanism that generates circadian regulation of cellular function and organismal physiology (Sato et al, 2006; Kume et al, 1999; Ye et al, 2014). Our observations in vitro and in vivo are more consistent with a model whereby CRY functions indirectly to prevent proteome imbalance by suppressing temporal variation in protein abundance. Validating predictions from this model by several approaches, we found that CRY deficiency was linked with proteotoxic stress, which renders circadian rhythms less robust in cells and in mice. There is no reason to doubt that CRY proteins normally regulate circadian transcription, rather our data suggest that the function of CRY-mediated circadian transcriptional regulation requires urgent reappraisal. In light of our findings, we propose a refinement to current models for the generation and utility of circadian rhythms in mammalian cells (Figure S7). We anticipate that experimental comparison of this refinement with predictions from the canonical TTFL model will help to distinguish the circadian functions of CRY proteins from other possible interpretations."*

****Major comments****

1) The authors state in their introduction that the 10-20% daily variation in protein levels would likely not elicit rhythms in protein function but then go on to argue a great increase in protein oscillations in the CRY KOs with a cutoff of 10% variation. If it is the case that oscillations in these levels don't matter, then the fact that CRY suppresses a 10% variation in protein levels should have no bearing on physiological functions. I do not think that they mean to imply this, and they should revise the comment so that this statement is clearer in the context of the paper. This is also true in reference to Fig 1B. Either 20% abundance change matters, or it doesn't.

We believe the reviewer may have misunderstood our meaning. For most proteins, a 10-20% variation in their overall abundance would not be expected to elicit any change in protein activity simply because the abundance of most enzymes is not rate-limiting for enzyme activity under physiological conditions (Aragón & Sols, 1991; Bulik *et al*, 2016; Rocca *et al*, 2015; Nadaraia *et al*, 2007). We do not claim that the daily rhythms in protein abundance in cells or in vivo have any physiological relevance; rather this has been claimed or implied by others (Cox & Takahashi, 2019; Zhang *et al*, 2014a; Lowrey & Takahashi, 2011; Jang *et al*, 2015; Cederroth *et al*, 2019; Reddy *et al*, 2006; Dunlap, 1999; Lück & Westermark, 2016; Neufeld-Cohen *et al*, 2016). In our analysis of changes in protein abundance, we employed a 10% relative amplitude threshold as a conservative, evidence-based estimate for cell-intrinsic noise of protein expression i.e. a daily abundance rhythms of <10% relative amplitude cannot have biological relevance, since this much variation is observed

between otherwise identical cells in the same culture at the same time (Elowitz *et al*, 2002; Raser & O'Shea, 2004; Volfson *et al*, 2006; Sigal *et al*, 2006; Pedraza & Van Oudenaarden, 2005). This does not mean that protein rhythms >10% can be assumed to have biological relevance, and nor do we do address whether rhythms of any specific protein have any biological importance.

To be clear, the current paradigm for circadian regulation of mammalian cellular physiology assumes that circadian regulation of biological processes is achieved through clock-controlled gene expression resulting in circadian rhythms in the abundance of clock-controlled proteins that leads to a rhythm in the activity of those proteins. Again, there is little direct evidence to support the assumption that physiological protein abundance rhythms result in protein activity rhythms, nor is it really consistent with contemporary understanding of protein homeostasis given the large proportion of cellular energy budgets that are employed to maintain constant protein levels in other contexts (Franks *et al*, 2017; Hipp *et al*, 2019; Wolff *et al*, 2014; Harper & Bennett, 2016; Wang *et al*, 2019; Liu *et al*, 2016a). The alternative hypothesis therefore merits some consideration: that, as in yeast metabolic oscillations, cellular circadian rhythms promote protein homeostasis by temporal consolidation of proteome renewal, whilst minimising energetically costly changes in the abundance of most proteins (O'Neill *et al*, 2020). Again, we do not claim that this "true", merely that it is more consistent with empirical observation than the hypothesis that daily protein rhythms drive daily rhythms in cell function.

To prevent any misunderstanding of this point, we have revised the text as follows:

Line 452: *"The extent of daily variation for most 'clock-controlled proteins' is rather low however (relative amplitude <20%, Figure 1D, Figure S2C), and there is little reason to think that such modest changes in protein abundance would impact on protein function in most cases (Aragón & Sols, 1991; Bulik et al, 2016; Rocca et al, 2015; Nadaraia et al, 2007)."*

2) It would be helpful to have the luciferase traces of the CKO cells, to support their assertion that CRY is contributing robustness to the TTFL in these cells.

This is not an assertion but the focus of our previous paper, which employed many different independent biological replicates and contexts (Putker *et al*, 2021). As the reviewer will be aware, the prevailing paradigm posits that circadian rhythms in mammalian cells are ultimately generated by an auto-regulatory delayed transcriptional feedback loop in which CRY genes/proteins are essential components. In CRY-deficient cells, we found no evidence for continued TTFL function (consistent with predictions of the TTFL model), but found that PER2::LUC rhythms remained observable (not consistent with predictions of the TTFL model), but with less robustness than wild type controls. Thus CRY confers robustness, not timekeeping, to cellular circadian rhythms (Putker *et al*, 2021).

The parallel CKO PER2::LUC recording from the cells used for proteomics analysis is now included in revised Supplementary Figure 1, as requested, but please note that any single set of recordings would not constitute evidence for or against the robustness of an oscillation. These data simply reflect the level of PER2::LUC reporter activity that was measured over the time window during which sample collection for mass spectrometry occurred.

3) From the text, it is not clear if biological replicates were run for the proteomics or if all samples were combined and run as one. This should be clear as it has implications as to the confidence in the changes that were seen between the WT and CKO cells.

As described in the methods section, for the proteomics an equal mixture of 3 technical replicates from a single biological replicate were sampled at each time point over full 3 circadian cycles, for

each genotype. Pooled samples were created by mixing a fixed amount of each sample (including all time points and both genotypes). Samples were then labelled with different tandem mass tags for multiplexed analysis. This was done in sets of 10, representing one cycle and genotype per mass spectrometry run (8 samples) together with 2 pooled samples. Protein abundance was standardised against the pooled samples during the data analysis. This is known as “internal reference scaling”, a standard method for eliminating batch effects in quantitative proteomics experiments (Plubell *et al*, 2017).

We have revised Figure 1A to communicate this more clearly as well as the accompanying section in the methods as follows:

Line 617: *“A timecourse was carried out as described above, with three technical replicates per genotype (i.e. 3 wells in a 6-well plate) at each timepoint. At each timepoint cells were washed twice in ice cold PBS and then lysed at room temperature in 100 µL lysis buffer (8 M urea, 20 mM Tris, pH 8) for 20 minutes. The lysis buffer was prepared the day before sampling began, and frozen in 1 mL aliquots. At each timepoint, one aliquot was defrosted at room temperature (23°C) whilst shaking at 700 rpm for 5 minutes. After lysis, the cells were scraped and technical replicates were combined, thus creating 1 sample per genotype at each time point. These samples were flash frozen immediately after collection in liquid nitrogen and stored at -80°C. After the time course was completed, all the samples were simultaneously defrosted and sonicated for 2 minutes. The protein concentration was then measured using a BCA assay (Pierce). 12 pooled samples were created by combining a portion of each experimental sample such that each sample/pool contained an equal mass of protein. All samples were then flash frozen in liquid nitrogen and stored at -80°C.”*

Finally, we stress that these cellular experiments were not designed to address biological variation within each genotype, they test for differences in temporal regulation of the proteome between non-transformed mouse cell lines that we have previously characterised, and behave no differently to many independently generated cell lines isolated from different mice. The clear rhythm of CRY1 abundance in wild type cells, and absence of CRY1 from CKO cells validates our experimental design for mass spectrometry (Figure S1). The cells were treated/sampled identically, at the same time, and the positive and negative controls produced the expected results, and there is therefore no reason to doubt our technical capability to measure changes in protein abundance over time or between genotypes. Whether or not a given protein is “rhythmic” is a largely semantic debate; we used two popular methods and these gave a similar result in the important and surprising respect that both found the CKO cellular (phospho)proteome to contain more rhythmic proteins and phosphopeptides than WT. Another major finding was the greater temporal variation of protein abundance in the absence of CRY, than in its presence. We then validated this finding in a post-hoc analysis of mouse liver from publicly available data generated by an independent lab. We agree that other methods will produce different numbers and identities of rhythmic proteins but it is implausible that another method of analysis would produce a qualitatively different answer. Moreover, our validation of the cellular results using data from mouse liver (multiple biological replicates) would generally be assumed to render superfluous any additional validation in cellular models.

4) If CRY is suppressing oscillations, it is possible that the rhythms in WT cells would decrease in amplitude over time and not be detected by eJTK. Metacycle or the PAICE suite are better are detecting these non-uniform oscillations and might give you are better idea of what is oscillating and what is not. The same is true for phase calls, neither RAIN nor eJTK is good at calling phase and is 2-4 hours off in most cases.

Thanks for this suggestion. With several hundred “rhythmic” proteins identified, phase estimates that are “off” by 10-20% would not be able to alter the temporal clustering we observe. We agree

that different algorithms will produce quantitively different numbers, identities and phases for “rhythmic” proteins in WT and CKO cells. They will not produce a qualitatively different result however i.e. that most rhythmically abundant proteins are most abundant within the same 6 hour window, irrespective of genotype.

5) The authors state that "the TTFL-independent circadian rhythm seen in CKO cells drives oscillations of higher relative amplitude and acts preferentially towards more abundant proteins and phosphopeptides" While I do see a correlation between the increased abundance and the oscillations, I do not see evidence that there is a preferential and direct drive in the oscillation higher abundance proteins. This statement either needs to be supported with more direct evidence or needs to be changed.

Thank you for pointing out this distinction, we have revised the text as follows:

Line 152: *“…the CRY-independent circadian rhythm seen in CKO cells produces oscillations of higher relative amplitude, with this trend being most evident for those proteins and phosphopeptides with higher overall abundance"*

6) While a P value might validate that these variations in protein abundance are "significantly different" in figure S2, a Cohen statistic will likely show that the change in variability is not meaningful. The authors should run a Cohen analysis to demonstrate that the small changes spread out over many proteins truly represent a meaningful difference.

These data are not normally distributed (Figure S2B, C, E); Cohen’s analysis would therefore be inappropriate. As we already report moreover, a clear majority of proteins in both genotypes, for both mouse tissue and isolated cells, change by <<10%, and so it is correct that the overall genotype-dependent difference in daily variance is small. However, the point we were trying to communicate with the graphs in S2B, C & E, was that more proteins change over time, and exhibit a greater level of temporal variation, in CKO cells/tissue compared with WT. We therefore very much thank the reviewer for encouraging us to find a clearer way to present these data. In revised Figure S2, we now use probability density functions to illustrate the greater number of proteins showing temporal variation in CKO cells/tissue, accompanied by appropriate statistics, which allows readers to judge the effect size for themselves. One thing that now becomes clear as a result of this suggestion, is that the increase in the level of temporal variation in protein abundance between CKO and WT, is even greater *in vivo* than in our cellular model system.

7) The authors state that there is a decrease in 20S alpha subunits. However, the supplement shows that at 12 hrs the 20S subunits are the same in the WT and the KO but different at 36 hrs. Though the authors state that the lack of differences at 12 hrs are likely due to the medium change, it could also be an induction due to the stress response in cells or it might be that their 36hr tpt was not accurate. More time points are needed to validate that the increase in 20S in WT (or decrease in CRY KO) is valid. In addition, there could be oscillations in the 20S subunits. This data needs to have more timepoints assessed before the authors draw conclusions about the role of CRY in the 20S proteosome.

Apologies, we should have been clearer here, mTOR-dependent acute induction of proteasome biogenesis by serum factors is well established (Dikic, 2017; Zhang *et al*, 2014b), and was included as a positive control. Whether wild type cells synthesise more proteasomes or degrade fewer proteasomes than CKO cells over a circadian cycle (i.e. under constant conditions) is beyond the scope of our study, and would not change the result that these CKO cells consistently have fewer proteasomes under the same conditions employed during the two main time series experiments (Figures 1+2 and Figure 4). Figure 3A already reports the quantification of mean abundance for

each of the 3 different proteasome catalytic subunits from the mass spectrometry time course averaged across all 24 different timepoints (now also plotted as a time series in revised Figure S4, and this shows no circadian variation). The western blot in 3B is simply an experimentally independent validation of this result at a specific timepoint in the same cell line at 36h after a media change. Immunoblotting is semi-quantitative (at best), and these quantifications are not helped by the difference in detected H3 levels (used as loading control). Ultimately though, these blots simply provide additional experimental support for the direction of change indicated by the very strong quantitative data shown in Figure 3A. Figure 3D then provides a further completely independent validation of the same finding by an orthogonal enzymatic assay for proteasome activity.

We have therefore measured different aspects of the same phenomenon using 3 independent methods, in 8 independent experiments. Whilst it is of course possible that further replicates might potentially yield a different result, we believe that overwhelming evidence is already presented for reduced proteasome activity in these cells. This feature provided some insight into the more general phenotype of CRY-deficient cells and tissues i.e. increased sensitivity to proteotoxic stress, which we go on to test in cells then validate *in vivo*.

8) The authors relate proteotoxic stress in the CRY KO to the lack of robustness in the clock. To show this, they added proteotoxic stress elicitors to WT cells and noted that the clock was damped. While this data is correlated, relieving proteotoxic stress in a CRY KO strain and seeing a return to robust oscillations would be the only way to demonstrate that the buffering of proteotoxic stress was the key role of CRY in the clock.

As described above, we agree with the reviewer's prediction that alleviation of proteotoxic stress should increase the robustness of PER2::LUC rhythms in CRY-deficient cells but we would not expect a complete rescue, as these cells would still lack canonical TTFL function, which is very important for robustness and amplification of PER2::LUC rhythms (Putker *et al*, 2021). At sub-saturating concentrations, cycloheximide reduces the rate of translational elongation and previously has been shown to alleviate several elements of proteotoxic stress in cultured cells (Kim & Strange, 2013; Peng *et al*, 2015; Parzych *et al*, 2015). We observed that CKO PER2::LUC rhythms became significantly more robust in the presence of low concentrations of cycloheximide, but importantly did not rescue them to wild type level. These data are now presented in Revised Figure 6. Please note that, as described in Putker et al (Putker *et al*, 2021), CKO PER2::LUC rhythms are intrinsically more variable between experiments and always less robust than in parallel WT control cells. It remains unclear whether this variation occurs due to variable fidelity of PER2::LUC as a post-translational circadian reporter or through less stable rhythm generation, or both. The observation of K^+ and cytosolic protein rhythms without any accompanying PER2::LUC rhythm in the same cell line (Figure 4E), but collected during a different experiment, argues for the latter.

****Minor Comments****

1) Much of the results reads like a discussion section and the discussion section repeats what is in the results. Consolidating and reorganizing these two sections will streamline the paper and make the conclusions much more obvious.

We do appreciate this advice. Our serious concern is to prevent any misunderstanding of our observations – we do not want readers to think we are suggesting that the TTFL model is wrong, rather we suggest that it is in need of refinement. Thus in the results section we aimed to simply report what was observed with minimal context and interpretation. In the discussion, we frame these observations among the broader scientific literature and propose what we believe to be the most parsimonious interpretation. Ultimately however there is only one important conclusion, that the CRY-mediated TTFL mechanism proposed as the fundamental basis for clock-controlled protein

rhythms in mammalian cells does not fulfil this function. If anything it suppresses protein rhythms. We have removed additional text from the results to try to improve narrative flow.

2) line 140 should be figure s1b.

Thanks.

3) line 401 synthesise is synthesize

Thank you. We have used the British English spelling.

Reviewer #1 (Significance (Required)):

The manuscript "CRYPTOCHROME suppresses the circadian proteome and promotes protein homeostasis" investigates the role that CRY plays in generating circadian rhythms, an important question as the essentiality of CRY in the clock for robustness has been established but the mechanistic role that CRY plays is much less clear. The authors find that a CRY KO leads to global changes in the proteome and phosphoproteomes. They also note that there is an increase in the number of oscillating proteins in the CRY KO, suggesting that CRY is a suppressor of oscillations. The authors also note that the changes in protein levels and oscillating proteins lead to the dysregulation of cellular stress responses. These results lead them to conclude that CRY KO phenotypes are less related to the dysfunction of the clock then they are to CRY specific-clock independent functions and that the main role of CRY in generating circadian robustness is in regulating proteotoxic stress. This work is of interest to most researchers who are interested in circadian rhythms. My expertise is in the fundamental regulation of circadian timing and output and nothing in this manuscript was beyond my expertise.

Reviewer #2 (Evidence, reproducibility and clarity (Required)):

Please find below the review, which I structured according to the guidelines given by Review Commons.
This review was compiled by David Gatfield, University of Lausanne.

****SECTION A - Evidence, reproducibility and clarity****

****Summary: Provide a short summary of the findings and key conclusions (including methodology and model system(s) where appropriate).****

The manuscript reports on how the circadian rhythmic proteomes and phosphoproteomes in mouse fibroblasts are affected by the knockout of the Cryptochrome genes (Cry1 and Cry2), encoding critical inhibitors of the transcriptional feedback loops that drive the molecular circadian oscillator in mammals. A large body of evidence from many labs has previously shown that mice lacking both Cry genes (CKO) are behaviourally arrhythmic - they are, in fact, one of the most frequently used genetic models for a clock-less animal. However, a very recent paper (Putker, [...], O'Neill, 2021, EMBO J 40(7):e106745) - from the same lab that is also submitting the current manuscript - has shown that under specific conditions (e.g. after entrainment through stronger environmental Zeitgeber) circadian rhythms are observable in CKO mice. In the previous paper, the authors put forward their idea that whereas circadian transcriptional feedback imparts robustness and functionality onto biological clocks, the core timekeeping mechanism is post-translational.

In the current manuscript, the authors continue along this line of research. They temperature-entrain CKO fibroblasts and control cells, release them into constant conditions, and sample them over 3 days (resolution 3 hours). In both cell lines, quantitative proteomics allowed the detection of thousands of individual proteins and phosphopeptides. The abundance of most proteins (82% of all detectable) and phosphopeptdes (68%) was significantly different between the two cell lines when averaging over all timepoints of the series. Moreover, when applying rhythmicity detection algorithms to the time series, almost 3-fold more rhythmically accumulating proteins were found in the CKO than in the WT cells, with only few rhythmic proteins in common. Of note, for the main rhythmicity analyses, relatively lenient rhythmicity criteria were applied (relative amplitude threshold of 10%; main detection algorithm RAIN; however, also the more stringent JTK method resulted in similar results). The ensemble of these findings - all in Figure 1 - are unexpected and intriguing given that normally less rhythmicity, rather than more rhythmicity, would be expected in cells in which the Cry1/2 genes are inactivated. Further analyses of the proteomics data, shown in Figure 2, explore more detailed rhythmic properties of the oscillating proteins. Proteins rhythmic in both cell lines have a moderately higher amplitude and are on average more abundant in CKO than in WT. Moreover, depending on which subsets of rhythmic events were analysed and compared (i.e. proteins, phosphoproteins, common, or exclusive to one of the two genotypes), phase distributions of the oscillating events were differentially affected. The intermediary conclusion that the authors make is that the role of CRY proteins is to suppress rhythmic events (proteins/phosphoproteins), which now, in the CKO cells, are unmasked and detectable.

In the rest of the manuscript the authors put together different ideas for the underlying mechanism(s) and show data that is globally consistent with these ideas. First, they hypothesise that in order to accumulate higher average (rhythmic) protein levels, proteasomal activity may be reduced and translation rate increased. Figure 3 shows data that indeed points in this direction, especially for the lower proteasomal activity. Another difference

between CKO and WT cells, shown in Figure 4, concerns the overall cytosolic protein content and - possibly coupled to this - the regulation of osmotic homeostasis, both of which show genotype-dependent differences in their daily patterns. These findings are consistent with the idea that in the absence of CRY proteins some sort of "proteomic imbalance" occurs - a state that may be reflected in higher sensitivity to proteotoxic stress; indeed, different assays shown in Figure 5 indicate that CKO cells have higher basic and inducible (by tunicamycin) levels of eIF2alpha phosphorylation (marker of integrated stress response), and CKO mice are sensitive to cellular stress induction as well (phospho-STAT3 levels after proteasome inhibitor treatment). Finally, in Figure 6, the authors induce proteotoxic stress conditions similar to what CKO cells experience under basic levels, now in WT cells. The observation is that under conditions of chronic stress (using 3 different inducers), rhythmicity of the circadian clock is reduced/abolished in WT cells, as judged from circadian reporter rhythms (transcriptionally driven PER2::LUC reporter). Overall, the authors conclude that CRY proteins have an important role in buffering overall protein biosynthesis against (circadian) fluctuations.

Thank you, this is a very accurate summary of our findings.

****Major comments:****

- Are the key conclusions convincing?

(1) Concerning the initial findings of more abundant rhythmicity in the CKO vs. WT cells (i.e. Figures 1-2), the conclusions are convincing within the limits of the applied experimental design. Thus, I am concerned that the data originate (if I understand this correctly) from a single cellular CKO and WT preparation (or pool of cells) that has been in culture for up to 30 passages.

The proteomics experiment used cells that had been passaged 20 times. This is now clarified in the methods section (Line 689). After isolation/selection, fibroblasts first need to be passaged ~10 times to overcome replicative senescence in atmospheric oxygen levels, then expanded to provide sufficient material for cryo-preservation and experimentation. Primary fibroblasts grow quite slowly, and proliferate best when 30-80% confluent, hence they are usually diluted ~1 in 3 during routine culture. Thus the number of cell doublings from 20 passages is much lower than for highly transformed cells such as U2OS or 3T3 cells (which are generally diluted 1 in 10 at each passage).

It is difficult to assess whether lower or higher detectable rhythmicity is a (to be a bit provocative: arbitrary?) feature of the different cellular preparations, or truly of the different genotypes. Many labs have experienced that in different cellular preparations, even when they are isogenic, there can be per se quite substantial differences in cellular morphology, gene expression, and many other parameters, including rhythmicity dynamics. Ideally, the experiment should therefore have included several biological replicates (i.e. independent cell preparations) or even a rescue experiment: re-expressing CRYs in the CKO line. Otherwise, I do not see how the authors can make a strong argument for a genotype-specific effect.

A single biological replicate allows us to confidently refute the hypothesis that CRY is essential for circadian regulation of clock-controlled protein abundance in mammalian cells, because these observations could not have been made if the hypothesis were true. The secondary finding from cellular models, that CRY suppresses temporal proteome variation, was then validated in mouse liver using published data from an independent lab that employed many biological replicates (Fig 2C). With high temporal resolution, technical replicates and 3 circadian cycles, the cellular study had the greatest sensitivity to detect temporal variation in a cellular proteome within the constraints of current technology. The liver data has lower temporal resolution (4 time points) but employed

multiple mice. Observing the same thing in two very different contexts, with different methodologies, allows us to argue for a genotype-specific effect.

However, we absolutely agree that the specific identity of proteins (and transcripts) that exhibit a significant circadian rhythm in abundance shows poor reproducibility between labs and even between independently performed experiments within the same lab. Indeed, in our recent study of rhythmic protein abundance across the yeast metabolic cycle (O'Neill *et al*, 2020), we found only 4% overlap in the identity of significantly rhythmic proteins within the exact same cell cultures, during a single experiment, where the only variable was dilution rate. These observations have led us to question one element of the current circadian paradigm, that TTFL-mediated circadian regulation of protein abundance is the central mechanism by which circadian regulation of cellular function is achieved – if it were we would expect the same proteins to vary by the same amount when experiments are repeated. Indeed, our recent observations are better consistent with the alternative hypothesis, that circadian rhythms in gene expression function to suppress daily variation in the abundance of most proteins in order to maintain protein homeostasis and minimise the bioenergetic cost of proteome renewal (O'Neill *et al*, 2020). In this case, the relatively low amplitude cell-autonomous rhythms in protein abundance detected by us and other labs across a range of biological contexts may reflect imprecise temporal protein quality control and would be expected to vary rather stochastically between different cell lines and experimental conditions.

Irrespective of whether this alternative hypothesis is correct, whilst the use of several independent biological replicates would provide much greater insight into the level of variation within each genotype, it would not provide more insight into physiological temporal proteome variation than is already provided by the post-hoc analysis of mouse liver data (Fig S2C).

Independently of this issue, instead of the interpretation that CRYs suppress rhythmicity that becomes unmasked in CKO cells, I am wondering whether the authors have also considered an alternative model according to which the difference in rhythmicity detectable under free-run actually lies in differential sensitivity to the temperature-entrainment protocol in CKO vs WT cells? Maybe CKO and WT cells do not differ in their rhythmic proteomes per se, but only in the efficiency with which one can synchronise the cells, which will then be reflected in the protein rhythms during free-run?

This is an interesting suggestion. Certainly, CKO cells are consistently more sensitive to temperature cycles than WT (Putker *et al*, 2021). However, if the differences we observe were attributable to greater synchronisation, then we would not observe so many proteins and phosphopeptides with altered overall abundance (which averages out any temporal variation). Secondly, we would observe a consistently narrower phase distribution of both proteins and phosphopeptides in CKO if the cells were more synchronised than WT, not the similar protein and broader phosphopeptide phase distributions we actually observe. Third, if the reviewer's hypothesis were correct, then we would expect a much greater overlap in the rhythmic proteome and phosphoproteome than was actually observed between CKO and WT cells. Finally, we have tested entrainment by temperature cycles in many different WT cell lines, and consistently find greater synchrony after 4 days compared with 2 days, but no difference between 4 days and 7 days i.e. after 4 days WT cells are already as synchronous as possible, whereas our previous work indicates that CKO cells tend to desynchronise more quickly that WT under constant conditions (Putker *et al*, 2021).

(2) The other findings (Figures 3-6) are overall convincing, but again with the caveat of using single CKO and WT preparations for many conclusions. However, this risk is hedged somewhat better here, through additional experiments (e.g. in mice).

Still, I would like to point out that while the findings in Figures 3-6 are generally consistent with the proposed model, there are overall relatively few experiments that actually attempt to challenge it. Much of the data thus remains suggestive and circumstantial.

We absolutely agree that a hypothesis must be robustly tested before it can be accepted. Thus, in this investigation, we specifically tested the long-standing prediction that CRY proteins are essential for circadian regulation of mammalian cellular proteomes (Sato *et al*, 2006; Kume *et al*, 1999; Ye *et al*, 2014). Our findings are incompatible with that hypothesis i.e. the observation of circadian protein abundance rhythms in a single CRY-deficient mammalian cell line is sufficient to refute the hypothesis that CRY is essential for the generation of circadian protein rhythms in all mammalian cells. We then propose an alternative model (Fig S7), informed by our observations (Figures 1-4), and test some key predictions from it (Figures 5-6). We do not claim that this alternative model is "true", merely that it more consistent with our own and previous observations than the prevailing paradigm. Suggested by reviewer 1, we now also include a test of the prediction that exogenous alleviation of proteotoxic stress increases the robustness of PER2::LUC rhythms in CKO cells.

As a further test of our model, the revised manuscript now includes behavioural data from mice treated with the proteasome inhibitor ixazomib in their drinking water (Figure 6). Our model predicts that cellular stress normally obscures the expression of circadian physiology and behaviour in mouse models. When rhythms are observed in CKO mice (Maywood *et al*, 2011; Ono *et al*, 2013; Putker *et al*, 2021), after strong environmental cues, the period of oscillation in constant darkness is shorter and the amplitude lower than wild type controls. We therefore simply tested the prediction that the mild continuous proteotoxic stress of oral ixazomib would reversibly shorten the period of mouse circadian rhythms and reduce their amplitude i.e. pushing the phenotype closer to CKO mice. Our results were consistent with this prediction. We do not expect a complete phenocopy since the level of serum ixazomib required to induce sufficient proteotoxic stress to recapitulate CRY-deficiency in the SCN would be likely to result in unacceptable welfare issues. We hope that the reviewer will agree that within the iterative cycle of the scientific method, there comes a natural point where observations should be communicated to the field, before the next cycle of prediction/experiment/refinement begins. We believe that we have reached that point and that the information provided in our study will be of great interest to the scientific community. The next cycle of experiments will be able to explicitly refute the alternative model for circadian proteostasis we are proposing, if it is incorrect, and we have absolutely no qualms about disproving our own hypotheses, e.g., Putker et al, ARS, 2017 (Putker *et al*, 2017).

We have revised the text as follows:
Line 336: *"Extending this hypothesis in vivo predicts that cellular stress normally obscures the expression of circadian physiology and behaviour in mouse models. When rhythms are observed in CKO mice (Maywood et al, 2011; Ono et al, 2013b; Putker et al, 2021), after strong environmental cues, the period of oscillation in constant darkness is shorter and the amplitude lower than wild type controls. We therefore tested the prediction that the mild continuous proteotoxic stress presented by a continuous low-dose of ixazomib (an orally available proteasome inhibitor) would reversibly shorten the period of circadian locomotor activity rhythms and reduce their amplitude i.e. pushing the phenotype closer to CKO mice. We found that amplitude was reduced and period length was shortened during 7 days of drug treatment of WT mice compared with vehicle controls, and reversible upon drug removal (Figure 6I-L). Therefore, chronic proteotoxic stress is sufficient to impair robustness of circadian rhythms in cells and mice. In principle this may contribute to the reduced robustness and timekeeping fidelity of CKO cells as well as diverse phenotypes of CRY-deficient mice (Figure 6M)."*

I also do not agree with the statement (page 10) that it is not necessary to interrogate the transcriptome in the CKO cells because this has been done previously. It would be important to know which of the rhythmicity changes (or, as a matter of fact, gene expression changes at large) show parallel transcriptomal effects as well, and which ones are truly translationally/post-translationally driven. I do not believe that the reference to historical controls is valid in this case - for an integrative view of the sources of changes in protein levels, it would be essential to know how the mRNA levels change within the same samples.

With the greatest respect, we disagree, since the transcriptome would provide minimal insight, if any. We already found that clock gene rhythms are effectively absent from CKO cells (Putker *et al*, 2021). More importantly, there are only two relevant factors that can confer a rhythm in abundance upon a given protein: these are its rate of synthesis and degradation. If one or both of these is circadian regulated then, dependent on their relative amplitudes and phases, a detectable circadian rhythm in steady state abundance of the protein might arise.

Proteome-wide circadian variation of protein turnover, by autophagy or ubiquitin-proteosome system, has been reported but its regulation is very poorly understood at this time (Wang *et al*, 2020; Desvergne *et al*, 2016). As the reviewer will be aware there are many factors that can affect the rate of synthesis of a given protein, i.e., codon usage vs aminoacyl-tRNA availability; subcellular localisation/sequestration of mRNA into condensates by RNA binding proteins; phosphoregulation of translation factors (particularly eIF2α, eIF3d, eIF4E1); UTR motifs (TOP, IRE, IRES, TISU, Kozak); frameshift-inducing secondary structures; microRNA binding sites vs microRNA availability; mRNA stability (~polyA tail length), and finally the cytosolic availability of the protein's encoding mRNA (not its overall cellular abundance). Thus, whilst it is plausible that in some cases daily variation in overall mRNA abundance might contribute to daily variation in abundance of the encoded protein, a correlation between the two can never demonstrate causation, given the wide range of other equally plausible mechanisms by which daily variation in protein abundance might arise. Indeed, it is perfectly plausible that rhythms in the abundance of a protein directly (or indirectly) stimulate rhythms in its transcription, rather than the other way around. This is a common homeostatic feedback mechanism observed for the Period and other genes such as the ERK signalling pathway (Nakayama *et al*, 2008; Shin *et al*, 2009) and the NFκB pathway (Ashall *et al*, 2009).

Moreover, it is now well-established and widely accepted that whole cell transcript levels are poorly predictive of protein levels (Liu *et al*, 2016b; Feltham *et al*, 2019; Ohtsuki *et al*, 2012; Cheng *et al*, 2016; Zapalska-Sozoniuk *et al*, 2019). Roughly 50% of rhythmic proteins in mouse liver and SCN have no corresponding cycling mRNA (Deery *et al*, 2009; Reddy *et al*, 2006). Moreover, in a study of mouse liver, half of the cycling proteome was delayed in phase by 6 hours compared to their corresponding mRNA, and 20% had no cycling transcript at all (Robles *et al*, 2014); similar findings have been demonstrated under diurnal conditions (Mauvoisin *et al*, 2014).

In cultured mammalian cells, relatively few transcripts exhibit a detectable circadian rhythm in abundance (Collins *et al*, 2021; Hughes *et al*, 2009; Hoffmann *et al*, 2014). In the few cases that this has been studied (in wild type cells), less than half of those few rhythmic cellular transcripts encode proteins whose abundance is also rhythmic, and only for less than half of these does the phase of mRNA abundance adequately account for the phase of the protein abundance. Even within this handful of proteins, that show circadian abundance rhythms in cell culture where the phase of mRNA rhythms is roughly coherent with encoded protein rhythms, there is no direct evidence that the rhythm in transcript level is directly responsible for the rhythm in protein abundance – the evidence is circumstantial, and ribosome profiling has never been performed over 2 circadian cycles, the minimum recommended to confidently claim a rhythm (Hughes *et al*, 2017).

Ultimately then, if we were to perform transcriptomics in CKO vs WT cells, the very best outcome we might expect is to observe that transcript rhythms correlate better with protein rhythms in WT cells than in CKO cells. This correlation would provide no more insight than currently exists, and would have no bearing on our finding that in cells, and *in vivo*, temporal variation in protein abundance is suppressed, not generated, by CRY proteins. For an integrative view, within each cell or cell culture we would need to be able to measure the rates of each protein's synthesis and degradation, combined with its overall abundance, transcript level, transcript availability and % of each transcript engaged by actively translating ribosomes. It is not possible to measure all of these simultaneously with current technologies, and even then it would probably not be sufficient to mechanistically explain the complex relationship between protein levels and transcript levels. We do not dismiss the enormous contribution to understanding of circadian physiology facilitated by technologic advances in transcriptomics during the last two decades, we merely assert that transcriptomics cannot provide insight into the specific hypothesis we are testing.

To emphasise this point more clearly, we have made the following change to the text:

Line 485: *"…Moreover the generally poor correlation between changes in whole cell transcriptome with changes in protein abundance and activity (Feltham et al, 2019; Ohtsuki et al, 2012; Cheng et al, 2016; Liu et al, 2016a; Zapalska-Sozoniuk et al, 2019; Eastman et al, 2018; Cenik et al, 2015) means that causal relationships cannot be reliably inferred in any case. In our future work we will determine the relative rates of protein synthesis and removal that generate rhythms in abundance. Once proteins are identified whose abundance rhythm is specifically attributable to a cell-autonomous rhythm in translation, it will be possible to determine the relative contribution made by any circadian variation in cytosolic mRNA availability and its ribosomal recruitment. Whole cell transcriptomics alone cannot provide immediate mechanistic insight, since this does not distinguish sequestered mRNA from the pool that is actually available for translation (Eastman et al, 2018; Ivanov et al, 2019)."*

- Should the authors qualify some of their claims as preliminary or speculative, or remove them altogether?

I would say that overall probably a number of claims could require considerable toning down - when reading the results part, several times I thought this would be better for discussion. I am not listing these points here in detail; further down in this review, I give a short compilation of some things I noticed while reading the manuscript.

Thanks, we have revised the results section to focus simply on what was observed, with minimal essential context.

- Would additional experiments be essential to support the claims of the paper? Request additional experiments only where necessary for the paper as it is, and do not ask authors to open new lines of experimentation.

The lack of true biological replicates (or rescue experiments) in the mass-spec data is problematic in my view.
I am not sure whether there is a less laborious (time & cost) way around doing such an experiment, but I am reluctant to accept that one can make quite substantial novel claims on CRY proteins based on a single cell preparation/cell line.

We understand the reviewer's concern. To reiterate, a single well-characterised cell line was used to test the hypothesis that CRY is essential for circadian regulation of the proteome in mammalian

cells. Our data are evidently sufficient to refute this hypothesis – it cannot be true if a single exception is observed. We also previously showed that many different CKO lines have the capacity to express PER2::LUC rhythms (Putker et al., EMBO J, 2021). The CKO line used in this investigation, (chosen randomly from 6 that were isolated from 6 independent mice) showed higher amplitude rhythms in independent time courses that employed several different methods (Figure 1,2 & 4). We agree that the increased number and amplitude of rhythmic proteins detected in CKO vs WT cells would not be sufficient to support our secondary finding that CRY functions to suppress circadian protein rhythms – if considered in isolation. This is why we interrogated *in vivo* data from multiple biological replicates that were collected and previously published by an independent lab (Fig S2C). We suggest that we are not making a novel claim, in that it has been assumed that CKO cells lack circadian proteome regulation because they lack an "essential clock gene", we are in fact simply testing this assumption and find it to be incorrect.

- Are the suggested experiments realistic in terms of time and resources? It would help if you could add an estimated cost and time investment for substantial experiments.

This is something I cannot judge.
But quality of the data and validity of the interpretation is for me the only thing that is relevant here.

It is not clear to us that a simple repeat of the same experiment with independent WT & CKO lines would provide any additional insight, as discussed on Page 1 of our response to reviewers. Here we have also proposed an experimental design and predictions that we think would address the reviewer's main concern and would appreciate their thoughts, however, it is not clear to us how this experiment could have any bearing on our original results or interpretation, irrespective of the outcome i.e. if we get a different result, it is still true CRY is not essential for circadian proteome rhythms; if we observe a smaller effect size, it would not change the larger variation we have already observed in CKO mouse liver compared with wild type (Figure S2C).

- Are the data and the methods presented in such a way that they can be reproduced?

Yes, I think this is overall well described.

- Are the experiments adequately replicated and statistical analysis adequate?

See above. This is where I see the main issue with the study in its present form!

In the revised manuscript, we now support all the major findings with validation *in vivo*. We hope the reviewer will agree that further cellular experiments might be interesting, but would not provide more new understanding than we have already gleaned.

****Minor comments:****
- Specific experimental issues that are easily addressable.

I have a list of individual comments just below.

- Are prior studies referenced appropriately?

Yes.

- Are the text and figures clear and accurate?

Overall yes.

- Do you have suggestions that would help the authors improve the presentation of their data and conclusions?

In the following I list a couple of points that the authors may find helpful and that are generally easy to correct:

-Page 4, line 74: "Similarly, circadian oscillations persist in cells and tissue slices lacking BMAL1 (37,38)" Given the problematic situation surrounding the paper Ref. 37 (Ray et al., 2020), the authors are probably better advised to not use it as an argument to support their story.

Thank you. We had no reason to doubt this study at the time of submission, but clearly subsequent revelations have called its methodology and conclusions into question. We have removed this citation from the manuscript.

-Page 6, line 109-110: "In our cellular time course, as expected, Cry1 was selectively detected in WT, but not in CKO cells, and [...] (Figure S1B).". It would be good to show all core clock protein data (where detectable) in WT vs CKO, as supplemental material.

CRY1 was the only “core clock protein” detected. This is now stated in the accompanying figure legend. As stated in the methods, the complete set of proteins detected is available in the material provided on Github.

-Figure S1D-G: In the heatmaps (S1D-E), a black line indicates the loss of a specific timepoint from the time series (explained in figure legend). However, individual protein plots in S1F-G appear to show all timepoints. How is this possible?

Thanks for noticing this oversight. This timepoint is now omitted throughout the proteomics time series.

-Page 7, line 129: "In this way we found that more abundant proteins were more likely to be rhythmic than less abundant proteins, but crucially, [...]". Can this simply by an effect of signal/noise, variation, statistical power, etc.??

Thanks, we also considered what other explanations could give rise to this intriguing observation. It is certainly true that sensitivity to detect a rhythm is associated with overall abundance, which is intuitive and has been reported previously (Laloum & Robinson-Rechavi, 2020). As stated in the text, the relationship between abundance and rhythmicity is much stronger for CKO than WT cells, both for proteins and phosphopeptides, whereas differences in overall abundance of highly abundant proteins/phosphopeptides is not so great. This argues in favour of the observation being a biologically “real” phenomenon compared with any kind of technical artifact. If it were some unexpected artifact, one would expect the same effect size irrespective of genotype. If we were to speculate, these observations may be consistent with a non-specific mechanism of protein degradation, obeying mass action kinetics, that is circadian regulated and with higher amplitude in CKO compared with WT cells. We hope to be able to explain these observations mechanistically in our future work, as this goes beyond the scope of this study.

-Page 7, line 140: Reference to (Figure S1A) should be corrected to (Figure S1B).

Thanks for noticing this, now revised.

-Page 8, line 158: "Secondly, in WT cells, CRY regulates the phase of rhythmicity for a subpopulation of proteins and phosphopeptides whose abundance peaks around the same phase as CRY1 and in antiphase with the majority of the rhythmic (phosphor)proteome." I am not sure this claim can be made here from the presented data, because the phase difference of the commonly rhythmic proteins is not shown?

Apologies for the misunderstanding, we worded this poorly. The point here is that amongst the proteins and phosphopeptides that are rhythmic in WT (and not in CKO), a small population are in antiphase to the majority of rhythmic proteins. The conclusion is that the rhythmicity of these "antiphasic" proteins/phosphopeptides is dependent on CRY, since they are not rhythmic in CKO cells and neither do we observe any such clustering at this phase in CKO cells. Therefore, showing the phase maps for proteins/phosphopeptides rhythmic in both genotypes does not add to our argument.

We have clarified this point in the text as follows:

Line 175: *"Secondly, in WT cells only, there is a subpopulation of proteins and phosphopeptides whose rhythmicity is dependent on CRY, and in antiphase to the majority of the rhythmic (phospho)proteome."*

Page 9 (line 186) - page 10 (line 209): all this is very speculative and hypothetical - rather something for discussion than for results section?

Thank you (now starting line 205). The first paragraph simply describes what was observed and so should correctly be in the results section. The second paragraph contains a summary of the preceding section that is required as essential context for the concept of proteome imbalance, but also did contain some interpretation that we agree would be better communicated in the discussion section. We have removed much of this in the revised manuscript because it is covered in the discussion already.

Page 10, lines 212-217. As already mentioned above, the fact that other publications have studied CKO transcriptional changes in other biological samples, paradigms etc., does not mean that the authors can ignore them in this study with, after all, other cells, other entrainment paradigms, etc.!

The authors are taking a shortcut that I find difficult to accept - transcriptomal changes should be taken into account in order to be sure what effects are really occurring at the protein level! Difficult to look at the protein in isolation.

These changes are evidently occurring at the level of protein abundance. We are entirely agnostic as to their mechanistic underpinnings. As described above there are many ways in which a circadian rhythm in the synthesis of a protein might be achieved, and three in which a rhythm in its removal from the cell might be accomplished (UPS, autophagy, exosomes). This study is focused explicitly on protein abundance in the steady state, in our future work we will determine the relative rates of protein synthesis and removal that generate rhythms in abundance (by pulsed SILAC-TMT). Once we know the identity of proteins whose abundance rhythm is specifically attributable to a cell-autonomous rhythm in translation, we can determine the relative contribution made by any circadian variation in cytosolic mRNA availability and its ribosomal recruitment. We do not anticipate that that whole cell transcriptomics alone can provide immediate mechanistic insight, since this does not distinguish mRNA that is available for translation from that which is sequestered

in non-membrane bound compartments (stress granules, p-bodies etc) or that which is bound by stalled ribosomes. To make this clearer, we have added to the discussion as follows:

Line 489: *"In our future work we will determine the relative rates of protein synthesis and removal that generate rhythms in abundance. Once proteins are identified whose abundance rhythm is specifically attributable to a cell-autonomous rhythm in translation, it will be possible to determine the relative contribution made by any circadian variation in cytosolic mRNA availability and its ribosomal recruitment. Whole cell transcriptomics alone cannot provide immediate mechanistic insight, since this does not distinguish sequestered mRNA from the pool that is actually available for translation (Eastman et al, 2018; Ivanov et al, 2019)."*

Page 13, line 286: "This may be reconciled by our observation that density regulated protein (DENR) and eIF2A. additional alternative subunits of the translation initiation complex. Were significantly upregulated in CKO cells compared with WT (Figure S6B-C);". The increase in DENR is so minimal, it is hard to imagine that this can explain anything. Honestly, I do not see the sense in this statement, it's unnecessary distraction and not hard data. In general, there are too many such suggestive claims in the results section that are then not followed up on.
BTW, DENR likely has very little, if any, role in canonical translation (but rather in re-initiation).

Thanks for this suggestion. It is entirely correlational we agree, we simply found the upregulation of eIF2a particularly interesting because it has been observed in the context of certain cancers. We have now removed these data and associated text from the revised manuscript.

Figure 3C vs. 3B: The quantification of the Western blot does not appear to reflect the experiment itself. On the membrane (3B), it does not look like a reduction down to <25 % that is show in the quanfication (3C); also the (supposedly more quantitative) mass-spec data suggests that the effect size is smaller. Did something go wrong in the quantification?

As the reviewer is aware, western blotting is only semi-quantitative and comparison between cells requires several replicates and appropriate loading controls. In this experiment, we decided to use histone H3 as a loading control since a previous mass spectrometry experiment revealed no significant circadian or genotype-dependent variation in H3 levels. We encountered more variation in H3 levels between replicate dishes than was expected, however, which has the effect of accentuating the apparent difference in 20S alpha subunits. We cannot justify retrospectively changing the loading control that we picked before doing the experiment, and in any case the result is consistent with the findings in 3A and 3D i.e. lower proteasome abundance/activity in CKO compared with WT cells.

Ultimately the western blots are included simply as an additional validation, of the independent (more quantitative) measurements that were performed independently by mass spec and enzyme assay and so there is no reason to doubt them.

Figure 3D: The signal in this assay depends on cell number or "total cytoplasmic content per well." In the Methods section, I saw that 20'000 cells were seeded per well. Question: at the time of experiment, were cell numbers equivalent (e.g. are proliferation rates - from seeding to measurement - of CKO vs. WT the same?), and are the cells actually of comparable size (i.e. do 20'000 CKO cells have about the same cytoplasmic equivalent as 20'000 WT cells)? The assay could be, for example, normalised to total protein content.

The cells were counted seeded about 12h before the experiment, so proliferation would have been minimal. Any differences in cell size do not affect our interpretation that proteasomal activity is lower in CKO cells compared with WT. Normalising to protein in this case would be inappropriate, since already know that there is more protein present in CKO cells compared with WT (Figure 3F). We have clarified the timings in the methods as follows:

Line 805: *"20,000 cells per well were plated in a 96-well plate using standard culture medium, and the medium was changed 9 hours later. 10 µM epoxomicin in the medium was used as negative control. 3 hours after this medium change/epoxomicin treatment, the ProteasomeGlo Cell-Based Assay (Promega) was used to measure proteasome catalytic activity."*

Figure 3E: The significance is indicated with (**), but the same graph in Figure S4C only carries one star for significance (*).

These are the same data but a different statistical test was employed since more conditions are being compared in the supplementary figure (Welsh's t-test in 3E, 2-way ANOVA with multiple comparisons tests in S4C). In both cases, the null hypothesis (no difference) was rejected.

Figure 3: How do low proteasome activity (=more stable proteins) and stronger protein amplitudes fit together? This is something I did not fully understand.

There are at least 5 ways in which this could occur:

1. Autophagic flux has been reported to be rhythmic (Wang *et al*, 2020). If this were also true in our cells, then decreased proteasomal degradation in CKO cells would demand a compensatory increase in the amount and amplitude of autophagy-mediated degradation in order maintain steady state total protein concentration overall – especially given that overall protein synthesis is higher in CKO cells.
2. If the rhythmic rates of both synthesis and degradation (in WT cells) of a protein oscillate with similar amplitude and phase, the protein's steady state abundance will be constant or oscillate with a low relative amplitude (<10% and tending towards 0%). Any change in the phase relationship between synthesis and degradation will increase the relative amplitude in steady state abundance. If the phase difference in CKO cells is much greater than WT the rhythm in steady state abundance may increase above 10% relative amplitude and thus be detected as "rhythmic" by the analysis criteria used in this manuscript.
3. Also for those proteins with a rhythmic rate of synthesis and degradation (in WT cells), where synthesis and degradation rates normally oscillate with similar amplitude and phase, then a decrease in the relative amplitude of degradation vs synthesis will increase the relative amplitude of oscillation in (dynamic) steady state abundance. If the decrease in degradation amplitude for CKO vs WT cells is sufficiently great, then the rhythm in steady state abundance may increase above 10% relative amplitude and thus be detected as "rhythmic" by the criteria used in this manuscript..
4. Same as 3, but increase in the relative amplitude of synthesis vs degradation.
5. Any combination of 1-4.

We have attempted to explain a simplified version of this reasoning in Supplementary Figure 2 and the accompanying text. To distinguish between these possible mechanisms, for the entire proteome, lies well beyond the scope of this investigation. We will be able to address this question soon using a refinement of pulsed SILAC-TMT that is currently underway.

Figure 5A: Are p-eIF2alpha peptides detected in the original mass spec data? If yes, can the quantification be added to this Figure?

P-eIF2alpha peptides were not detected. Unfortunately our phosphoproteomics coverage was not as high as we would have liked.

Figure 5C: the WT animals are rhythmic, but the CKOs are not. Is this a concern (confounding factor) in this experiment?

These animals were maintained under diurnal conditions, under which there is essentially no discernible difference in behavioural activity i.e. rhythms in CKO animals are considered to be driven by the LD and associated feeding cycle (Vollmers *et al*, 2009; Mauvoisin *et al*, 2014; Crosby *et al*, 2019; Wang *et al*, 2017). This is readily observable from the behavioural data in Revised Figure 6. We have revised the figure 5 legend to make this clearer.

Reviewer #2 (Significance (Required)):

****SECTION B - Significance****

- Describe the nature and significance of the advance (e.g. conceptual, technical, clinical) for the field.

The nature and significance of the advance would be "conceptual", representing a new way of how we may think about the role of CRY proteins for gene expression - inside and outside of the clock!

- Place the work in the context of the existing literature (provide references, where appropriate).

From the original view - held until a few years ago - that CRY proteins are exclusively active as components of the core clock negative limb, broader functions have been documented over the last years. In particular, work by the Lamia lab and by others has shown a role as a modulator of nuclear receptor-mediated transcription - giving CRYs also a role in output pathways. In this context, further investigations into other/additional roles of CRYs are important and welcome.

- State what audience might be interested in and influenced by the reported findings.

I think that (for sure in the current state in which the authors do not provide a molecular explanation of how CRYs supposedly regulate the various proposed processes - from proteasome regulation, to translation etc.) this work would be mainly interesting for the broader circadian clock community.

The reviewer implicitly supposes that CRY proteins contribute to the regulation of protein homeostasis by a specific mechanism, but we suggest the logic here is unsound. Considering the many established targets of CRY, and the pleiotropic phenotype of other E3 ligase and transcriptional repressor knockouts (Huber *et al*, 2016; Correia *et al*, 2019; Lamia *et al*, 2009, 2011; Kriebs *et al*, 2017; Chan *et al*, 2020; Koike *et al*, 2012; Hoffmann & Spengler, 2019; Lombardi *et al*, 2015; Scheffner & Kumar, 2014; Zhou *et al*, 2013; Liu *et al*, 2020), we consider that direct regulation of translation or proteasomal activity by CRY is extremely unlikely – apart from anything else, there are orders of magnitude more ribosomes and proteosomes in the cell than there are copies of the CRY protein. The idea that deletion of a ubiquitously expressed, multifunctional protein would elicit many complex consequences, that compensate for its loss-of-function to maintain cellular function, is not novel or even surprising, and we cite several precedents in the

manuscript. Certainly, it cannot be assumed from our data that protein synthesis and proteasomal activity are directly regulated by CRY in WT cells.

To be clear once more, the central finding is that CRY is not required for circadian regulation of the cellular proteome, a direct experimental refutation of the mechanism that is broadly accepted to underpin daily regulation of mammalian cell biology. In cells and mouse tissue we observed greater temporal variation of more proteins in CKO cells than WT controls, thus CRY suppresses circadian regulation of the proteome in two independent biological contexts; again in stark contradiction of predictions from the currently accepted model. This is not to suggest that the currently accepted model is without virtue, merely that it is in need of urgent refinement.

Finally, given the importance of protein homeostasis for all cellular functions, and its causal role in many cancers and age-linked pathologies, we hope that the reviewer will agree that the association between CRY and protein homeostasis is of broader scientific interest than to the circadian field exclusively.

- Define your field of expertise with a few keywords to help the authors contextualize your point of view. Indicate if there are any parts of the paper that you do not have sufficient expertise to evaluate.

My expertise: Circadian clocks, RNA biology/translation, gene expression more broadly.

References

Anckar J & Sistonen L (2011) Regulation of HSF 1 Function in the Heat Stress Response: Implications in Aging and Disease. *Annu Rev Biochem* 80: 1089–1115

Aragón JJ & Sols A (1991) Regulation of enzyme activity in the cell: effect of enzyme concentration. *FASEB J* 5: 2945–2950

Ashall L, Horton CA, Nelson DE, Paszek P, Harper C V, Sillitoe K, Ryan S, Spiller DG, Unitt JF, Broomhead DS, *et al* (2009) Pulsatile stimulation determines timing and specificity of NF-kappaB-dependent transcription. *Science* 324: 242–6

Barclay JL, Shostak A, Leliavski A, Tsang AH, Johren O, Muller-Fielitz H, Landgraf D, Naujokat N, van der Horst GTJ & Oster H (2013) High-fat diet-induced hyperinsulinemia and tissue-specific insulin resistance in Cry-deficient mice. *AJP Endocrinol Metab* 304: E1053–E1063

Bulik S, Holzhütter HG & Berndt N (2016) The relative importance of kinetic mechanisms and variable enzyme abundances for the regulation of hepatic glucose metabolism - insights from mathematical modeling. *BMC Biol* 14

Bur IM, Cohen-Solal AM, Carmignac D, Abecassis PY, Chauvet N, Martin AO, van der Horst GTJ, Robinson ICAF, Maurel P, Mollard P, *et al* (2009) The circadian clock components CRY1 and CRY2 are necessary to sustain sex dimorphism in mouse liver metabolism. *J Biol Chem* 284: 9066–73

Cederroth CR, Albrecht U, Bass J, Brown SA, Dyhrfjeld-Johnsen J, Gachon F, Green CB, Hastings MH, Helfrich-Förster C, Hogenesch JB, *et al* (2019) Medicine in the Fourth Dimension. *Cell Metab* 30: 238–250 doi:10.1016/j.cmet.2019.06.019 [PREPRINT]

Cenik C, Cenik ES, Byeon GW, Grubert F, Candille SI, Spacek D, Alsallakh B, Tilgner H, Araya CL, Tang H, *et al* (2015) Integrative analysis of RNA, translation, and protein levels reveals distinct regulatory variation across humans. *Genome Res* 25: 1610–1621

Chan AB, Huber AL & Lamia KA (2020) Cryptochromes modulate E2F family transcription factors. *Sci Rep* 10

Cheng Z, Teo G, Krueger S, Rock TM, Koh HW, Choi H & Vogel C (2016) Differential dynamics of the mammalian mRNA and protein expression response to misfolding stress . *Mol Syst Biol* 12: 855

Collins EJ, Cervantes-Silva MP, Timmons GA, O'Siorain JR, Curtis AM & Hurley JM (2021) Post-transcriptional circadian regulation in macrophages organizes temporally distinct immunometabolic states. *Genome Res* 31: 171–185

Correia SP, Chan AB, Vaughan M, Zolboot N, Perea V, Huber A-L, Kriebs A, Moresco JJ, Yates JR & Lamia KA (2019) The circadian E3 ligase complex SCFFBXL3+CRY targets TLK2. *Sci Rep* 9: 198

Cox KH & Takahashi JS (2019) Circadian clock genes and the transcriptional architecture of the clock mechanism. *J Mol Endocrinol* 63: R93–R102

Crosby P, Hamnett R, Putker M, Hoyle NP, Reed M, Karam CJ, Maywood ES, Stangherlin A, Chesham JE, Hayter EA, *et al* (2019) Insulin/IGF-1 Drives PERIOD Synthesis to Entrain Circadian Rhythms with Feeding Time. *Cell* 177: 896-909.e20

Deery MJ, Maywood ES, Chesham JE, Sládek M, Karp NA, Green EW, Charles PD, Reddy AB, Kyriacou CP, Lilley KS, *et al* (2009) Proteomic analysis reveals the role of synaptic vesicle cycling in sustaining the suprachiasmatic circadian clock. *Curr Biol* 19: 2031–6

Desvergne A, Ugarte N, Radjei S, Gareil M, Petropoulos I & Friguet B (2016) Circadian modulation of proteasome activity and accumulation of oxidized protein in human embryonic kidney HEK 293 cells and primary dermal fibroblasts. *Free Radic Biol Med* 94: 195–207

Dikic I (2017) Proteasomal and Autophagic Degradation Systems. *Annu Rev Biochem* 86: 193–224

Dunlap JC (1999) Molecular Bases for Circadian Clocks. *Cell* 96: 271–290

Eastman G, Smircich P & Sotelo-Silveira JR (2018) Following Ribosome Footprints to Understand Translation at a Genome Wide Level. *Comput Struct Biotechnol J* 16: 167–176 doi:10.1016/j.csbj.2018.04.001 [PREPRINT]

Elowitz MB, Levine AJ, Siggia ED & Swain PS (2002) Stochastic gene expression in a single cell. *Science (80-)* 297: 1183–1186

Fan Y, Hida A, Anderson DA, Izumo M & Johnson CH (2007) Cycling of CRYPTOCHROME proteins is not necessary for circadian-clock function in mammalian fibroblasts. *Curr Biol* 17: 1091–100

Feltham J, Xi S, Murray S, Wouters M, Urdiain-Arraiza J, George C, Townley A, Roberts E, Fisher R, Liberatori S, *et al* (2019) Transcriptional changes are regulated by metabolic pathway dynamics but decoupled from protein levels. *bioRxiv*: 833921 doi:10.1101/833921 [PREPRINT]

Franks A, Airoldi E & Slavov N (2017) Post-transcriptional regulation across human tissues. *PLoS Comput Biol* 13

Harper JW & Bennett EJ (2016) Proteome complexity and the forces that drive proteome imbalance. *Nature* 537: 328–338

Hetz C & Saxena S (2017) ER stress and the unfolded protein response in neurodegeneration. *Nat Rev Neurol* 13: 477–491

Hipp MS, Kasturi P & Hartl FU (2019) The proteostasis network and its decline in ageing. *Nat Rev Mol Cell Biol* 20: 421–435

Hoffmann A & Spengler D (2019) Chromatin remodeling complex NuRD in neurodevelopment and neurodevelopmental disorders. *Front Genet* 10 doi:10.3389/fgene.2019.00682 [PREPRINT]

Hoffmann J, Symul L, Shostak A, Fischer T, Naef F & Brunner M (2014) Non-Circadian Expression Masking Clock-Driven Weak Transcription Rhythms in U2OS Cells. *PLoS One* 9: e102238

Huber AL, Papp SJ, Chan AB, Henriksson E, Jordan SD, Kriebs A, Nguyen M, Wallace M, Li Z, Metallo CM, *et al* (2016) CRY2 and FBXL3 Cooperatively Degrade c-MYC. *Mol Cell* 64: 774–789

Hughes ME, Abruzzi KC, Allada R, Anafi R, Arpat AB, Asher G, Baldi P, de Bekker C, Bell-Pedersen D, Blau J, *et al* (2017) Guidelines for Genome-Scale Analysis of Biological Rhythms. *J Biol Rhythms* 32: 380–393

Hughes ME, DiTacchio L, Hayes KR, Vollmers C, Pulivarthy S, Baggs JE, Panda S & Hogenesch JB (2009) Harmonics of Circadian Gene Transcription in Mammals. *PLoS Genet* 5: e1000442

Ivanov P, Kedersha N & Anderson P (2019) Stress granules and processing bodies in translational control. *Cold Spring Harb Perspect Biol* 11

Jang C, Lahens NF, Hogenesch JB & Sehgal A (2015) Ribosome profiling reveals an important role for translational control in circadian gene expression. *Genome Res*: 1836–1847

Kim H & Strange K (2013) Changes in translation rate modulate stress-induced damage of diverse proteins. *Am J Physiol - Cell Physiol* 305

Koike N, Yoo S-H, Huang H-C, Kumar V, Lee C, Kim T-K & Takahashi JS (2012) Transcriptional architecture and chromatin landscape of the core circadian clock in mammals. *Science* 338: 349–54

Kriebs A, Jordan SD, Soto E, Henriksson E, Sandate CR, Vaughan ME, Chan AB, Duglan D, Papp SJ, Huber A-L, *et al* (2017) Circadian repressors CRY1 and CRY2 broadly interact with nuclear receptors and modulate transcriptional activity. *Proc Natl Acad Sci* 114: 8776–8781

Kume K, Zylka MJ, Sriram S, Shearman LP, Weaver DR, Jin X, Maywood ES, Hastings MH & Reppert SM (1999) mCRY1 and mCRY2 are essential components of the negative limb of the circadian clock feedback loop. *Cell* 98: 193–205

Laloum D & Robinson-Rechavi M (2020) Methods detecting rhythmic gene expression are biologically relevant only for strong signal. *PLOS Comput Biol* 16: e1007666

Lamia KA, Papp SJ, Yu RT, Barish GD, Uhlenhaut NH, Jonker JW, Downes M & Evans RM (2011) Cryptochromes mediate rhythmic repression of the glucocorticoid receptor. *Nature* 480: 552–556

Lamia KA, Sachdeva UM, DiTacchio L, Williams EC, Alvarez JG, Egan DF, Vasquez DS, Juguilon H, Panda S, Shaw RJ, *et al* (2009) AMPK regulates the circadian clock by

cryptochrome phosphorylation and degradation. *Science* 326: 437–40
Liu H, Ding J, Köhnlein K, Urban N, Ori A, Villavicencio-Lorini P, Walentek P, Klotz LO, Hollemann T & Pfirrmann T (2020) The GID ubiquitin ligase complex is a regulator of AMPK activity and organismal lifespan. *Autophagy* 16: 1618–1634
Liu Y, Beyer A & Aebersold R (2016a) On the Dependency of Cellular Protein Levels on mRNA Abundance. *Cell* 165: 535–550 doi:10.1016/j.cell.2016.03.014 [PREPRINT]
Liu Y, Beyer A & Aebersold R (2016b) On the Dependency of Cellular Protein Levels on mRNA Abundance. *Cell* 165: 535–550
Lombardi LM, Baker SA & Zoghbi HY (2015) MECP2 disorders: From the clinic to mice and back. *J Clin Invest* 125: 2914–2923 doi:10.1172/JCI78167 [PREPRINT]
Lowrey PL & Takahashi JS (2011) Genetics of circadian rhythms in mammalian model organisms
Lück S & Westermark PO (2016) Circadian mRNA expression: Insights from modeling and transcriptomics. *Cell Mol Life Sci* 73: 497–521 doi:10.1007/s00018-015-2072-2 [PREPRINT]
Masuki S, Todo T, Nakano Y, Okamura H & Nose H (2005) Reduced α-adrenoceptor responsiveness and enhanced baroreflex sensitivity in Cry-deficient mice lacking a biological clock. *J Physiol* 566: 213–224
Mauvoisin D, Wang J, Jouffe C, Martin E, Atger F, Waridel P, Quadroni M, Gachon F & Naef F (2014) Circadian clock-dependent and -independent rhythmic proteomes implement distinct diurnal functions in mouse liver. *Proc Natl Acad Sci U S A* 111: 167–72
Maywood ES, Chesham JE, O'Brien JA & Hastings MH (2011) A diversity of paracrine signals sustains molecular circadian cycling in suprachiasmatic nucleus circuits. *Proc Natl Acad Sci* 108: 14306–14311
Nadaraia S, Yohrling GJ, Jiang GC-T, Flanagan JM & Vrana KE (2007) Enzyme Activity: Control. In *Encyclopedia of Life Sciences* Chichester, UK: John Wiley & Sons, Ltd
Nakayama K, Satoh T, Igari A, Kageyama R & Nishida E (2008) FGF induces oscillations of Hes1 expression and Ras/ERK activation. *Curr Biol* 18: R332-4
Nangle SN, Rosensweig C, Koike N, Tei H, Takahashi JS, Green CB, Zheng N, Adams P, Afonine P, Bunkoczi G, *et al* (2014) Molecular assembly of the period-cryptochrome circadian transcriptional repressor complex. *Elife* 3: e03674
Neufeld-Cohen A, Robles MS, Aviram R, Manella G, Adamovich Y, Ladeuix B, Nir D, Rousso-Noori L, Kuperman Y, Golik M, *et al* (2016) Circadian control of oscillations in mitochondrial rate-limiting enzymes and nutrient utilization by PERIOD proteins. *Proc Natl Acad Sci U S A* 113: E1673–E1682
O'Neill JS, Hoyle NP, Robertson JB, Edgar RS, Beale AD, Peak-Chew SY, Day J, Costa ASH, Frezza C & Causton HC (2020) Eukaryotic cell biology is temporally coordinated to support the energetic demands of protein homeostasis. *Nat Commun* 11
Ohtsuki S, Schaefer O, Kawakami H, Inoue T, Liehner S, Saito A, Ishiguro N, Kishimoto W, Ludwig-Schwellinger E, Ebner T, *et al* (2012) Simultaneous absolute protein quantification of transporters, cytochromes P450, and UDP-glucuronosyltransferases as a novel approach for the characterization of individual human liver: Comparison with mRNA levels and activities. *Drug Metab Dispos* 40: 83–92
Ono D, Honma S & Honma KI (2013) Postnatal constant light compensates Cryptochrome1 and 2 double deficiency for disruption of circadian behavioral rhythms in mice under constant dark. *PLoS One* 8
Pakos-Zebrucka K, Koryga I, Mnich K, Ljujic M, Samali A & Gorman AM (2016) The integrated stress response. *EMBO Rep* 17: 1374–1395
Parzych K, Chinn TM, Chen Z, Loaiza S, Porsch F, Valbuena GN, Kleijnen MF, Karadimitris A, Gentleman E, Keun HC, *et al* (2015) Inadequate fine-tuning of protein synthesis and failure of amino acid homeostasis following inhibition of the ATPase VCP/p97. *Cell Death Dis* 6: e2031
Pedraza JH & Van Oudenaarden A (2005) Noise propagations in gene networks. *Science (80-)* 307: 1965–1969
Peng M, Ostrovsky J, Kwon YJ, Polyak E, Licata J, Tsukikawa M, Marty E, Thomas J, Felix CA,

Xiao R, *et al* (2015) Inhibiting cytosolic translation and autophagy improves health in mitochondrial disease. *Hum Mol Genet* 24: 4829–4847

Plubell DL, Wilmarth PA, Zhao Y, Fenton AM, Minnier J, Reddy AP, Klimek J, Yang X, David LL & Pamir N (2017) Extended Multiplexing of Tandem Mass Tags (TMT) Labeling Reveals Age and High Fat Diet Specific Proteome Changes in Mouse Epididymal Adipose Tissue. *Mol Cell Proteomics* 16: 873–890

Putker M, Crosby P, Feeney KA, Hoyle NP, da Costa ASH, Gaude E, Frezza C & O'Neill JS (2017) Mammalian circadian period, but not phase and amplitude, is robust against redox and metabolic perturbations. *Antioxid Redox Signal* 4523: ars.2016.6911

Putker M, Wong DCS, Seinkmane E, Rzechorzek NM, Zeng A, Hoyle NP, Chesham JE, Edwards MD, Feeney KA, Fischer R, *et al* (2021) CRYPTOCHROMES confer robustness, not rhythmicity, to circadian timekeeping. *EMBO J* e106745: 1–15

Raser JM & O'Shea EK (2004) Control of stochasticity in eukaryotic gene expression. *Science (80-)* 304: 1811–1814

Reddy AB, Karp NA, Maywood ES, Sage EA, Deery M, O'Neill JS, Wong GKY, Chesham J, Odell M, Lilley KS, *et al* (2006) Circadian Orchestration of the Hepatic Proteome. *Curr Biol* 16: 1107–1115

Robles MS, Cox J & Mann M (2014) In-Vivo Quantitative Proteomics Reveals a Key Contribution of Post-Transcriptional Mechanisms to the Circadian Regulation of Liver Metabolism. *PLoS Genet* 10

Rocca JD, Hall EK, Lennon JT, Evans SE, Waldrop MP, Cotner JB, Nemergut DR, Graham EB & Wallenstein MD (2015) Relationships between protein-encoding gene abundance and corresponding process are commonly assumed yet rarely observed. *ISME J* 9: 1693–1699 doi:10.1038/ismej.2014.252 [PREPRINT]

Sasaki K & Hiderou Y (2015) Organelle autoregulation—stress responses in the ER, Golgi,mitochondria and lysosome. *J Biochem* 157: 185–195

Sato TK, Yamada RG, Ukai H, Baggs JE, Miraglia LJ, Kobayashi TJ, Welsh DK, Kay SA, Ueda HR & Hogenesch JB (2006) Feedback repression is required for mammalian circadian clock function. *Nat Genet* 38: 312–319

Scheffner M & Kumar S (2014) Mammalian HECT ubiquitin-protein ligases: Biological and pathophysiological aspects. *Biochim Biophys Acta - Mol Cell Res* 1843: 61–74

Shin S-Y, Rath O, Choo S-M, Fee F, McFerran B, Kolch W & Cho K-H (2009) Positive- and negative-feedback regulations coordinate the dynamic behavior of the Ras-Raf-MEK-ERK signal transduction pathway. *J Cell Sci* 122: 425–35

Sigal A, Milo R, Cohen A, Geva-Zatorsky N, Klein Y, Liron Y, Rosenfeld N, Danon T, Perzov N & Alon U (2006) Variability and memory of protein levels in human cells. *Nature* 444: 643–646

Takasu NN, Nakamura TJ, Tokuda IT, Todo T, Block GD & Nakamura W (2015) Recovery from Age-Related Infertility under Environmental Light-Dark Cycles Adjusted to the Intrinsic Circadian Period. *Cell Rep* 12: 1407–1413

Volfson D, Marciniak J, Blake WJ, Ostroff N, Tsimring LS & Hasty J (2006) Origins of extrinsic variability in eukaryotic gene expression. *Nature* 439: 861–864

Vollmers C, Gill S, DiTacchio L, Pulivarthy SR, Le HD & Panda S (2009) Time of feeding and the intrinsic circadian clock drive rhythms in hepatic gene expression. *Proc Natl Acad Sci* 106: 21453–21458

Wang D, Eraslan B, Wieland T, Hallström B, Hopf T, Zolg DP, Zecha J, Asplund A, Li L, Meng C, *et al* (2019) A deep proteome and transcriptome abundance atlas of 29 healthy human tissues. *Mol Syst Biol* 15

Wang J, Mauvoisin D, Martin E, Atger F, Galindo AN, Dayon L, Sizzano F, Palini A, Kussmann M, Waridel P, *et al* (2017) Nuclear Proteomics Uncovers Diurnal Regulatory Landscapes in Mouse Liver. *Cell Metab* 25: 102–117

Wang X, Xu Z, Cai Y, Zeng S, Peng B, Ren X, Yan Y & Gong Z (2020) Rheostatic Balance of

Circadian Rhythm and Autophagy in Metabolism and Disease. *Front Cell Dev Biol* 8: 616434 doi:10.3389/fcell.2020.616434 [PREPRINT]
Wolff S, Weissman JS & Dillin A (2014) Differential Scales of Protein Quality Control. *Cell* 157: 52–64
Wong DC & O'Neill JS (2018) Non-transcriptional processes in circadian rhythm generation. *Curr Opin Physiol* 5: 117–132
Ye R, Selby CP, Chiou Y-Y, Ozkan-Dagliyan I, Gaddameedhi S & Sancar A (2014) Dual modes of CLOCK:BMAL1 inhibition mediated by Cryptochrome and Period proteins in the mammalian circadian clock. *Genes Dev* 28: 1989–98
Zapalska-Sozoniuk M, Chrobak L, Kowalczyk K & Kankofer M (2019) Is it useful to use several "omics" for obtaining valuable results? *Mol Biol Rep* 46: 3597–3606 doi:10.1007/s11033-019-04793-9 [PREPRINT]
Zhang R, Lahens NF, Ballance HI, Hughes ME & Hogenesch JB (2014a) A circadian gene expression atlas in mammals: Implications for biology and medicine. *Proc Natl Acad Sci* 111: 16219–16224
Zhang Y, Nicholatos J, Dreier JR, Ricoult SJH, Widenmaier SB, Hotamisligil GS, Kwiatkowski DJ & Manning BD (2014b) Coordinated regulation of protein synthesis and degradation by mTORC1. *Nature* 513: 440–443
Zhao Q, Wang J, Levichkin I V., Stasinopoulos S, Ryan MT & Hoogenraad NJ (2002) A mitochondrial specific stress response in mammalian cells. *EMBO J* 21: 4411–4419
Zhou W, Wei W & Sun Y (2013) Genetically engineered mouse models for functional studies of SKP1-CUL1-F-box-protein (SCF) E3 ubiquitin ligases. *Cell Res* 23: 599–619 doi:10.1038/cr.2013.44 [PREPRINT]

1st Editorial Decision 22nd Jun 2021

Dear John,

Thank you for transferring your manuscript from Review Commons to The EMBO Journal. I have now read your manuscript, the reviewer comments and your rebuttal, and discussed the scope of the revision with reviewer #1.

I note that both reviewers express interest in the study, but also indicate a major issue with the lack of biological replicates in the provided proteomics analysis. I have consulted further on this point with reviewer #1, who finds that, although the added reanalysis of a published dataset provides support to the findings, the manuscript would need further revision before they can recommend its publication. From the editorial side, I find that the addition of further proteomics analysis as proposed in your rebuttal would significantly strengthen the manuscript and its message and would therefore ask you to add this data in the final revised manuscript.

We have extended our 'scooping protection policy' beyond the usual 3-month revision timeline to cover the period required for a full revision to address the essential experimental issues. This means that competing manuscripts published during revision period will not negatively impact on our assessment of the conceptual advance presented by your study. Please contact us if you see a paper with related content published elsewhere to discuss the appropriate course of action.

When preparing your letter of response to the referees' comments, please bear in mind that this will form part of the Review Process File and will therefore be available online to the community. For more details on our Transparent Editorial Process, please visit our website: https://www.embopress.org/page/journal/14602075/authorguide#transparentprocess

Please note that I will be on maternity leave from June 23, and my colleague David del Alamo, with whom I have already discussed your study, will take care of your manuscript in my absence.

With best regards,

Ieva

Ieva Gailite, PhD
Scientific Editor
The EMBO Journal
Meyerhofstrasse 1
D-69117 Heidelberg
Tel: +4962218891309
i.gailite@embojournal.org

***
Rev_Com_number: RC-2021-00727
New_manu_number: EMBOJ-2021-108883
Corr_author: O'Neill
Title: CRYPTOCHROME suppresses the circadian proteome and promotes protein homeostasis

Reviewer #1 additional comments:

In brief, I don't think that they have done is sufficient to warrant publication at this stage, but I don't think that it is essential for them to do more proteomics.

To explain further: After reading the responses, I think that the authors have two choices. Either 1. They do more proteomics to give their results section a sound footing statistically, OR 2. They decrease the emphasis that they put on figures 1 and 2. The way that their paper is written, they base their entire hypothesis on one in vitro replicate of deeply sampled data and a second sparsely sampled biological triplicate in vivo set of data from a different author (the existing liver proteome that they had in before but have now played up a bit more). Neither of these data sets has sufficient data in order to get statistically meaningful data to strongly support their hypothesis. They then present this data in figure 1 and 2 and S1 and S2 and analyze the phase and the overlap of these oscillations. Their data is consistent BUT their data is not well sampled enough for a confident argument. Proteomics data, especially phosphoproteomics data, is way too messy for a single replicate. I don't believe that they need multiple different cell types, just more biological replication.
In all honestly, I think that their hypothesis in the paper is probably right, and will be backed up by more replication. However, there is too much noise in proteomic data for a single biological replicate to be sufficient for the kinds of statements they are making about the data. I think that their figures 3-6 tell the story without the need for the omics analysis and if they do not want to, or cannot afford to, do a more detailed proteomics analysis, then they should deemphasize their data in figures 1 and 2 and refocus their paper on figures 3-6.
I would be happy to provide a thorough response to this new version when the authors are ready. I do think that it adds to the conversation about CRY in the circadian field. However, I don't feel that their omics are strong enough to stand alone in the way that they are framing them and they would need to change something in order for me to support publication.

RE: EMBOJ-2021-108883R – response to reviewers

Reviewer 1: "In brief, I don't think that they have done is sufficient to warrant publication at this stage, but I don't think that it is essential for them to do more proteomics.

To explain further: After reading the responses, I think that the authors have two choices. Either 1. They do more proteomics to give their results section a sound footing statistically, OR 2. They decrease the emphasis that they put on figures 1 and 2. The way that their paper is written, they base their entire hypothesis on one in vitro replicate of deeply sampled data and a second sparsely sampled biological triplicate in vivo set of data from a different author (the existing liver proteome that they had in before but have now played up a bit more). Neither of these data sets has sufficient data in order to get statistically meaningful data to strongly support their hypothesis. They then present this data in figure 1 and 2 and S1 and S2 and analyze the phase and the overlap of these oscillations. Their data is consistent BUT their data is not well sampled enough for a confident argument. Proteomics data, especially phosphoproteomics data, is way too messy for a single replicate. I don't believe that they need multiple different cell types, just more biological replication.

In all honestly, I think that their hypothesis in the paper is probably right, and will be backed up by more replication. However, there is too much noise in proteomic data for a single biological replicate to be sufficient for the kinds of statements they are making about the data. I think that their figures 3-6 tell the story without the need for the omics analysis and if they do not want to, or cannot afford to, do a more detailed proteomics analysis, then they should deemphasize their data in figures 1 and 2 and refocus their paper on figures 3-6.

I would be happy to provide a thorough response to this new version when the authors are ready. I do think that it adds to the conversation about CRY in the circadian field. However, I don't feel that their omics are strong enough to stand alone in the way that they are framing them and they would need to change something in order for me to support publication."

We thank the reviewer for this further feedback. in our revised manuscript we have enacted both of their suggestions by: (1) reducing the emphasis on any specific results/analysis presented in Figures 1,2 & S1-4 that were not subsequently and independently validated in separate experiments (i.e., with sufficient biological replicates); and by (2) performing the validation experiment outlined in our previous response to reviewers, i.e., 4 independently-derived biological replicates from each genotype sampled after 36 and 48 h under constant conditions.

The results of this validation experiment agree with the major findings of the 3 day proteomics experiment, i.e., more differences in protein composition overall between genotypes than variation over time within each genotype; differing identities of most proteins whose abundance changed over time between genotypes, association between change in abundance and change in temporal variation etc. These data are presented in the new Figure 3. Compared with the original 3 day study, we did not observe an increased number of proteins whose abundance changed over time in CKO compared with WT cells. However, this is very likely attributable to the increased variance in the abundance of proteins that change over time between the 4 different CKO biological replicates compared with between the WT replicates (Figure 3E). Because this experiment provided no direct evidence for significantly increased numbers of time-dependent abundance changes in CKO cells, we have deemphasised this element of the 3 day time course analysis in the revised manuscript. Instead we have stated that "at least as many" proteins are subject to temporal variation in CKO as WT cells, unless referring specifically to the 3 day time course.

More generally, and in addition to the changes made after both reviewers' previous comments, we have made further and rather substantial changes to the manuscript in order to better focus on what we think to be the most important and robust findings. We thank the reviewer for encouraging us on the need for clear communication of the most pertinent experimental observations. Specifically, we have:

- Revised the title to "CRYPTOCHROMES promote daily protein homeostasis", for which there surely is now overwhelming supporting evidence.

- Revised the abstract to reflect changes in the focus of the text and figures.

- Added an entirely new figure 3 and accompanying results section that provides ample validation of the key findings from the first 3-day proteomics study, as the reviewer requested.

- Reformatting Figure S2B/S3B to allow comparisons to be easily made with the new Figure 3G, i.e., both experiments found that genotype-dependent differences in proteome composition are much greater than variation in proteome composition over time within a genotype.

- Emphasised that the findings of the first 3-day proteomics cannot, alone, provide support for any specific genotype-dependent differences in proteome composition.

- Removed several paragraphs from the results and discussion section that concerned the 3 day proteomics experiment. A severely truncated and heavily revised version of this text can now be found in the legends of Figures S2-4. In the interests of transparency, full disclosure and open communication, we decided it would not be appropriate to remove these analyses entirely. They thus remain available for the very interested reader, though the text makes clear that any findings that we did not pursue experimentally require further validation before they can be accepted.

- Further emphasised the scope of this study, which essentially just asks why circadian timing is less robust in CRY-deficient cells; to do this we began by testing an extension of the prevailing hypothesis: that CRY proteins are essential for temporal regulation of cellular proteomes. Analysis of the 3 day proteomics data inform the subsequent hypothesis that we go on to test by many independent

methods. Our study does not address the basis of circadian timekeeping, nor does it address all the pathways by which proteome imbalance arises in CRY-deficient cells, which this is bound to be multifactorial and beyond the scope of this investigation.

- Further emphasised that we do not claim any biological relevance for temporal variation in protein abundance, which has been assumed previously elsewhere. Indeed, if anything, our observations support the opposite conclusion: that daily variation in proteome composition is disadvantageous, and that circadian regulation helps to prevent it.

We have highlighted the major changes to the revised text, and the reviewer will note the many additional caveats that we have added throughout the manuscript, wherever an interpretation is made. Ultimately the data are the data and need to be communicated at this stage because, irrespective of what we think they mean, there is no precedent for these findings; which are supported by multiple independent methods. We are as confident as one ever can be about their reproducibility and simply suggest what we believe to be the most parsimonious interpretation for their meaning. We hope the reviewer is satisfied by the extensive changes we have made to the manuscript, which now provides much more validation and exploration of physiological consequence than in the original submission.

1st Revision - Editorial Decision 3rd Nov 2021

Dear Dr. O'Neill,

Thank you for the submission of your revised manuscript to The EMBO Journal. We now consider that you have properly dealt with the referees' concerns and your manuscript is almost ready for publication once a minor detail has been addressed:

Please provide the paper's synopsis composed of:
- A short 'blurb' text summarizing in two sentences the study (max. 250 characters). Add as well three to four 'bullet points' highlighting the main findings. Bullet points and standfirst text should be submitted as a separate manuscript file in LaTeX, RTF or MS Word format.
- A "synopsis image", which can be used as a "visual title" for the synopsis section of your paper. The image should be PNG or JPG format with pixel dimensions of 550 x 300-600 (width x height).

Please let me know if you have any further questions regarding any of these points. Thank you again for giving us the chance to consider your manuscript for The EMBO Journal. I look forward to receiving the final version.

Yours sincerely,

David del Alamo
Editor
The EMBO Journal

Further information is available in our Guide For Authors: https://www.embopress.org/page/journal/14602075/authorguide

----

***
Rev_Com_number: RC-2021-00727
New_manu_number: EMBOJ-2021-108883R
Corr_author: O'Neill
Title: CRYPTOCHROMES promote daily protein homeostasis

The authors performed the requested editorial changes.

2nd Revision - Editorial Decision 9th Nov 2021

Dear Dr. O'Neill,

I am pleased to inform you that your manuscript has been accepted for publication in the EMBO Journal.

Please note that it is EMBO Journal policy for the transcript of the editorial process (containing referee reports and your response letter) to be published as an online supplement to each paper. If you do NOT want this, you will need to inform the Editorial Office via email immediately. More information is available here: https://emboj.embopress.org/about#Transparent_Process

Your manuscript will be processed for publication in the journal by EMBO Press. Manuscripts in the PDF and electronic editions of The EMBO Journal will be copy edited, and you will be provided with page proofs prior to publication. Please note that supplementary information is not included in the proofs.

Should you be planning a Press Release on your article, please get in contact with embojournal@wiley.com as early as possible, in order to coordinate publication and release dates.

If you have any questions, please do not hesitate to call or email the Editorial Office. Thank you for your contribution to The EMBO Journal.

Yours sincerely,

David del Alamo
Editor
The EMBO Journal

***

Rev_Com_number: RC-2021-00727
New_manu_number: EMBOJ-2021-108883R1
Corr_author: O'Neill
Title: CRYPTOCHROMES promote daily protein homeostasis

EMBO PRESS

YOU MUST COMPLETE ALL CELLS WITH A PINK BACKGROUND ↓

PLEASE NOTE THAT THIS CHECKLIST WILL BE PUBLISHED ALONGSIDE YOUR PAPER

Corresponding Author Name: John O'Neill
Journal Submitted to: EMBO Journal
Manuscript Number: EMBOJ-2021-108883

Reporting Checklist For Life Sciences Articles (Rev. June 2017)

This checklist is used to ensure good reporting standards and to improve the reproducibility of published results. These guidelines are consistent with the Principles and Guidelines for Reporting Preclinical Research issued by the NIH in 2014. Please follow the journal's authorship guidelines in preparing your manuscript.

A- Figures
1. Data
The data shown in figures should satisfy the following conditions:

- the data were obtained and processed according to the field's best practice and are presented to reflect the results of the experiments in an accurate and unbiased manner.
- figure panels include only data points, measurements or observations that can be compared to each other in a scientifically meaningful way.
- graphs include clearly labeled error bars for independent experiments and sample sizes. Unless justified, error bars should not be shown for technical replicates.
- if n< 5, the individual data points from each experiment should be plotted and any statistical test employed should be justified
- Source Data should be included to report the data underlying graphs. Please follow the guidelines set out in the author ship guidelines on Data Presentation.

2. Captions

Each figure caption should contain the following information, for each panel where they are relevant:

- a specification of the experimental system investigated (eg cell line, species name).
- the assay(s) and method(s) used to carry out the reported observations and measurements
- an explicit mention of the biological and chemical entity(ies) that are being measured.
- an explicit mention of the biological and chemical entity(ies) that are altered/varied/perturbed in a controlled manner.

- the exact sample size (n) for each experimental group/condition, given as a number, not a range;
- a description of the sample collection allowing the reader to understand whether the samples represent technical or biological replicates (including how many animals, litters, cultures, etc.).
- a statement of how many times the experiment shown was independently replicated in the laboratory.
- definitions of statistical methods and measures:
  - common tests, such as t-test (please specify whether paired vs. unpaired), simple χ2 tests, Wilcoxon and Mann-Whitney tests, can be unambiguously identified by name only, but more complex techniques should be described in the methods section;
  - are tests one-sided or two-sided?
  - are there adjustments for multiple comparisons?
  - exact statistical test results, e.g., P values = x but not P values < x;
  - definition of 'center values' as median or average;
  - definition of error bars as s.d. or s.e.m.

Any descriptions too long for the figure legend should be included in the methods section and/or with the source data.

In the pink boxes below, please ensure that the answers to the following questions are reported in the manuscript itself. Every question should be answered. If the question is not relevant to your research, please write NA (non applicable). We encourage you to include a specific subsection in the methods section for statistics, reagents, animal models and human subjects.

USEFUL LINKS FOR COMPLETING THIS FORM

http://www.antibodypedia.com
http://1degreebio.org
http://www.equator-network.org/reporting-guidelines/improving-bioscience-research-repor
http://grants.nih.gov/grants/olaw/olaw.htm
http://www.mrc.ac.uk/Ourresearch/Ethicsresearchguidance/Useofanimals/index.htm
http://ClinicalTrials.gov
http://www.consort-statement.org
http://www.consort-statement.org/checklists/view/32-consort/66-title
http://www.equator-network.org/reporting-guidelines/reporting-recommendations-for-tumo
http://datadryad.org
http://figshare.com
http://www.ncbi.nlm.nih.gov/gap
http://www.ebi.ac.uk/ega
http://biomodels.net/
http://biomodels.net/miriam/
http://jjj.biochem.sun.ac.za
https://osp.od.nih.gov/biosafety-biosecurity-and-emerging-biotechnology/
http://www.selectagents.gov/

B- Statistics and general methods

Please fill out these boxes ↓ (Do not worry if you cannot see all your text once you press return)

1.a. How was the sample size chosen to ensure adequate power to detect a pre-specified effect size?	Where a specific hypothesis was tested, no explicit assumption about effect size was made beforehand since we had not performed the experiment, but typically 10% coefficients of variation are expected. For example, in Figure 7, we used n of 8 to test whether a difference in circadian period could be detected between experimental groups assuming a variance of 10% based on previous similar experiments. We knew this would be sufficient to detect a period difference between groups of 0.5h (alpha = 0.05, Power of 90%) for 10% variance within groups. We were therefore underpowered to detect small differences between groups, in order to minimise the liklihood of type 1 errors.
1.b. For animal studies, include a statement about sample size estimate even if no statistical methods were used.	See above
2. Describe inclusion/exclusion criteria if samples or animals were excluded from the analysis. Were the criteria pre-established?	One control animal that died during the experiment presented in Figure 7I-L which was not included in our analysis.
3. Were any steps taken to minimize the effects of subjective bias when allocating animals/samples to treatment (e.g. randomization procedure)? If yes, please describe.	Animals were assigned to groups by research technicians who were unaware of the hypothesis under investigation.
For animal studies, include a statement about randomization even if no randomization was used.	No specific method of randomisation was used as animals were age/gender matched and assigned to groups arbitrarily.
4.a. Were any steps taken to minimize the effects of subjective bias during group allocation or/and when assessing results (e.g. blinding of the investigator)? If yes please describe.	All such analyses were performed by two or more individuals, independently by objective methods as reported.
4.b. For animal studies, include a statement about blinding even if no blinding was done	No blinding was performed
5. For every figure, are statistical tests justified as appropriate?	Yes
Do the data meet the assumptions of the tests (e.g., normal distribution)? Describe any methods used to assess it.	Where appropriate, normality was tested using the normal distribution test of Graphpad Prism.

Is there an estimate of variation within each group of data?	Where appropriate the variation within each group is described.
Is the variance similar between the groups that are being statistically compared?	In cases where the variance between groups is not similar this is reported explicitly.

C- Reagents

6. To show that antibodies were profiled for use in the system under study (assay and species), provide a citation, catalog number and/or clone number, supplementary information or reference to an antibody validation profile. e.g., Antibodypedia (see link list at top right), 1DegreeBio (see link list at top right).	This information is included in the Materials and Methods section
7. Identify the source of cell lines and report if they were recently authenticated (e.g., by STR profiling) and tested for mycoplasma contamination.	Fibroblasts homozygous were extracted from adult mouse lung tissue within the O'Neill lab with a continuous and documented chain of custody. All cells were confirmed to be free of Mycoplasma.

* for all hyperlinks, please see the table at the top right of the document

D- Animal Models

8. Report species, strain, gender, age of animals and genetic modification status where applicable. Please detail housing and husbandry conditions and the source of animals.	C57BL/6J, male and female mice, 2-5 months of age, homozygous null or positive for Cry1 and Cry2. Mice used to derive fibroblasts were homozygous for the PER2::LUC (Yoo et al, 2004).
9. For experiments involving live vertebrates, include a statement of compliance with ethical regulations and identify the committee(s) approving the experiments.	All animal work was licensed by the Home Office under the Animals (Scientific Procedures) Act 1986, with Local Ethical Review by the Medical Research Council and the University of Cambridge, UK.
10. We recommend consulting the ARRIVE guidelines (see link list at top right) (PLoS Biol. 8(6), e1000412, 2010) to ensure that other relevant aspects of animal studies are adequately reported. See author guidelines, under 'Reporting Guidelines'. See also: NIH (see link list at top right) and MRC (see link list at top right) recommendations. Please confirm compliance.	Compliance confirmed

E- Human Subjects

11. Identify the committee(s) approving the study protocol.	N/A
12. Include a statement confirming that informed consent was obtained from all subjects and that the experiments conformed to the principles set out in the WMA Declaration of Helsinki and the Department of Health and Human Services Belmont Report.	N/A
13. For publication of patient photos, include a statement confirming that consent to publish was obtained.	N/A
14. Report any restrictions on the availability (and/or on the use) of human data or samples.	N/A
15. Report the clinical trial registration number (at ClinicalTrials.gov or equivalent), where applicable.	N/A
16. For phase II and III randomized controlled trials, please refer to the CONSORT flow diagram (see link list at top right) and submit the CONSORT checklist (see link list at top right) with your submission. See author guidelines, under 'Reporting Guidelines'. Please confirm you have submitted this list.	N/A
17. For tumor marker prognostic studies, we recommend that you follow the REMARK reporting guidelines (see link list at top right). See author guidelines, under 'Reporting Guidelines'. Please confirm you have followed these guidelines.	N/A

F- Data Accessibility

18: Provide a "Data Availability" section at the end of the Materials & Methods, listing the accession codes for data generated in this study and deposited in a public database (e.g. RNA-Seq data: Gene Expression Omnibus GSE39462, Proteomics data: PRIDE PXD000208 etc.) Please refer to our author guidelines for 'Data Deposition'.<br><br>Data deposition in a public repository is mandatory for:<br>a. Protein, DNA and RNA sequences<br>b. Macromolecular structures<br>c. Crystallographic data for small molecules<br>d. Functional genomics data<br>e. Proteomics and molecular interactions	Done
19. Deposition is strongly recommended for any datasets that are central and integral to the study; please consider the journal's data policy. If no structured public repository exists for a given data type, we encourage the provision of datasets in the manuscript as a Supplementary Document (see author guidelines under 'Expanded View' or in unstructured repositories such as Dryad (see link list at top right) or Figshare (see link list at top right).	Datasets have been deposited as specified in the Data Availability section
20. Access to human clinical and genomic datasets should be provided with as few restrictions as possible while respecting ethical obligations to the patients and relevant medical and legal issues. If practically possible and compatible with the individual consent agreement used in the study, such data should be deposited in one of the major public access-controlled repositories such as dbGAP (see link list at top right) or EGA (see link list at top right).	This study did not involve humans
21. Computational models that are central and integral to a study should be shared without restrictions and provided in a machine-readable form. The relevant accession numbers or links should be provided. When possible, standardized format (SBML, CellML) should be used instead of scripts (e.g. MATLAB). Authors are strongly encouraged to follow the MIRIAM guidelines (see link list at top right) and deposit their model in a public database such as Biomodels (see link list at top right) or JWS Online (see link list at top right). If computer source code is provided with the paper, it should be deposited in a public repository or included in supplementary information.	Source code is provided on Github, with the link in the Data Availability section

G- Dual use research of concern

22. Could your study fall under dual use research restrictions? Please check biosecurity documents (see link list at top right) and list of select agents and toxins (APHIS/CDC) (see link list at top right). According to our biosecurity guidelines, provide a statement only if it could.	No
